# Fluctuating temperature modifies heat-mortality association around the globe

**DOI:** 10.1016/j.xinn.2022.100225

**Published:** 2022-03-11

**Authors:** Yao Wu, Bo Wen, Shanshan Li, Antonio Gasparrini, Shilu Tong, Ala Overcenco, Aleš Urban, Alexandra Schneider, Alireza Entezari, Ana Maria Vicedo-Cabrera, Antonella Zanobetti, Antonis Analitis, Ariana Zeka, Aurelio Tobias, Barrak Alahmad, Ben Armstrong, Bertil Forsberg, Carmen Íñiguez, Caroline Ameling, César De la Cruz Valencia, Christofer Åström, Danny Houthuijs, Do Van Dung, Dominic Royé, Ene Indermitte, Eric Lavigne, Fatemeh Mayvaneh, Fiorella Acquaotta, Francesca de’Donato, Francesco Sera, Gabriel Carrasco-Escobar, Haidong Kan, Hans Orru, Ho Kim, Iulian-Horia Holobaca, Jan Kyselý, Joana Madureira, Joel Schwartz, Klea Katsouyanni, Magali Hurtado-Diaz, Martina S. Ragettli, Masahiro Hashizume, Mathilde Pascal, Micheline de Sousa Zanotti Stagliorio Coélho, Noah Scovronick, Paola Michelozzi, Patrick Goodman, Paulo Hilario Nascimento Saldiva, Rosana Abrutzky, Samuel Osorio, Tran Ngoc Dang, Valentina Colistro, Veronika Huber, Whanhee Lee, Xerxes Seposo, Yasushi Honda, Michelle L. Bell, Yuming Guo

**Affiliations:** 1 Department of Epidemiology and Preventive Medicine, School of Public Health and Preventive Medicine, Monash University, Melbourne 3004, Australia; 2 Climate, Air Quality Research Unit, School of Public Health and Preventive Medicine, Monash University, Melbourne 3004, Australia; 3 Department of Public Health, Environments, and Society, London School of Hygiene & Tropical Medicine, London WC1E 7HT, UK; 4 Centre for Statistical Methodology, London School of Hygiene & Tropical Medicine, London WC1E 7HT, UK; 5 Centre on Climate Change & Planetary Health, London School of Hygiene & Tropical Medicine, London WC1E 7HT, UK; 6 Shanghai Children’s Medical Centre, Shanghai Jiao Tong University, Shanghai 200025, China; 7 School of Public Health, Institute of Environment and Human Health, Anhui Medical University, Hefei 230032, China; 8 Center for Global Health, Nanjing Medical University, Nanjing 211166, China; 9 School of Public Health and Social Work, Queensland University of Technology, Brisbane 4000, Australia; 10 National Agency for Public Health of the Ministry of Health, Labour, and Social Protection of the Republic of Moldova, Chisinau MD-2009, Republic of Moldova; 11 Institute of Atmospheric Physics, Czech Academy of Sciences, Prague 141 00, Czech Republic; 12 Faculty of Environmental Sciences, Czech University of Life Sciences, Prague 165 00, Czech Republic; 13 Institute of Epidemiology, Helmholtz Zentrum München–German Research Center for Environmental Health, Neuherberg 85747, Germany; 14 Faculty of Geography and Environmental Sciences, Hakim Sabzevari University, Sabzevar 9617976487, Iran; 15 Institute of Social and Preventive Medicine, University of Bern, Bern 3012, Switzerland; 16 Oeschger Center for Climate Change Research, University of Bern, Bern 3012, Switzerland; 17 Department of Environmental Health, Harvard T.H. Chan School of Public Health, Harvard University, Boston, MA 02115, USA; 18 Department of Hygiene, Epidemiology and Medical Statistics, National and Kapodistrian University of Athens, Athens 11527, Greece; 19 Institute for Environment, Health, and Societies, Brunel University London, London UB8 3PN, UK; 20 Institute of Environmental Assessment and Water Research, Spanish Council for Scientific Research, Barcelona 08034, Spain; 21 School of Tropical Medicine and Global Health, Nagasaki University, Nagasaki 852-8521, Japan; 22 Department of Public Health and Clinical Medicine, Umeå University, Umeå 901 87, Sweden; 23 Department of Statistics and Computational Research, Universitat de València, València 46003, Spain; 24 CIBER de Epidemiología y Salud Pública (CIBERESP), Madrid 28029, Spain; 25 National Institute for Public Health and the Environment (RIVM), Centre for Sustainability and Environmental Health, Bilthoven 3720 BA, Netherlands; 26 Department of Environmental Health, National Institute of Public Health, Cuernavaca Morelos 62100, Mexico; 27 Department of Environmental Health, Faculty of Public Health, University of Medicine and Pharmacy at Ho Chi Minh City, Ho Chi Minh City 17000, Vietnam; 28 Department of Geography, University of Santiago de Compostela, Santiago de Compostela 15705, Spain; 29 Institute of Family Medicine and Public Health, University of Tartu, Tartu 50090, Estonia; 30 School of Epidemiology & Public Health, Faculty of Medicine, University of Ottawa, Ottawa, ON K1N 6N5, Canada; 31 Air Health Science Division, Health Canada, Ottawa, ON K1A 0K9, Canada; 32 Department of Earth Sciences, University of Turin, Turin 10125, Italy; 33 Department of Epidemiology, Lazio Regional Health Service, Rome 00147, Italy; 34 Department of Statistics, Computer Science, and Applications “G. Parenti”, University of Florence, Florence 50121, Italy; 35 Health Innovation Laboratory, Institute of Tropical Medicine “Alexander von Humboldt”, Universidad Peruana Cayetano Heredia, Lima 15102, Peru; 36 Scripps Institution of Oceanography, University of California San Diego, La Jolla, CA 92093, USA; 37 Department of Environmental Health, School of Public Health, Fudan University, Shanghai 200032, China; 38 Graduate School of Public Health, Seoul National University, Seoul 08826, Republic of Korea; 39 Faculty of Geography, Babeş-Bolyai University, Cluj-Napoca 400084, Romania; 40 EPIUnit – Instituto de Saúde Pública, Universidade do Porto, Porto 4050-600, Portugal; 41 Laboratório para a Investigação Integrativa e Translacional em Saúde Populacional (ITR), Porto 4050-600, Portugal; 42 Environmental Health Department, Instituto Nacional de Saúde Dr. Ricardo Jorge, Porto 4000-055, Portugal; 43 School of Population Health and Environmental Sciences, King’s College London, London WC2R 2LS, UK; 44 Swiss Tropical and Public Health Institute, Basel 4051, Switzerland; 45 University of Basel, Basel 4001, Switzerland; 46 Department of Global Health Policy, Graduate School of Medicine, The University of Tokyo, Tokyo 113-8654, Japan; 47 Santé Publique France, Department of Environmental Health, French National Public Health Agency, Saint Maurice 94 410, France; 48 Department of Pathology, Faculty of Medicine, University of São Paulo, São Paulo 05508-270, Brazil; 49 Gangarosa Department of Environmental Health, Rollins School of Public Health, Emory University, Atlanta, GA 30322, USA; 50 Technological University Dublin, Dublin D07 EWV4, Ireland; 51 INSPER, São Paulo 04546-042, Brazil; 52 Universidad de Buenos Aires, Facultad de Ciencias Sociales, Instituto de Investigaciones Gino Germani, Buenos Aires C1053ABH, Argentina; 53 Department of Environmental Health, University of São Paulo, São Paulo 01246-904, Brazil; 54 Department of Quantitative Methods, School of Medicine, University of the Republic, Montevideo 11200, Uruguay; 55 IBE-Chair of Epidemiology, Ludwig Maximilian University Munich, Munich 81377, Germany; 56 Department of Physical, Chemical, and Natural Systems, Universidad Pablo de Olavide, Sevilla 41013, Spain; 57 School of the Environment, Yale University, New Haven, CT 06511, USA; 58 Department of Occupational and Environmental Medicine, School of Medicine, Ewha Womans University, Seoul 03760, South Korea; 59 Center for Climate Change Adaptation, National Institute for Environmental Studies, Tsukuba, Ibaraki 305-8506, Japan

**Keywords:** temperature variability, heat, modification effect, mortality

## Abstract

Studies have investigated the effects of heat and temperature variability (TV) on mortality. However, few assessed whether TV modifies the heat-mortality association. Data on daily temperature and mortality in the warm season were collected from 717 locations across 36 countries. TV was calculated as the standard deviation of the average of the same and previous days’ minimum and maximum temperatures. We used location-specific quasi-Poisson regression models with an interaction term between the cross-basis term for mean temperature and quartiles of TV to obtain heat-mortality associations under each quartile of TV, and then pooled estimates at the country, regional, and global levels. Results show the increased risk in heat-related mortality with increments in TV, accounting for 0.70% (95% confidence interval [CI]: −0.33 to 1.69), 1.34% (95% CI: −0.14 to 2.73), 1.99% (95% CI: 0.29–3.57), and 2.73% (95% CI: 0.76–4.50) of total deaths for Q1–Q4 (first quartile–fourth quartile) of TV. The modification effects of TV varied geographically. Central Europe had the highest attributable fractions (AFs), corresponding to 7.68% (95% CI: 5.25–9.89) of total deaths for Q4 of TV, while the lowest AFs were observed in North America, with the values for Q4 of 1.74% (95% CI: −0.09 to 3.39). TV had a significant modification effect on the heat-mortality association, causing a higher heat-related mortality burden with increments of TV. Implementing targeted strategies against heat exposure and fluctuant temperatures simultaneously would benefit public health.

## Public summary

• Increased temperature variability (TV) poses a greater mortality risk due to heat
• TV has a more profound modification effect on extreme heat-mortality association
• Strategies against heat and TV simultaneously would benefit public health

## Graphical abstract

**Figure:**
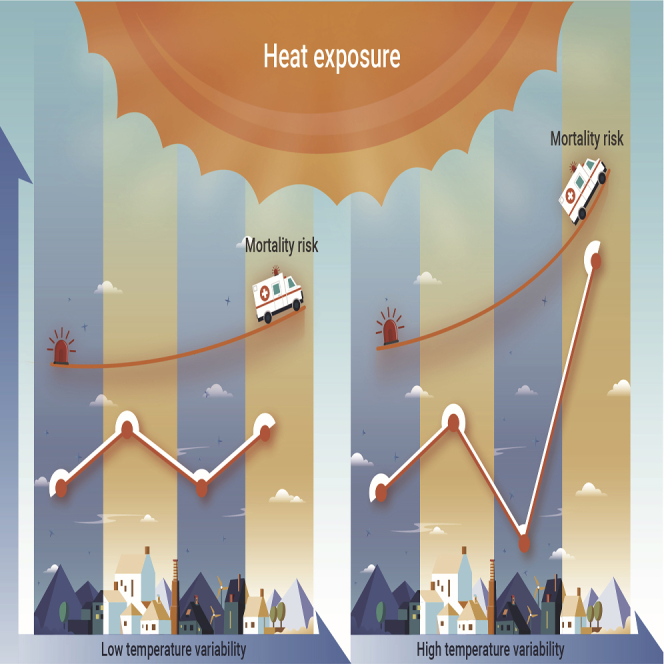

## Introduction

Global warming pervasively affects human life and undermines the years of gains in public health.^1^^,^^2^ Owing to the increasing rate of 0.2°C in temperature per decade, human-induced warming has been associated with an increase in frequency and intensity of hot days, reaching 2.9 billion additional person-days of exposure to heatwave events of vulnerable populations older than 65 years in 2019.^3^, ^4^, ^5^, ^6^ Increasing heat exposure, in turn, results in excess morbidity or mortality.^7^^,^^8^ For the past 20 years, an average of 489,075 heat-related excess deaths per year was estimated at the global level, and the heat-related excess death ratio increased from 0.83% (95% confidence interval [CI]: 0.52–1.25) in 2000–2003 to 1.04% (95% CI: 0.64–1.55) in 2016–2019.^9^ During 1991–2018, 37.0% (95% CI: 20.5–76.3) of heat-related deaths in the warm season can be attributed to human-induced heating.^10^

Another challenging issue driven by climate change is temperature variability (TV), an indicator of short-term temperature fluctuations or stability. Previous studies have projected an increasing trend of TV in some regions, in particular in tropical countries.^11^^,^^12^ Extensive studies have established evidence of the health effects of TV, showing a significant association between TV and mortality in many parts of the world and substantial public health burden stemming from TV.^13^^,^^14^ Traditionally, studies of the health effects of TV have controlled for mean temperature as a confounder.^13^^,^^15^^,^^16^ Based on similar biological mechanisms underlying the health effects of TV and heat exposure, it is worth investigating whether there exists a synergistic effect between them.^17^ Previous studies observed season-differentiated effects of TV on mortality, which suggests potential effect modification of mean temperature on TV-related mortality.^13^^,^^18^ However, to the best of our knowledge, few studies have examined whether TV modifies the heat-mortality association. For example, the temperatures fluctuations from 5°C to 25°C and from 13°C to 17°C represent the same mean temperature of 15°C, but their effects on mortality are very likely to be different. Assessing heat-mortality association without considering the modification of TV may fail to recognize the most severe weather in relation to heat and to implement an effective early warning system.

In this study, using data from the Multi-Country Multi-City (MCC) Collaborative Research Network, we systematically evaluated the contribution of TV to the heat-mortality association in 717 locations across 36 countries over the period 1972–2018. Through this study, we aimed to provide a more complete picture of the TV-differentiated heat-related mortality burden and to provide scientific evidence that could improve the sensitivity of current heat-health warning systems in hot temperatures with dramatic temperature fluctuations.

## Results

The descriptive statistics from each country are shown in Table 1. A total of 36.42 million deaths from total or non-external causes were identified during the warm season from 1972 to 2018 (Table 1). On average, the median daily TV across 717 locations was 5.8°C (25th–75th percentile: 4.9–6.7). The average daily mean temperature under each quartile of TV was 20.6°C (Q1), 21.7°C (Q2), 22.3°C (Q3), and 23.0°C (Q4) (Table 1). The overall correlation coefficient between daily mean temperature and TV was 0.10 (Table S2). The summary descriptions of the daily mean temperature and TV for each location are shown in Tables S3–S5.

**Table 1:** Mortality data and description of daily TV and mean temperature in each stratum (from Q1 to Q4) of TV in 717 locations from 36 countries during the warm season

Country	No. cities	Total death (thousands)	Temperature variability (°C)	Mean temperature (°C)				
			Median (P_25_–P_75_)	Q1	Q2	Q3	Q4	Difference between Q4 and Q1 of TV
Argentina	3	199	7.1 (6.1–8.1)	22.3	23.6	24.2	24.8	2.4
Australia	3	348	4.5 (3.7–5.6)	21.2	21.7	22.4	23.2	2.0
Brazil	18	1,064	5.5 (4.8–6.2)	25.0	25.8	26.2	26.5	1.5
Canada	26	1,135	6.4 (5.3–7.6)	16.4	17.3	17.7	18.5	2.1
China	12	284	5.2 (4.3–6.2)	24.1	25.2	25.6	25.7	1.6
Colombia	5	291	5.5 (4.9–6.1)	21.9	22.4	22.7	23.0	1.1
Costa Rica	1	10	5.5 (4.9–6.2)	23.0	23.3	23.5	23.6	0.6
Czech Republic	4	227	4.3 (3.3–5.2)	14.3	16.5	18.5	20.1	5.8
Ecuador	2	33	4.9 (4.2–5.7)	21.0	21.4	21.6	21.7	0.7
Estonia	5	46	5.7 (4.4–7.0)	14.0	14.9	15.9	17.1	3.2
France	18	513	5.9 (4.8–7.1)	17.6	18.6	19.7	21.4	3.8
Germany	12	974	5.9 (4.7–7.3)	14.8	16.5	18.2	20.7	6.0
Greece	1	82	5.3 (4.7–5.9)	25.5	26.6	27.9	29.1	3.7
Guatemala	1	21	5.0 (4.5–5.8)	19.8	20.5	20.7	20.9	1.1
Iran	1	41	8.7 (7.9–9.6)	25.2	26.1	26.2	26.0	0.8
Ireland	6	317	4.4 (3.7–5.2)	14.0	14.1	14.1	14.6	0.6
Italy	17	246	4.6 (4.0–5.4)	22.5	23.6	24.1	24.5	2.0
Japan	47	12,049	4.7 (3.9–5.5)	23.0	24.4	25.0	24.6	1.6
Mexico	10	757	7.5 (6.3–8.6)	21.3	22.7	23.5	24.1	2.8
Moldova	4	19	7.2 (6.1–8.4)	18.2	20.2	21.3	22.7	4.5
Netherland	5	142	5.5 (4.4–6.8)	15.4	16.0	16.8	18.9	3.6
Panama	1	2	4.7 (4.0–5.5)	28.1	28.9	29.2	28.7	0.5
Peru	18	174	6.6 (5.8–7.5)	19.7	20.2	20.2	20.1	0.4
Portugal	5	499	7.6 (6.3–8.9)	19.0	20.6	21.9	24.0	5.0
Puerto Rico	1	8	3.9 (3.6–4.3)	28.2	28.1	28.1	28.4	0.2
Romania	8	300	7.4 (6.3–8.5)	17.9	20.0	21.2	22.4	4.5
South Africa	52	2,148	7.8 (6.6–8.9)	21.0	22.2	22.7	23.1	2.0
South Korea	36	967	4.9 (3.9–6.0)	22.9	23.8	23.9	22.7	−0.2
Spain	45	830	7.7 (6.6–8.7)	19.4	21.5	22.6	23.8	4.4
Sweden	3	220	4.7 (3.6–6.0)	14.7	15.6	16.4	18.3	3.6
Switzerland	8	75	5.5 (4.3–6.5)	15.2	17.1	18.7	20.6	5.4
Thailand	62	571	5.5 (4.8–6.4)	28.0	28.8	29.3	29.6	1.6
UK	65	1,784	5.1 (4.1–6.3)	15.0	15.4	15.7	16.6	1.6
Uruguay	1	45	4.9 (3.6–5.9)	21.4	23.8	25.2	25.9	4.5
USA	209	9,968	7.0 (6.1–7.9)	22.1	22.9	23.1	23.0	0.9
Vietnam	2	38	5.5 (4.9–6.0)	28.5	29.3	29.6	30.2	1.6
Total	717	36,424	5.8 (4.9–6.7)	20.6	21.7	22.3	23.0	2.4

IQR = interquartile range; Q1 = the 1st quartile; Q2 = the 2nd quartile; Q3 = the 3rd quartile; Q4 = the 4th quartile; P_25_ = the 25th percentile; P_75_ = the 75th percentile; TV = temperature variability.

**Figure 1:**
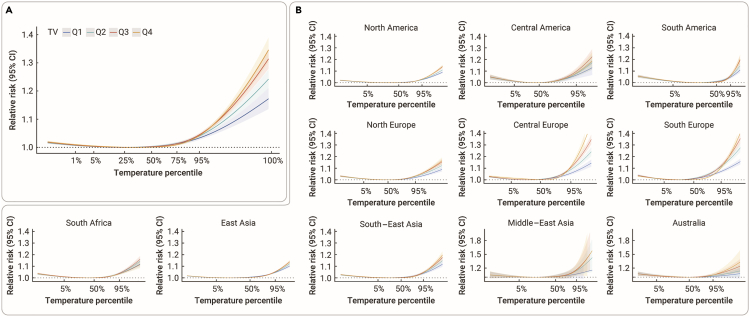
Overall cumulative exposure-response associations by temperature variability (A) Overall exposure-response curves between daily mean temperature and mortality in the warm season, stratified by quartiles of TV. (B) Regional exposure-response curves between daily mean temperature and daily mortality in the warm season, stratified by quartiles of TV. Shaded areas indicate the 95% CI. Definition of abbreviations:Q1 = the 1st quartile; Q2 = the 2nd quartile; Q3 = the 3rd quartile; Q4 = the 4th quartile; TV = temperature variability.

Figure 1 shows exposure-response curves between daily mean temperature and mortality in warm season. In general, J-shaped associations between daily mean temperature and mortality were found under different groups of TV, with the risks increasing dramatically at extreme hot temperatures (Figure 1A). The MMTs for different quartiles of TV were 17.62°C (Q1), 17.62°C (Q2), 18.39°C (Q3), and 19.80°C (Q4). The difference in four curves was tested as statistically significant. Regional exposure-response curves indicate potential geographical patterns in the modification effect of TV on the heat-mortality association (Figure 1B). In general, higher mortality risks were observed for higher TV levels across different regions. Southern Europe and central Europe generated a greater difference in heat-related mortality risks between Q1 and Q4 of TV. For most countries, the country-specific curve demonstrated increased mortality risks as TV rose (Figure S1).

**Figure 2:**
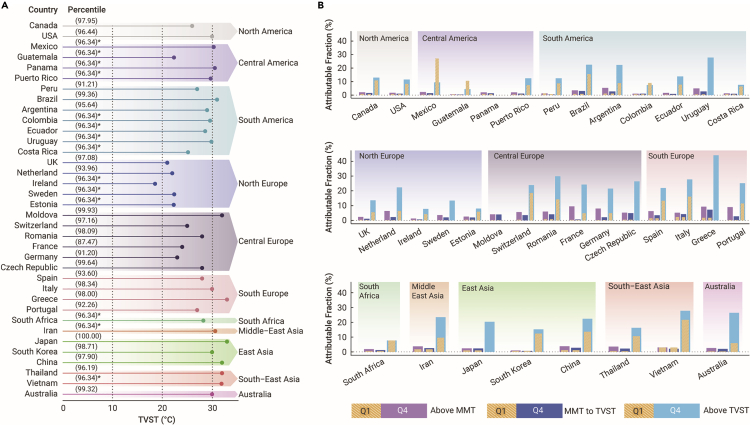
Figure 2 Fractions of all-cause mortality attributable to heat exposure by temperature variability (A) TVST identified for each country for TV. ∗For countries without identifiable TVST, the quantile threshold of the 96.34th percentile (the average of all identifiable country-specific TVSTs) in the temperature distribution for each country was used. (B) Comparison of AFs of mortality due to heat exposure for Q1 and Q4 of TV in each country, stratified by TVST. Yellow bars: the AFs for the Q1 of TV; purple bars: the AFs of mortality due to heat exposure above MMT for the Q4 of TV; dark blue bars: the AFs of mortality due to heat exposure from MMT to TVST for the Q4 of TV; light blue bars: the AFs of mortality due to heat exposure above TVST for the Q4 of TV. AF = attributable fraction; MMT = minimum mortality temperature; Q1 = the 1st quartile; Q4 = the 4th quartile; TV = temperature variability; TVST = temperature variability sensitive heat threshold.

From the Q1 to the Q4 of TV, there was an increasing trend of AF due to heat exposure, with a value of 0.70% (95% CI: −0.33 to 1.69) for Q1, 1.34% (95% CI: −0.14 to 2.73) for Q2, 1.99% (95% CI: 0.29–3.57) for Q3, and 2.73% (95% CI: 0.76–4.50) for Q4 (Table 2). The corresponding attributable deaths are shown in Table S6. Central Europe had the highest AFs for Q4 of TV among all of the regions. The country-specific AFs stratified by TV groups are shown in Table S7. Dividing the AF according to the country-specific TVST (an average of the 96.34th percentile across all of the countries), temperatures between MMT and TVST were responsible for a small fraction, increasing from 0.66% (95% CI: −0.35 to 1.62) for Q1 to 1.83% (95% CI: 0.17–3.37) for Q4 of TV. AF for temperatures above TVST changed dramatically, with increasing AFs of 8.61% (95% CI: −1.82 to 17.49), 10.48% (95% CI: −0.02 to 19.25), 12.90% (95% CI: 2.70–21.53), and 16.36% (95% CI: 5.81–24.62) for Q1–Q4 of TV, respectively (Table S8). Country-specific TVST ranged between the 87.47th percentile and the 100th percentile (Figure 2A). For most countries, AFs for the Q4 of TV were higher than the AFs for Q1 of TV, especially for temperatures above TVST (Figure 2B; Table S8).

**Table 2:** Attributable fractions of mortality due to heat exposure, stratified by quantiles of TV in each region

Region	Attributable fraction (%)			
	Q1	Q2	Q3	Q4
North America	0.71 (−0.64 to 2.00)	1.07 (−0.52 to 2.56)	1.45 (−0.22 to 3.00)	1.74 (−0.09 to 3.39)
Central America	0.22 (−0.31 to 0.74)	0.42 (−0.46 to 1.25)	1.37 (−0.33 to 2.95)	2.19 (−0.25 to 4.44)
South America	0.82 (−0.75 to 2.32)	1.59 (−0.65 to 3.67)	2.21 (0.04–4.21)	2.98 (0.20–5.46)
Northern Europe	0.17 (−0.34 to 0.67)	0.44 (−0.49 to 1.32)	0.76 (−0.27 to 1.72)	2.52 (0.52–4.31)
Central Europe	0.26 (−0.30 to 0.80)	1.08 (−0.22 to 2.29)	2.41 (0.55–4.13)	7.68 (5.25–9.89)
Southern Europe	1.19 (0.07–2.24)	2.95 (0.99–4.76)	4.09 (1.76–6.23)	7.34 (4.17–10.20)
South Africa	0.44 (−0.85 to 1.68)	0.82 (−0.98 to 2.51)	1.22 (−1.06 to 3.32)	1.79 (−1.60 to 4.72)
Middle East Asia	1.65 (−1.76 to 4.77)	3.64 (−0.38 to 7.33)	4.23 (0.37–7.75)	3.73 (0.26–6.89)
East Asia	0.87 (0.03–1.66)	1.70 (0.41–2.92)	2.47 (0.97–3.87)	2.29 (0.94–3.54)
Southeast Asia	0.55 (−0.83 to 1.91)	1.16 (−1.68 to 3.87)	2.22 (−1.49 to 5.62)	3.56 (−0.87 to 7.36)
Australia	0.25 (−0.41 to 0.86)	0.69 (−0.33 to 1.64)	1.19 (−0.18 to 2.49)	2.62 (0.70–4.39)
International	0.70 (−0.33 to 1.69)	1.34 (−0.14 to 2.73)	1.99 (0.29–3.57)	2.73 (0.76–4.50)

Definition of abbreviations: Q1 = the 1st quartile; Q2 = the 2nd quartile; Q3 = the 3rd quartile; Q4 = the 4th quartile; TV = temperature variability.

In the sensitivity analyses, our results were robust. The same patterns were observed after changing the length of exposure to TV (Figures S2–S5; Tables S9–S12) and after adding separate predictors in meta-regression with BLUP (Table S13). Using incremental lag periods, shortening the duration of the warm season, and adding relative humidity to the model, the modification effect of TV still existed (Tables S14–S16). The AFs changed slightly after using different methods to handle missing values (Table S17).

## Discussion

Our study showed that heat exposure together with high TV could significantly increase the mortality risk in the warm season. We saw an upward trend in premature death due to heat exposure with the increase in TV. For temperatures higher than TVST (96.34th percentile on average), AF showed a greater difference across 4 TV groups. The TV-modified heat-mortality burden showed disparate geographical variations.

The physiological mechanisms that explain the synergistic effects of TV and heat exposure on mortality are not yet clearly defined. However, there are several ways in which the two exposures may interact. When exposed to heat, people expend more of their reserves on thermoregulation to respond to heat.^19^ This process involves elevations in heart rate and blood pressure, vasodilatation to transfer heat to the skin, and respiration to lose heat with the expired air.^20^, ^21^, ^22^ Physiological adaptation to higher temperatures takes time. If the temperature suddenly changes in a short period of time, then people may have difficulty with internal thermoregulation, resulting in inflammatory responses and coagulation abnormalities induced by heat stress.^23^ In addition, sudden temperature changes may also activate bronchopulmonary vagal afferent nerves and the inflammatory response.^24^ For people with underlying conditions (e.g., preexisting illness, chronic diseases, poor fitness level), heat exposure may place extra pressure on the cardiovascular and respiratory systems,^25^ especially when temperature changes dramatically in a short period. Consequently, heat-related deaths may occur.

Our findings are generally consistent with previous studies focusing on TV or heat, indicating that both heat exposure and TV were positively associated with mortality.^13^^,^^26^^,^^27^ Some studies have explored the modification effect of temperature on the association between mortality and diurnal temperature range (DTR) or TV.^17^^,^^28^, ^29^, ^30^ A study in Japan showed a higher risk of cardiovascular mortality associated with TV during extremely hot days in comparison with extremely cold days.^17^ Another study in England and Wales showed a J-shaped curve of the relationship between percentiles of DTR and mortality in the warm season, while an inverted-V-shaped association was observed during the cold season.^28^ Although many studies have quantified the mortality burden associated with heat exposure, to the best of our knowledge, none of them explored the potential heterogeneity attributable to TV.^31^, ^32^, ^33^ Without taking TV into account when assessing the association between heat exposure and mortality, the heat-related mortality burden may be underestimated.

Some heterogeneity across regions was also found. For countries such as Guatemala and Colombia, temperatures above TVST together with low TV showed a higher mortality burden. The potential reasons for these results may derive from the low variation in TV and thus a small difference between Q1 and Q4 of TV. TV can be affected by many factors, such as greenness, soil moisture, and precipitation.^34^^,^^35^ For example, vegetation removal and soil aridation would act to increase daily temperature fluctuation with more rainfall.^34^ Temperate desert steppe generally has comparable warming effects on T_min_ and T_max_, while temperate meadow and temperate steppe may have larger cooling effects on T_max_ than T_min_.^35^ In addition, population aging may mediate the association. The variation in heat exposure is caused by the differences in both vulnerable populations (in particular, the elderly) and temperature across regions.^36^ For the countries such as South Africa (7.7%) and Cambodia (6.8%), the vulnerable population aged 60 or older accounted for only a small proportion of total population, lower than the global average level of 12.3% in 2015.^37^ The lower the fraction of older persons, the less sensitivity to heat exposure for the whole country, and the lower the mortality burden attributable to heat exposure. With the deepening of the aging of society, vulnerable populations are expected to increase.^37^ Further research is warranted to explore the geographic variation in TV-differentiated heat-related mortality burden and call for targeted strategies in mitigation and adaptation against climate change.

As a response to the increasing heat conditions, heat-health warning systems are developed in some parts of the world. For example, the WHO Regional Office for Europe developed the heat-health action plans (HHAPs) in 2008, covering 35 out of 53 member states of the WHO European Region by the end of 2018.^38^ Although it is hard to assess how much heat-health prevention was associated with HHAPs, a substantial reduction in heat-related deaths was observed since the implementation of preventive measures (either HHAPs or other types of intervention).^38^, ^39^, ^40^, ^41^ However, the majority of warning systems use mean temperature or T_max_ as indices to trigger the warnings and inform the general public through the mass media without user-oriented attractive notification.^42^^,^^43^ As suggested by our findings, the health effects of heat exposure could be magnified if they are accompanied by a higher TV and the findings were consistent across countries in Europe where HHAPs are implemented,^44^ implying that it is difficult for current warning systems to obtain effective heat prevention when the TV is high.

### Strengths and limitations

This study has several strengths. First, to the best of our knowledge, this is the first study to systematically explore the modifying effects of TV on heat-related mortality risks. The assessment of TV-differentiated mortality risks associated with heat exposure provides a better understanding of heat vulnerability. When quantifying the heat-related mortality burden, the effect of TV should not be ignored. Second, this study benefits from the large-scale investigation across multiple countries and a long period of time. It enables us to provide a global vision of the heat-related mortality burden under different TV exposure and to explore potential variation in estimations in terms of socioeconomic status and climatic and geographic features. Finally, our study targeted the heat-related mortality burden more precisely by highlighting the modification effect of TV when temperatures were higher than TVST.

Several limitations should be acknowledged. As the time series design and temperature data from fixed monitoring stations were used, ecological fallacy and measurement errors in exposure seem to be inevitable. Due to the lack of data for some areas of the world, some regions contain only one country. The extrapolation and interpretation of the findings are restricted to the generalization of our results. In addition, we are unable to investigate the modification effect of TV on the association between heat exposure and age- or cause-specific mortality. As the biological mechanisms are sensitive to causes of death, further research is warranted to investigate the disease-differentiated modification effect of TV on heat-related mortality burden.

## Conclusions

Our findings, which are based on multi-country data, revealed that higher TV over a short period of time increased the mortality risks associated with heat exposure. It is imperative to raise public awareness of the potential health risks of TV. Targeted adaptation strategies against heat-related mortality burden should be implemented after taking into account the fluctuation of temperatures and geographical patterns.

## Materials and methods

### Data collection

The MCC Collaborative Research Network database (http://mccstudy.lshtm.ac.uk/) was used. Daily death counts and meteorological data, including mean temperature, maximum temperature (T_max_), minimum temperature (T_min_), and relative humidity were extracted. The *International Classification of Diseases*, 9th and 10th revisions (ICD-9 and ICD-10) codes were used to identify the causes of death. We extracted the data series on non-external causes of death (ICD-9: 0–799; ICD-10: A00–R99), or, if not available, all-cause mortality. Our analyses were restricted to locations with complete weather data in the warm season (the warmest 4 consecutive months) for at least 2 consecutive years. Detailed information on data cleaning is described in Text S1. Finally, 717 locations across 36 countries were included. Daily mortality, mean temperature, T_min_, and T_max_ data had overall missing rates of 0.14%, 0.84%, 0.86%, and 0.73%, respectively (Table S1).

### Calculation of temperature variability

TV was calculated as the standard deviation (SD) of the average of T_min_ and T_max_ for the current day and 1 day before (T_max-lag0_, T_max-lag1_, T_min-lag0_, T_min-lag1_).^13^ In the sensitivity analysis, higher lengths of exposure (0–2 days and 0–3 days) were applied.

### Statistical analysis

We used a two-stage time series design to assess the modification effect of TV on the heat-related mortality burden in the warm season. In the first stage, a generalized linear regression with the quasi-Poisson family allowing overdispersion in the death counts was applied for each location to obtain location-specific estimates for the heat-mortality association. To capture the modification effect of TV on the heat-mortality association, we introduced an interaction term between a cross-basis function of daily mean temperature and quartiles (Qs) of TV. We used relative rather than absolute levels of TV to accommodate different levels of adaptive capacity to TV across locations. The equation was as follows:

$$Y_{it}∼\text{Poisson}\left(\mu ;\theta \right)\;$$

$$\text{E}\left(Y_{it}\right)=\text{exp}\left(\alpha_{i}+\beta_{i}TV_{it}+cb\left({\text{Temp}}_{it},\text{lag}=10\right)+cb\left({\text{Temp}}_{it},\text{lag}=10\right)\times {\text{Quartile}}_{TV}+ns\left({\text{Time}}_{it},df=4/\text{year}\right)+\gamma_{i}DOW_{it}\right)$$

$$\text{VAR}\left(Y_{it}\right)=\theta \mu$$

where $Y_{it}$ is daily deaths counts in location i on day t; $TV_{it}$ is the linear function of TV; and $cb\left({\text{Temp}}_{it},\;\text{lag}=10\right)$, built by distributed-lag nonlinear models (DLNMs), is a cross-basis function of daily mean temperature featuring the nonlinear and delayed association over 10 days of lag.^45^ It incorporates a natural cubic spline function with two internal knots placed at the 50th and 90th percentiles of the location-specific temperature distributions during the warm season and a natural cubic spline function of lag days with 2 internal knots placed at equally spaced values in the log scale to capture the delayed effect of temperature.^10^ ${\text{Quartile}}_{TV}$ stands for the dummy coded categorical variable of TV groups (from Q1 to Q4). To control for unmeasured temporal trends such as seasonality and long-term trend, $ns\left({\text{Time}}_{it},df=4/\text{year}\right)$, a natural cubic function of calendar days with 4 degrees of freedom (df) per year was included. In addition, an indicator for the day of the week $\left(DOW_{it}\right)$ was adjusted in the model to control for weekly variations in risk.

In the second stage, multivariate random-effects meta-analysis without predictors was used to pool the location-specific estimates at the global, regional, and national levels. Precise estimates for each location were obtained using the best linear unbiased prediction (BLUP) estimations. BLUPs can provide more accurate estimates in locations with small daily death counts or short time series by borrowing information across locations.^46^

### Quantification of temperature-specific risks and attributable fraction

For each quartile of TV, we showed the risk over a 10-day lag period at each temperature value compared with the risk at minimum mortality temperature (MMT), at which the risk of mortality was the lowest. The statistical significance of the difference in heat-mortality risks across quartiles of TV was assessed using repeated-measures multivariate meta-analysis. Briefly, based on location-specific estimates of four quartiles of TV, we performed a random effects meta-regression with TV quartiles as the only meta-predictor to test the difference in overall exposure-response curves of heat-mortality risks across quartiles of TV. Furthermore, in each country, to identify the potential targeted percentile ranges of temperature modified by TV, we tested the statistical significance of the difference in heat-mortality risks between Q1 and Q4 of TV using a fixed-effects meta-regression model at each temperature value. The modification effect of TV was identified as being statistically significant if there was a significant difference in heat-mortality risks between Q1 and Q4 of TV. The fixed-effects model was used because these country-specific estimates were based on the same samples. The country-specific temperature percentiles above which statistically significant modification effects of TV were observed, were called TV sensitive heat thresholds (TVSTs) and used to separate components of mortality burden attributable to heat exposure.

We compared the attributable deaths and attributable fractions (AFs) associated with heat exposure above MMT for each quartile of TV. Two components of heat exposure were used to separate the overall AF: from the quantile of MMT to TVST and above TVST. Daily attributable deaths due to each component of heat were calculated using BLUP location-specific association and then summed to obtain the total attributable deaths during the study period.^47^ AF was computed by dividing the attributable deaths by the total death counts. Monte Carlo simulation (n = 1,000) was used to derive 95% CIs.

### Sensitivity analysis

We conducted several sensitivity analyses to check the robustness of our results: (1) using different lengths of exposure to TV (TV 0–2 and TV 0–3); (2) adding separately location-specific predictors (region, average mean temperature, range of mean temperature, indicators for Köppen-Geiger climatic zones, gross domestic product [GDP] per capita, latitude, and longitude) to the meta-analytical model with BLUP in the second stage to check whether the pooled estimates would change while adjusting for the effects of each predictor on the location-specific estimates; (3) choosing alternative 2 and 3 warmest consecutive months to define the warm season; (4) changing the lag days of heat from 7 to 13 days; and (5) controlling the potential effect of relative humidity using a natural cubic spline with 3 df. Finally, although the overall missing rates for mortality and temperature data were generally small (Table S1), we also performed sensitivity analyses by restricting our analyses to locations with complete data and by using complete data after imputation. Missing values in time-series data were imputed by the spline interpolation method.

R software (version 3.6.2) with packages “dlnm” (for the construction of the cross-basis functions), “mvmeta” (for meta-regression), and “imputeTS” (for spline interpolation of the time-series data) was used to perform all of the analyses. A two-sided p < 0.05 was set as statistically significant. The code is available at the personal website of the first author (Github: https://github.com/yaowu-ops/Modification-effect-of-TV-on-heat-mortality-association.git).

### Data sharing

Data were collected within the MCC Collaborative Research Network under a data-sharing agreement and cannot be made publicly available. Researchers can refer to MCC participants, who are listed as coauthors of this article, for information on accessing the data for each country.

## Lead contact websites

https://research.monash.edu/en/persons/yuming-guo

https://research.monash.edu/en/persons/shandy-li

## Supplemental information

Data S2.

Document S2. Article plus supplemental information
