# Supplementary material for: Fluctuating temperature modifies heat-mortality association around the globe
Source: Innovation (Camb). 2022 Mar 11;3(2):100225. doi: 10.1016/j.xinn.2022.100225 (PMC8942841; doi:10.1016/j.xinn.2022.100225)
Supplement: Supplementary file 1 [file mmc1.pdf]

The Innovation, Volume *3*

Supplemental Information

Fluctuating temperature modifies heat-mortality association around the globe

Yao Wu, Bo Wen, Shanshan Li, Antonio Gasparrini, Shilu Tong, Ala Overcenco, Aleš Urban, Alexandra Schneider, Alireza Entezari, Ana Maria Vicedo-Cabrera, Antonella Zanobetti, Antonis Analitis, Ariana Zeka, Aurelio Tobias, Barrak Alahmad, Ben Armstrong, Bertil Forsberg, Carmen Íñiguez, Caroline Ameling, César De la Cruz Valencia, Christofer Åström, Danny Houthuijs, Do Van Dung, Dominic Royé, Ene Indermitte, Eric Lavigne, Fatemeh Mayvaneh, Fiorella Acquaotta, Francesca de'Donato, Francesco Sera, Gabriel Carrasco-Escobar, Haidong Kan, Hans Orru, Ho Kim, Iulian-Horia Holobaca, Jan Kyselý, Joana Madureira, Joel Schwartz, Klea Katsouyanni, Magali Hurtado-Diaz, Martina S. Ragettli, Masahiro Hashizume, Mathilde Pascal, Micheline de Sousa Zanotti Stagliorio Coélho, Noah Scovronick, Paola Michelozzi, Patrick Goodman, Paulo Hilario Nascimento Saldiva, Rosana Abrutzky, Samuel Osorio, Tran Ngoc Dang, Valentina Colistro, Veronika Huber, Whanhee Lee, Xerxes Seposo, Yasushi Honda, Michelle L. Bell, and Yuming Guo

Supplemental materials and methods

Table of contents

Text S1. Information about Data collection.

Figure S1. Country-specific exposure-response curve between mean temperature and mortality in warm season, stratified by quantiles of TV. Definition of abbreviations: TV = temperature variability; Q1 = the first quartile; Q2 = the second quartile; Q3 = the third quartile; Q4 = the fourth quartile.

Figure S2. Overall exposure-response curve between mean temperature and mortality in warm season, stratified by quantiles of TV 0–2. Definition of abbreviations: TV = temperature variability; Q1 = the first quartile; Q2 = the second quartile; Q3 = the third quartile; Q4 = the fourth quartile.

Figure S3. Overall exposure-response curve between mean temperature and mortality in warm season, stratified by quantiles of TV 0–3. Definition of abbreviations: TV = temperature variability; Q1 = the first quartile; Q2 = the second quartile; Q3 = the third quartile; Q4 = the fourth quartile.

Figure S4. Country-specific exposure-response curve between mean temperature and mortality in warm season, stratified by quantiles of TV 0–2. Definition of abbreviations: TV = temperature variability; Q1 = the first quartile; Q2 = the second quartile; Q3 = the third quartile; Q4 = the fourth quartile.

Figure S5. Country-specific exposure-response curve between mean temperature and mortality in warm season, stratified by quantiles of TV 0–3. Definition of abbreviations: TV = temperature variability; Q1 = the first quartile; Q2 = the second quartile; Q3 = the third quartile; Q4 = the fourth quartile.

Table S1. Descriptive information on missing rates of mortality and temperature data at the country level.

Table S2. Correlation coefficients between daily mean temperature and temperature variability.

Table S3. Description of TV and daily mean temperature in each quartile of TV for each location included in our study.

Table S4. Description of TV 0–2 and daily mean temperature in each quartile of TV 0–2 for each location included in our study.

Table S5. Description of TV 0–3 and daily mean temperature in each quartile of TV 0–3 for each location included in our study.

Table S6. Attributable deaths of mortality due to heat exposure, stratified by quantiles of TV in each region.

Table S7. Attributable fractions of mortality due to heat exposure stratified by quantiles of TV in each country.

Table S8. Attributable fractions of mortality due to heat exposure stratified by TVST for each quartile of TV in each country.

Table S9. Attributable fractions of mortality due to heat exposure stratified by quantiles of TV 0–2 in each country.

Table S10. Attributable fractions of mortality due heat exposure stratified by quantiles of TV 0–3 in each country.

Table S11. Attributable fractions of mortality due to heat exposure stratified by TVST for each quartile of TV 0–2 in each country.

Table S12. Attributable fractions of mortality due to heat exposure stratified by TVST for each quartile of TV 0–3 in each country.

Table S13. Overall attributable fractions after adding separate predictor in meta-regression with BLUP, stratified by quartiles of TV.

Table S14. Sensitivity analyses of overall attributable fractions after changing the lag of mean temperature in the main model, stratified by quartiles of TV.

Table S15. Sensitivity analyses of overall attributable fractions using different definitions of warm season, stratified by quartiles of TV.

Table S16. Sensitivity analyses of overall attributable fractions after adjusting relative humidity in the main model using 556 cities with relative humidity data, stratified by quartiles of TV.

Table S17. Sensitivity analyses of overall attributable fractions using different complete data after removing cities with missing vlaues or after imputaion, stratified by quartiles of TV.

Text S1. Information on data collection and data cleansing

We collected data on daily mortality and mean temperature (T_{mean}) in warm season for 750 cities across 43 countries or regions from the database of the MCC Collaborative Research Network (http://mccstudy.lshtm.ac.uk/). Detailed information on the process of data collection has been described in previous publications [1]. As daily minimum temperature (T_{min}) and maximum temperature (T_{max}), the primary exposures of interest in our study, were not available for some countries, we downloaded data from official websites instead. For European countries including U.K., Finland, Germany, Spain, we use the daily T_{mean}, T_{min}, and T_{max} data (10 km × 10 km resolution) extracted from the European daily high-resolution gridded dataset of surface temperature and precipitation (E-OBS) 23.1 gridded temperature datasets (https://surfobs.climate.copernicus.eu/dataaccess/access_eobs.php). E-OBS is a European land only daily high-resolution gridded dataset of maximum, mean, and minimum surface temperatures, precipitation amounts and sea level pressure, which ensured the uniformity of the temperature data in those countries. For the U.S., the daily T_{mean}, T_{min}, and T_{max} data were obtained from a national meteorological dataset (4 km × 4 km resolution) created by Parameter–Elevation Regressions on Independent Slopes Model (PRISM) (http://prism.oregonstate.edu/explorer/). The reliability of the above datasets has been proved in the previous study, ensuring a high level of consistency between them and observations from monitoring stations [2]. For China, we obtained data on daily T_{mean}, T_{min}, and T_{max} for each location included in our study from the China Meteorological Data Sharing Service Center. For locations with more than one monitoring station, daily meteorological data were calculated by averaging measurements from all valid monitoring stations in each location.

For each location, the following data cleansing procedures were used. First, we collected locations that have daily measurements of T_{mean}, T_{min}, T_{max}, and mortality for more than 99 days of warm season per year (corresponding to 80% of the complete data in warm season), and with a coverage over 2 years. For locations with several discontinuous periods over 2 years, we separated data into different datasets according to each period. Second, we identified the type of missing values for each location. As time-series data was used in this study, two types of missing manners were identified: the lack of measurements for certain variables and a small amount of missing values scattered during the study time period of each location. If it's unavailable of measurements for certain variable in one location, we excluded this location in the first step. For the second type of missing data, there is no suggestion that item being missing is correlated with death counts or any other predictors of interest. Previous studies using the same dataset

also suggested that measurements being missing were mainly caused by data logging errors or abnormal operations of the monitoring equipment [3]. Thus, they are more likely to go missing at random. A summary of missing rates of temperature data and mortality data for each country (**Table S1**). Total mortality, mean temperature, minimum temperature and maximum temperature data have an overall missing rate of 0.13%, 0.82%, 0.84%, and 0.71%, respectively. The total missing rates were relatively small, which has non-substantial influence on our estimates. To provide more convincing evidence, we also perform sensitivity analyses by restricting our analyses to locations with complete data and by using complete data after imputation.

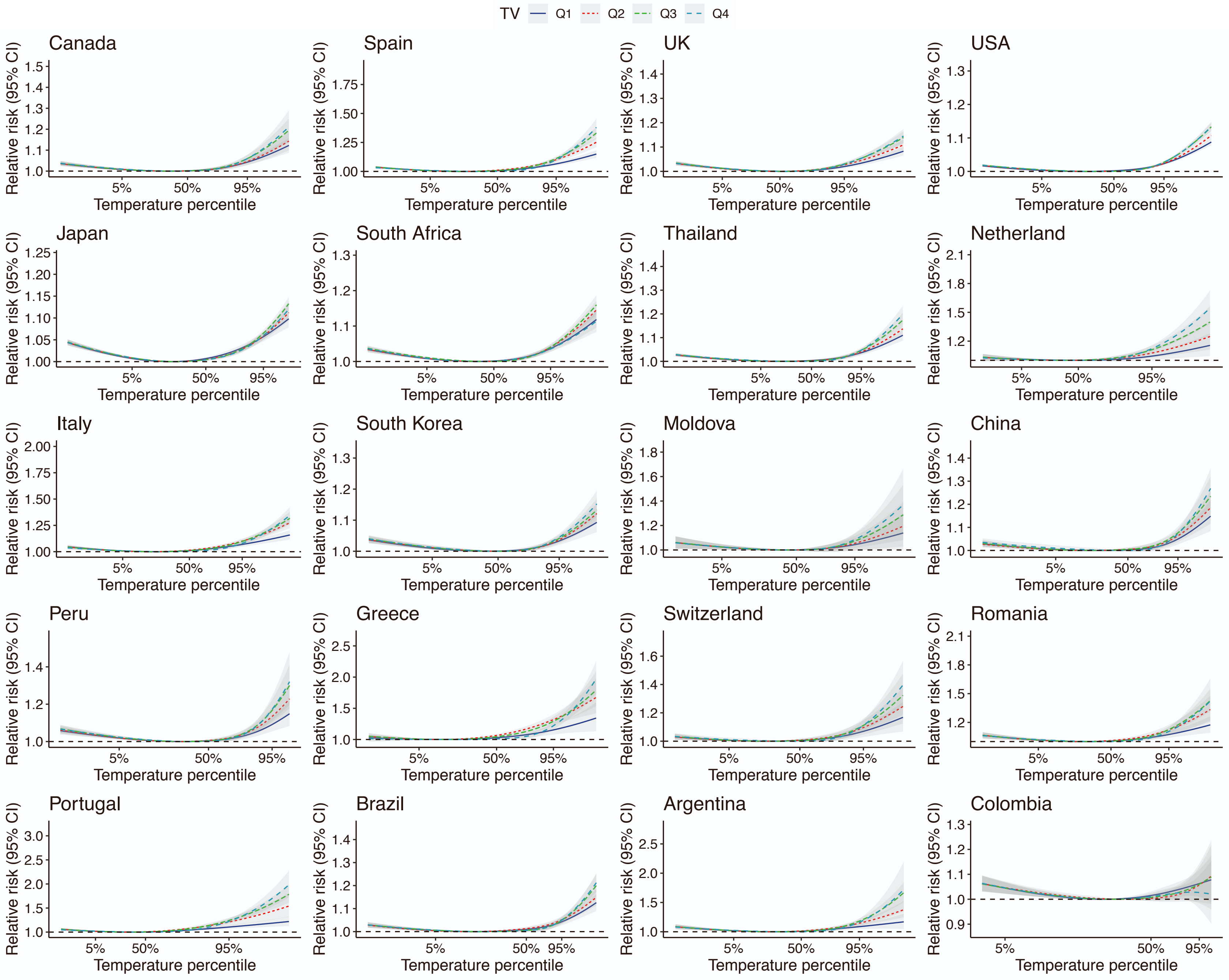
TV
Q1
Q2
Q3
Q4
Canada
Spain
UK
USA
Japan
South Africa
Thailand
Netherland
Italy
South Korea
Moldova
China
Peru
Greece
Switzerland
Romania
Portugal
Brazil
Argentina
Colombia
Relative risk (95% CI)
Temperature percentile
5%
50%
95%

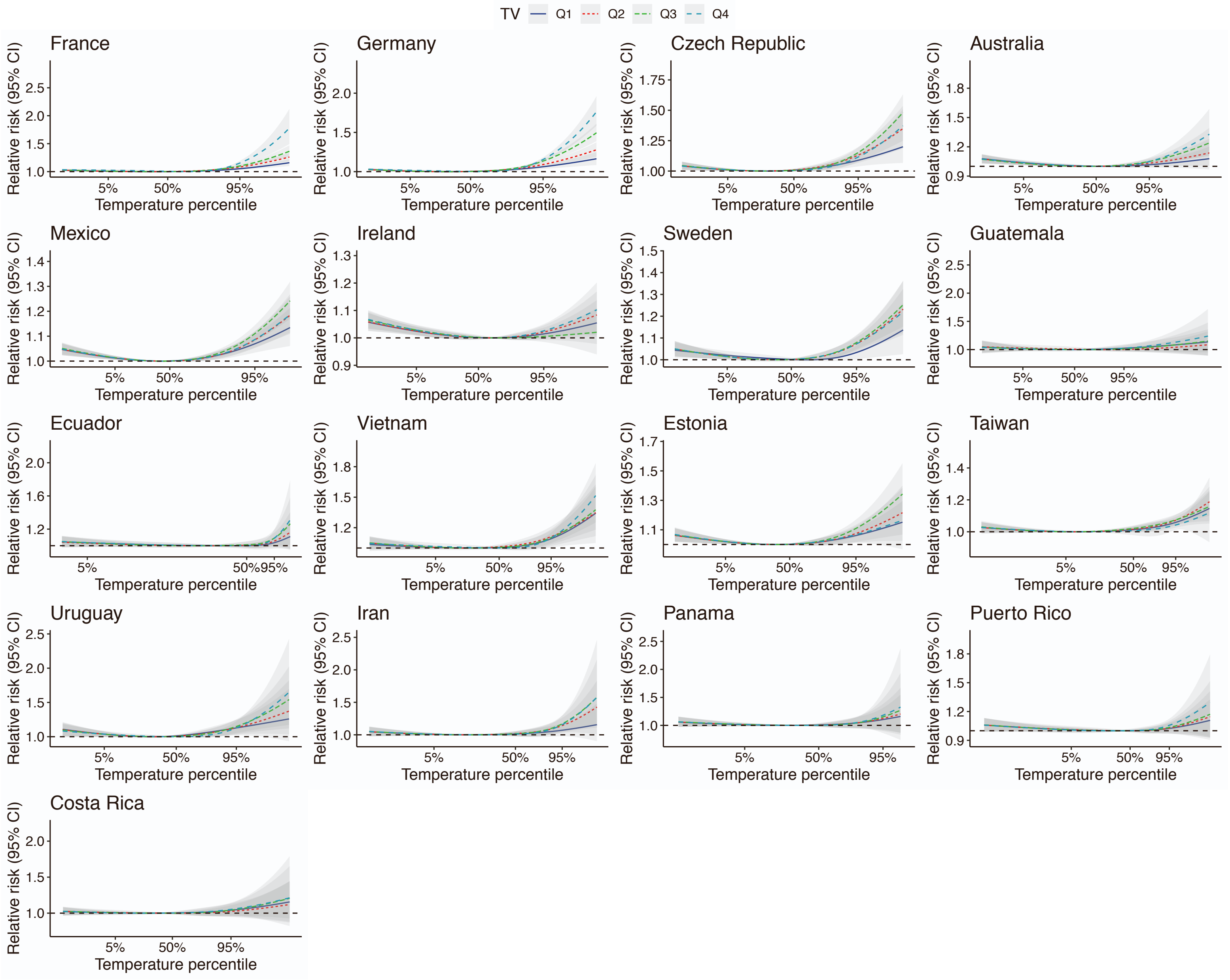

Figure S1. Country-specific exposure-response curve between mean temperature and mortality in warm season, stratified by quantiles of TV. Definition of abbreviations: TV = temperature variability; Q1 = the first quartile; Q2 = the second quartile; Q3 = the third quartile; Q4 = the fourth quartile.

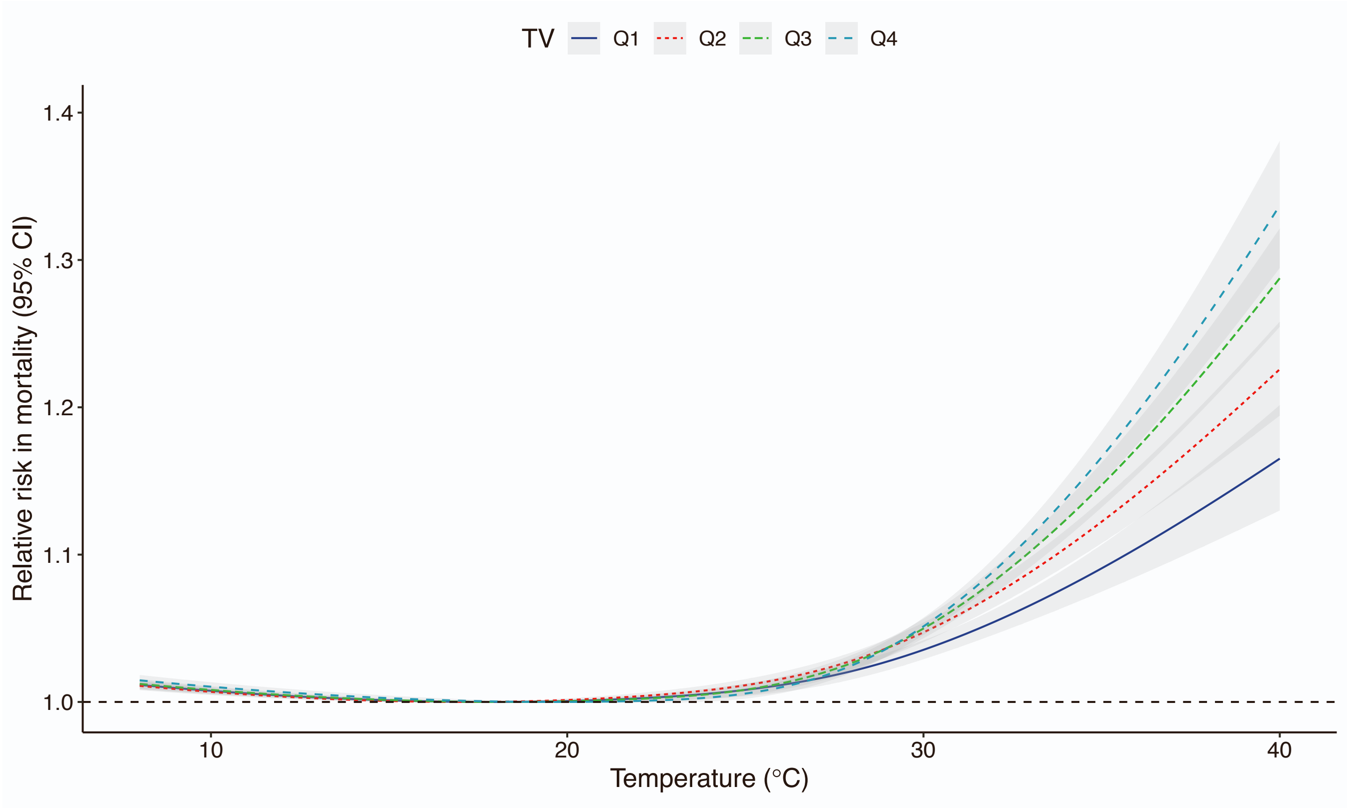

Figure S2. Overall exposure-response curve between mean temperature and mortality in warm season, stratified by quantiles of TV 0–2. Definition of abbreviations: TV = temperature variability; Q1 = the first quartile; Q2 = the second quartile; Q3 = the third quartile; Q4 = the fourth quartile.

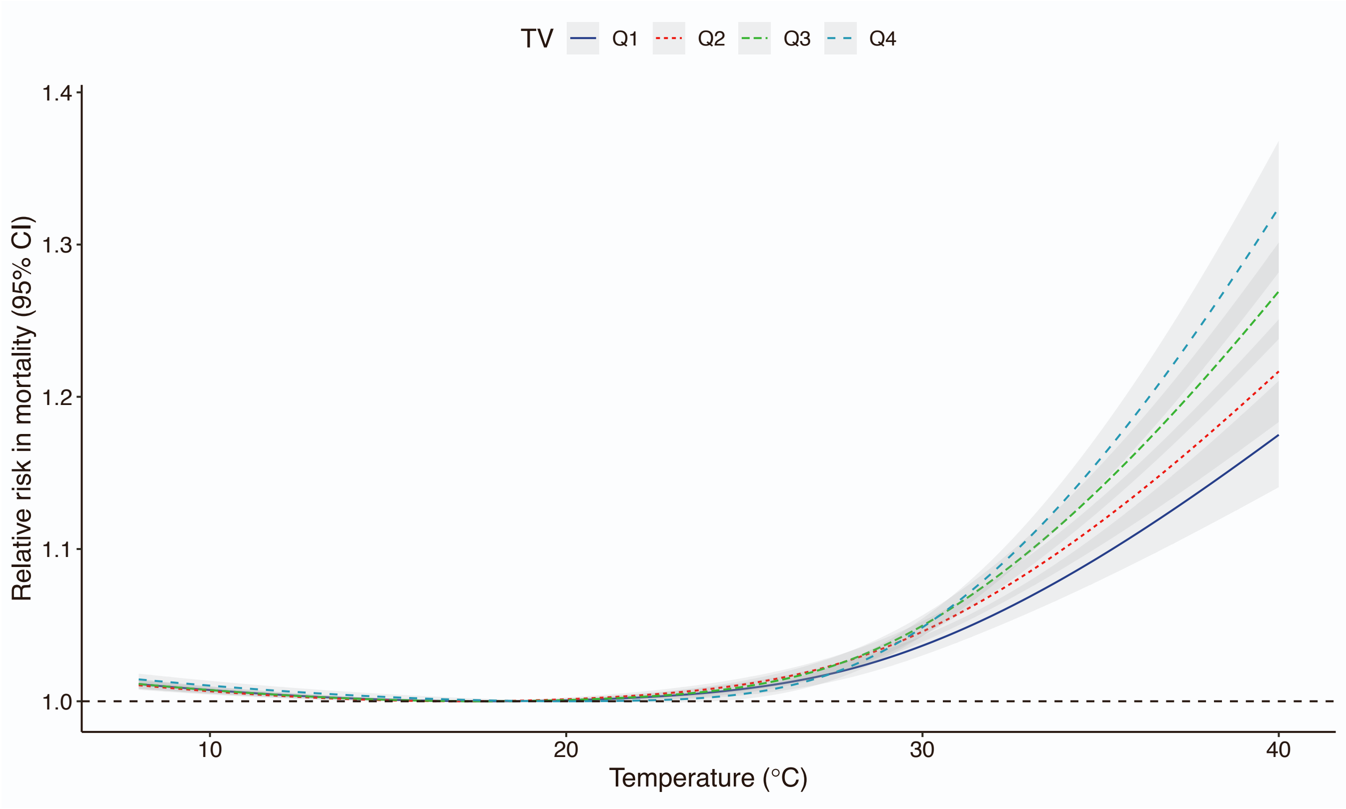

Figure S3. Overall exposure-response curve between mean temperature and mortality in warm season, stratified by quantiles of TV 0–3. Definition of abbreviations: TV = temperature variability; Q1 = the first quartile; Q2 = the second quartile; Q3 = the third quartile; Q4 = the fourth quartile.

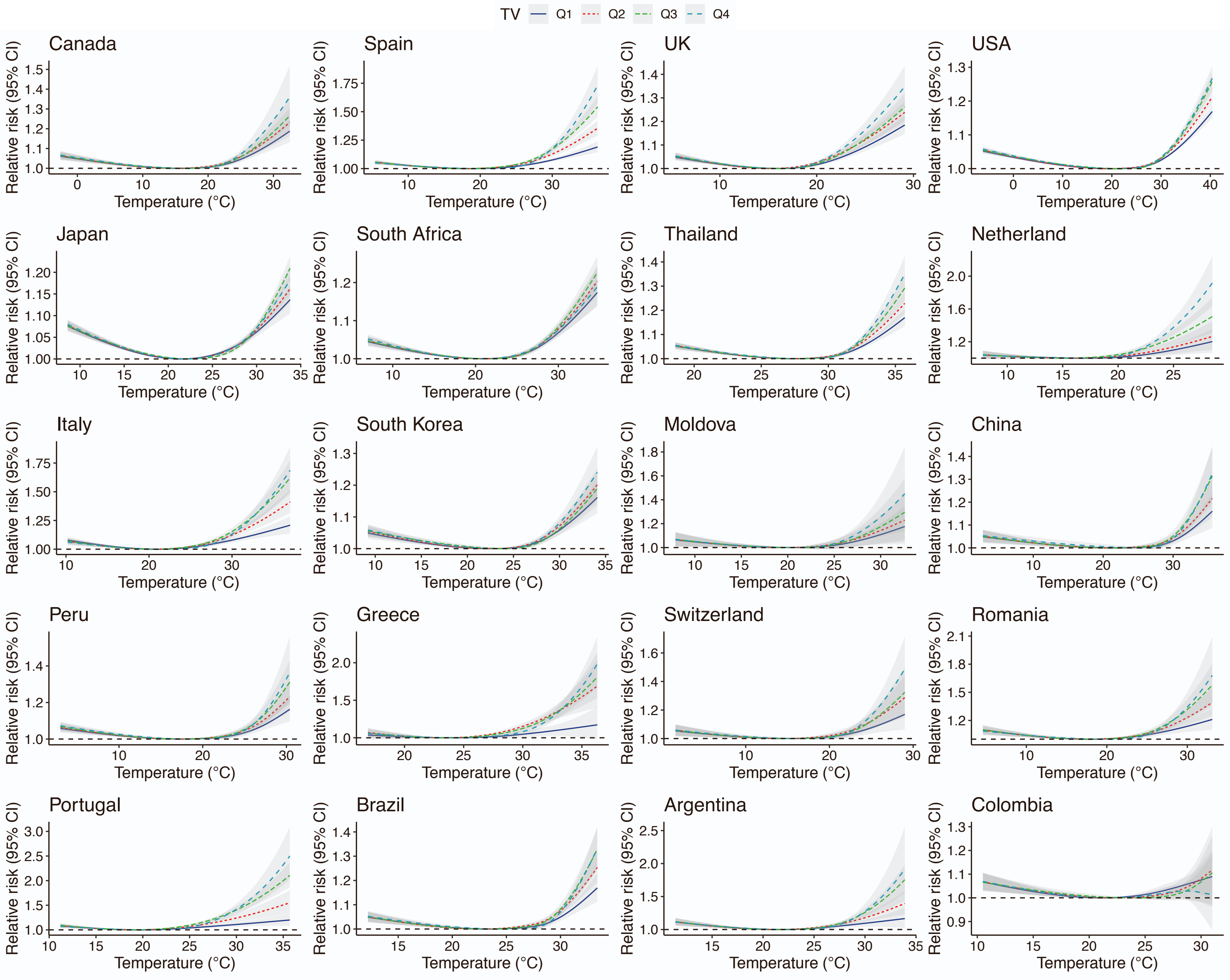

TV Q1 Q2 Q3 Q4
Canada
Spain
UK
USA
Japan
South Africa
Thailand
Netherland
Italy
South Korea
Moldova
China
Peru
Greece
Switzerland
Romania
Portugal
Brazil
Argentina
Colombia
Relative risk (95% CI)
Temperature (°C)

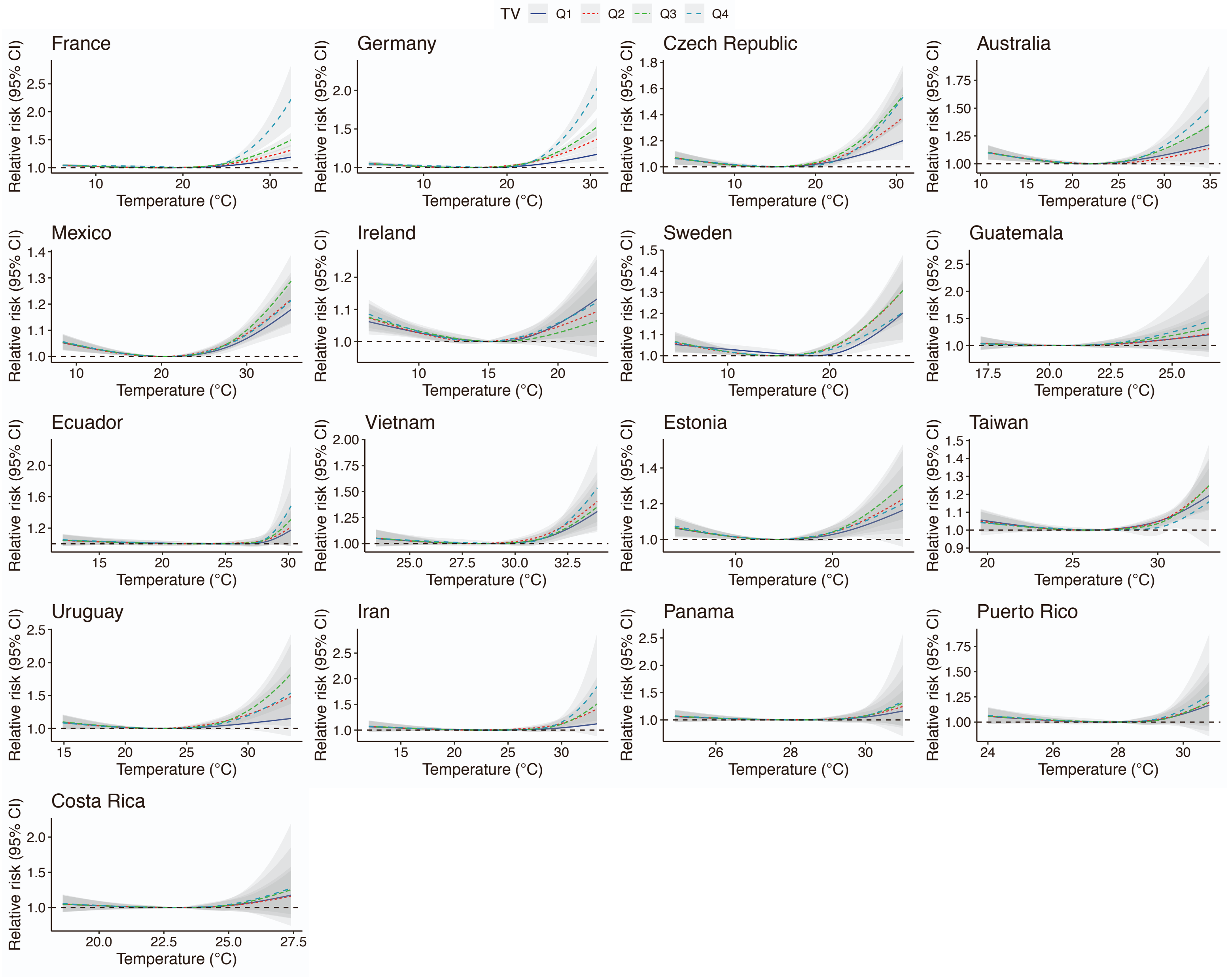

Figure S4. Country-specific exposure-response curve between mean temperature and mortality in warm season, stratified by quantiles of TV 0–2. Definition of abbreviations: TV = temperature variability; Q1 = the first quartile; Q2 = the second quartile; Q3 = the third quartile; Q4 = the fourth quartile.

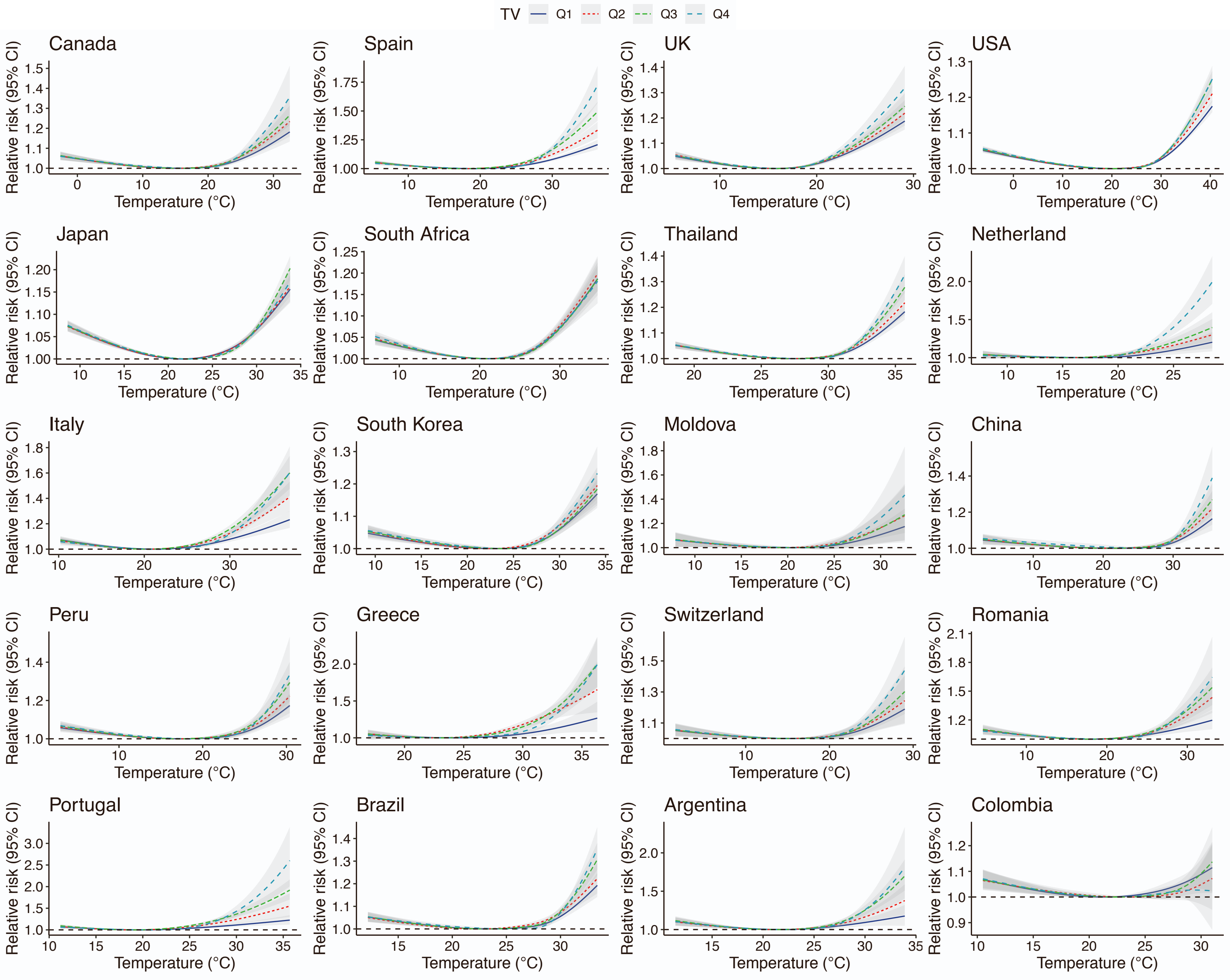
TV Q1 Q2 Q3 Q4
Canada
Spain
UK
USA
Japan
South Africa
Thailand
Netherland
Italy
South Korea
Moldova
China
Peru
Greece
Switzerland
Romania
Portugal
Brazil
Argentina
Colombia
Relative risk (95% CI)
Temperature (°C)

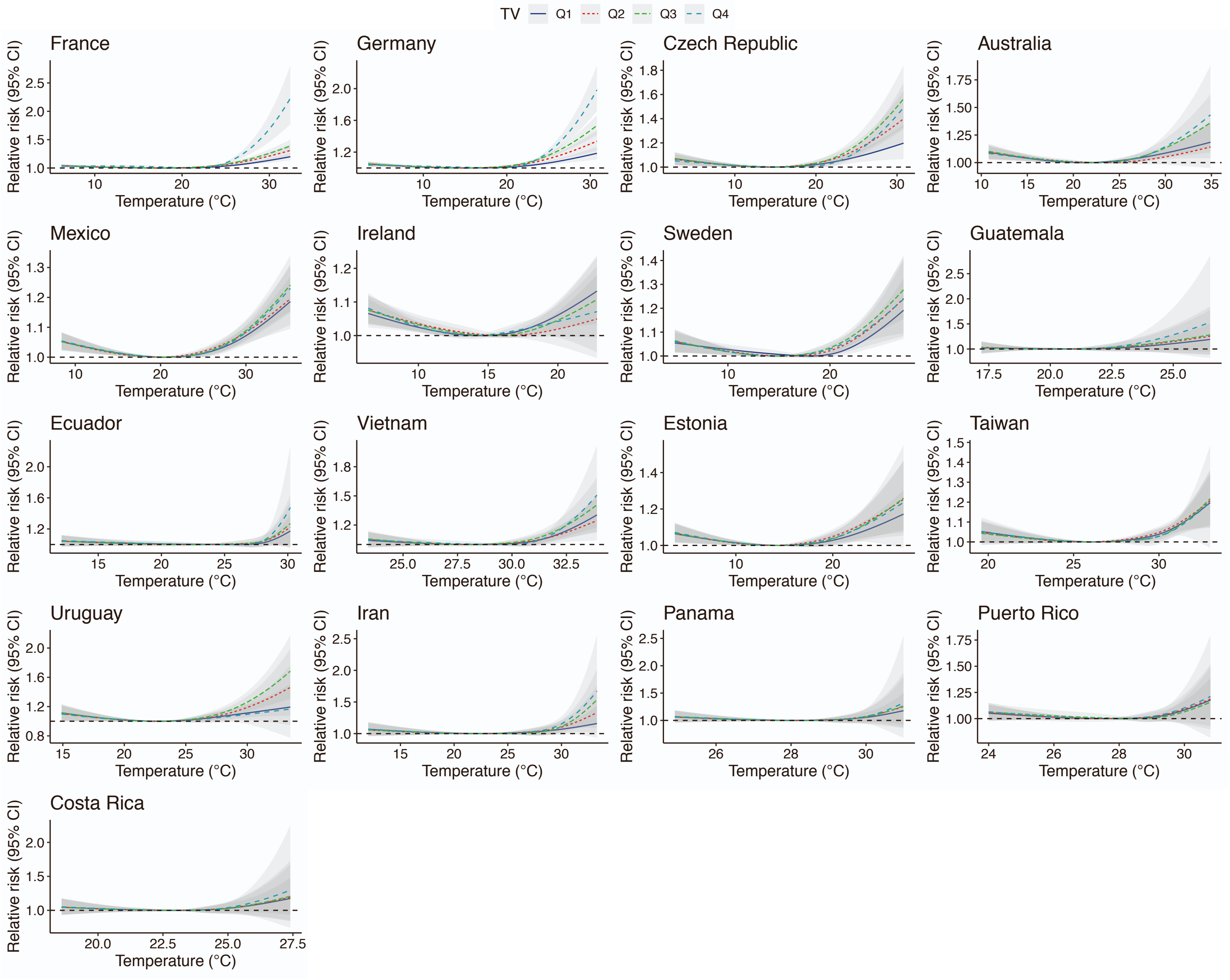

Figure S5. Country-specific exposure-response curve between mean temperature and mortality in warm season, stratified by quantiles of TV 0–3. Definition of abbreviations: TV = temperature variability; Q1 = the first quartile; Q2 = the second quartile; Q3 = the third quartile; Q4 = the fourth quartile.

Table S1. Descriptive information on missing rates of mortality and temperature data at the country level.

Country	Missing rates (%)			
	Minimum temperature	Mean temperature	Maximum temperature	Mortality
Argentina	0.05	0.00	0.00	0.95
Australia	0.00	0.00	0.00	0.31
Brazil	0.59	0.43	0.20	0.01
Canada	0.04	0.05	0.03	1.44
China	0.00	0.00	0.00	0.00
Colombia	2.41	2.09	1.16	0.00
Costa Rica	0.97	0.97	0.97	0.00
Czech Republic	0.00	0.00	0.00	0.00
Ecuador	2.66	2.66	2.66	0.00
Estonia	0.00	0.00	0.00	0.00
France	0.04	0.04	0.04	0.25
Germany	0.00	0.00	0.00	0.00
Greece	7.03	7.08	6.91	0.00
Guatemala	2.15	2.15	2.15	0.00
Iran	0.00	0.00	0.00	0.00
Ireland	0.00	0.00	0.00	0.01
Italy	1.08	0.03	0.19	0.01
Japan	0.01	0.02	0.01	0.00
Mexico	1.61	1.63	1.61	0.00
Moldova	0.00	0.00	0.00	0.00
Netherland	0.00	0.00	0.00	0.00
Panama	5.33	5.33	4.92	0.00
Peru	2.42	2.80	2.33	1.83
Portugal	0.25	0.25	0.25	0.12
Puerto Rico	1.78	2.05	0.65	0.00
Romania	0.00	0.00	0.00	0.00
South Africa	1.85	1.98	1.48	0.00
South Korea	0.00	0.02	0.00	0.00
Spain	0.00	0.00	0.00	0.00
Sweden	0.00	0.00	0.00	0.00
Switzerland	0.00	0.00	0.11	0.00
Thailand	0.07	0.07	0.04	0.00
UK	0.00	0.00	0.00	0.00
Uruguay	0.00	0.00	0.00	0.00
USA	0.00	0.00	0.00	0.00
Vietnam	0.57	0.57	0.57	0.00
Pooled	0.86	0.84	0.73	0.14

Table S2. Correlation coefficients between daily mean temperature and temperature variability.

Country	TV 0–1		TV 0–2		TV 0–3	
	Correlation coefficient	P value	Correlation coefficient	P value	Correlation coefficient	P value
Global	0.10	<0.001	0.10	<0.001	0.10	<0.001
Argentina	0.25	<0.001	0.25	<0.001	0.25	<0.001
Australia	0.27	<0.001	0.27	<0.001	0.27	<0.001
Brazil	0.08	<0.001	0.08	<0.001	0.08	<0.001
Canada	0.08	<0.001	0.08	<0.001	0.08	<0.001
China	-0.29	<0.001	-0.29	<0.001	-0.29	<0.001
Colombia	-0.31	<0.001	-0.31	<0.001	-0.31	<0.001
Costa Rica	0.21	<0.001	0.21	<0.001	0.21	<0.001
Czech Republic	0.52	<0.001	0.52	<0.001	0.52	<0.001
Ecuador	-0.58	<0.001	-0.58	<0.001	-0.58	<0.001
Estonia	0.22	<0.001	0.22	<0.001	0.22	<0.001
France	0.30	<0.001	0.30	<0.001	0.30	<0.001
Germany	0.53	<0.001	0.53	<0.001	0.53	<0.001
Greece	0.42	<0.001	0.42	<0.001	0.42	<0.001
Guatemala	0.35	<0.001	0.35	<0.001	0.35	<0.001
Iran	0.04	0.21	0.04	0.21	0.04	0.21
Ireland	0.05	<0.001	0.05	<0.001	0.05	<0.001
Italy	0.22	<0.001	0.22	<0.001	0.22	<0.001
Japan	0.14	<0.001	0.14	<0.001	0.14	<0.001
Mexico	0.10	<0.001	0.10	<0.001	0.10	<0.001
Moldova	0.32	<0.001	0.32	<0.001	0.32	<0.001
Netherland	0.39	<0.001	0.39	<0.001	0.39	<0.001
Panama	0.07	0.31	0.07	0.31	0.07	0.31
Peru	-0.17	<0.001	-0.17	<0.001	-0.17	<0.001
Portugal	0.58	<0.001	0.58	<0.001	0.58	<0.001
Puerto Rico	0.16	<0.001	0.16	<0.001	0.16	<0.001
Romania	0.27	<0.001	0.27	<0.001	0.27	<0.001
South Africa	0.32	<0.001	0.32	<0.001	0.32	<0.001
South Korea	-0.01	<0.001	-0.01	<0.001	-0.01	<0.001
Spain	0.26	<0.001	0.26	<0.001	0.26	<0.001
Sweden	0.39	<0.001	0.39	<0.001	0.39	<0.001
Switzerland	0.47	<0.001	0.47	<0.001	0.47	<0.001
Thailand	0.38	<0.001	0.38	<0.001	0.38	<0.001
UK	0.07	<0.001	0.07	<0.001	0.07	<0.001
Uruguay	0.54	<0.001	0.54	<0.001	0.54	<0.001
USA	-0.02	<0.001	-0.02	<0.001	-0.02	<0.001
Vietnam	0.51	<0.001	0.51	<0.001	0.51	<0.001

Table S6. Attributable deaths of mortality due to heat exposure, stratified by quantiles of TV in each region.

Region	Attributable deaths (thousand)			
	Q1	Q2	Q3	Q4
North America	19.84 (-17.71 to 55.61)	29.76 (-14.30 to 71.17)	40.21 (-6.13 to 83.16)	48.31 (-2.45 to 93.97)
Central America	0.43 (-0.60 to 1.41)	0.81 (-0.89 to 2.43)	2.69 (-0.65 to 5.82)	4.39 (-0.49 to 8.90)
South America	3.64 (-3.33 to 10.28)	7.15 (-2.94 to 16.52)	10.10 (0.18 to 19.20)	13.65 (0.90 to 24.98)
North Europe	1.08 (-2.11 to 4.19)	2.71 (-3.04 to 8.17)	4.74 (-1.66 to 10.71)	16.19 (3.35 to 27.65)
Central Europe	1.35 (-1.51 to 4.12)	5.57 (-1.14 to 11.84)	12.69 (2.89 to 21.82)	42.31 (28.92 to 54.50)
South Europe	4.79 (0.29 to 9.01)	12.07 (4.06 to 19.45)	16.96 (7.30 to 25.84)	31.38 (17.81 to 43.62)
South Africa	2.25 (-4.30 to 8.52)	4.31 (-5.12 to 13.15)	6.49 (-5.66 to 17.66)	9.77 (-8.72 to 25.81)
Middle-East Asia	0.16 (-0.17 to 0.46)	0.37 (-0.04 to 0.74)	0.44 (0.04 to 0.81)	0.40 (0.03 to 0.73)
East Asia	28.43 (1.04 to 54.09)	56.67 (13.58 to 97.00)	82.96 (32.71 to 129.98)	77.09 (31.52 to 119.04)
South-East Asia	0.81 (-1.23 to 2.85)	1.76 (-2.54 to 5.87)	3.38 (-2.26 to 8.56)	5.51 (-1.34 to 11.40)
Australia	0.22 (-0.35 to 0.75)	0.59 (-0.29 to 1.42)	1.03 (-0.15 to 2.16)	2.31 (0.62 to 3.86)
International	63.01 (-29.99 to 151.28)	121.77 (-12.66 to 247.77)	181.71 (26.61 to 325.73)	251.30 (70.14 to 414.47)

Table S7. Attributable fractions of mortality due to heat exposure stratified by quantiles of TV in each country.

Country	Q1 of TV	Q2 of TV	Q3 of TV	Q4 of TV
Argentina	1.05 (-0.48 to 2.50)	2.94 (0.69 to 5.06)	5.32 (3.05 to 7.45)	4.41 (2.14 to 6.49)
Australia	0.25 (-0.41 to 0.86)	0.69 (-0.33 to 1.64)	1.19 (-0.18 to 2.49)	2.62 (0.70 to 4.39)
Brazil	0.85 (-0.69 to 2.32)	1.61 (-0.72 to 3.78)	2.07 (-0.11 to 4.06)	3.38 (0.34 to 6.08)
Canada	0.65 (-0.48 to 1.72)	1.24 (-0.23 to 2.61)	1.62 (-0.10 to 3.21)	2.08 (-0.10 to 4.02)
China	1.27 (-0.66 to 3.10)	1.80 (-1.20 to 4.61)	3.03 (-0.35 to 6.11)	3.57 (0.08 to 6.44)
Colombia	0.51 (-0.81 to 1.76)	0.51 (-0.62 to 1.56)	0.72 (-0.79 to 2.13)	1.39 (-1.17 to 3.63)
Costa Rica	0.59 (-1.37 to 2.55)	0.38 (-1.29 to 1.89)	0.92 (-1.58 to 3.29)	1.32 (-1.83 to 4.23)
Czech Republic	0.34 (-0.37 to 0.99)	1.45 (-0.09 to 2.87)	3.63 (1.11 to 5.92)	5.24 (2.26 to 7.99)
Ecuador	0.26 (-0.77 to 1.24)	1.02 (-1.96 to 3.74)	1.30 (-1.49 to 3.87)	1.67 (-0.74 to 3.93)
Estonia	0.35 (-0.81 to 1.51)	0.92 (-0.83 to 2.59)	2.28 (-0.42 to 4.78)	2.59 (-1.61 to 6.35)
France	0.23 (-0.29 to 0.74)	0.63 (-0.48 to 1.68)	1.63 (0.08 to 3.09)	9.49 (7.39 to 11.36)
Germany	0.13 (-0.27 to 0.52)	0.59 (-0.45 to 1.57)	2.20 (0.58 to 3.71)	8.02 (5.74 to 10.12)
Greece	1.65 (0.09 to 3.10)	6.23 (3.89 to 8.33)	7.80 (4.98 to 10.25)	9.27 (5.28 to 12.73)
Guatemala	0.29 (-0.36 to 0.91)	0.08 (-0.24 to 0.35)	0.46 (-0.95 to 1.81)	0.59 (-1.30 to 2.40)
Iran	1.65 (-1.76 to 4.77)	3.64 (-0.38 to 7.33)	4.23 (0.37 to 7.75)	3.73 (0.26 to 6.89)
Ireland	0.22 (-0.48 to 0.88)	0.51 (-0.47 to 1.41)	0.16 (-0.28 to 0.59)	1.25 (-0.57 to 2.82)
Italy	2.21 (0.18 to 4.15)	4.22 (1.35 to 6.92)	4.37 (1.50 to 7.02)	5.15 (2.22 to 7.79)
Japan	0.90 (0.10 to 1.64)	1.76 (0.52 to 2.92)	2.55 (1.12 to 3.89)	2.37 (1.05 to 3.59)
Mexico	0.22 (-0.29 to 0.71)	0.42 (-0.45 to 1.25)	1.39 (-0.29 to 2.96)	2.23 (-0.20 to 4.48)
Moldova	0.08 (-0.38 to 0.56)	0.35 (-0.97 to 1.67)	1.15 (-0.79 to 3.02)	4.12 (0.72 to 7.17)
Netherland	0.10 (-0.47 to 0.66)	0.46 (-0.83 to 1.69)	1.55 (-0.33 to 3.31)	6.35 (2.83 to 9.44)
Panama	0.66 (-1.59 to 2.94)	3.77 (-4.10 to 10.02)	5.15 (-5.97 to 13.42)	2.04 (-1.80 to 5.23)
Peru	1.12 (-1.47 to 3.56)	1.83 (-1.70 to 5.02)	1.66 (-1.02 to 4.08)	1.26 (-0.87 to 3.07)
Portugal	0.21 (-0.15 to 0.56)	1.29 (0.48 to 2.06)	2.73 (1.48 to 3.87)	9.12 (6.80 to 11.19)
Puerto Rico	0.35 (-2.01 to 2.76)	0.37 (-1.14 to 1.74)	0.54 (-1.54 to 2.45)	2.03 (-1.48 to 5.17)
Romania	0.70 (-0.25 to 1.63)	3.16 (1.11 to 5.01)	3.46 (1.23 to 5.56)	5.96 (3.58 to 8.17)
South Africa	0.44 (-0.85 to 1.68)	0.82 (-0.98 to 2.51)	1.22 (-1.06 to 3.32)	1.79 (-1.60 to 4.72)
South Korea	0.44 (-0.63 to 1.49)	1.01 (-0.46 to 2.40)	1.33 (-0.46 to 2.99)	0.97 (-0.18 to 2.00)
Spain	1.44 (0.17 to 2.61)	3.23 (0.91 to 5.35)	4.46 (1.70 to 7.01)	6.69 (3.00 to 10.05)
Sweden	0.06 (-0.17 to 0.27)	0.43 (-0.43 to 1.27)	1.29 (-0.16 to 2.61)	3.35 (0.75 to 5.74)
Switzerland	0.33 (-0.57 to 1.23)	1.18 (-1.08 to 3.30)	2.62 (-0.83 to 5.83)	5.64 (0.72 to 9.92)
Thailand	0.39 (-0.89 to 1.67)	0.86 (-1.93 to 3.52)	2.11 (-1.62 to 5.51)	3.59 (-0.95 to 7.50)
UK	0.18 (-0.31 to 0.66)	0.41 (-0.47 to 1.25)	0.70 (-0.27 to 1.61)	2.33 (0.55 to 3.93)
Uruguay	0.50 (-0.13 to 1.10)	1.91 (-0.11 to 3.84)	4.17 (1.02 to 7.10)	4.94 (1.68 to 7.94)
USA	0.72 (-0.65 to 2.03)	1.05 (-0.55 to 2.56)	1.43 (-0.23 to 2.98)	1.70 (-0.09 to 3.32)
Vietnam	2.87 (0.11 to 5.60)	5.63 (2.03 to 9.04)	3.90 (0.52 to 7.27)	2.99 (0.48 to 5.21)
Total	0.70 (-0.33 to 1.69)	1.34 (-0.14 to 2.73)	1.99 (0.29 to 3.57)	2.73 (0.76 to 4.50)

Definition of abbreviations: TV = temperature variability; Q1 = the first quartile; Q2 = the second quartile; Q3 = the third quartile; Q4 = the fourth quartile.

Table S8. Attributable fractions of mortality due to heat exposure stratified by TVST for each quartile of TV in each country.

Country	Attributable fractions (%): MMT–TVST				Attributable fractions (%): above TVST			
	Q1 of TV	Q2 of TV	Q3 of TV	Q4 of TV	Q1 of TV	Q2 of TV	Q3 of TV	Q4 of TV
Argentina	1.02 (-0.49 to 2.45)	2.62 (0.42 to 4.69)	3.60 (1.48 to 5.57)	2.71 (0.66 to 4.67)	8.90 (-1.13 to 18.06)	18.01 (6.98 to 27.26)	29.16 (21.63 to 36.17)	22.33 (13.29 to 29.75)
Australia	0.25 (-0.40 to 0.85)	0.68 (-0.33 to 1.63)	1.16 (-0.19 to 2.45)	2.01 (0.33 to 3.58)	5.75 (-10.36 to 16.87)	8.90 (-3.30 to 18.97)	15.69 (0.26 to 27.67)	26.46 (4.71 to 41.51)
Brazil	0.82 (-0.71 to 2.28)	1.57 (-0.74 to 3.71)	1.95 (-0.19 to 3.92)	3.04 (0.08 to 5.71)	15.44 (-7.66 to 32.03)	12.65 (-6.28 to 26.59)	20.64 (4.99 to 31.93)	22.48 (3.06 to 35.91)
Canada	0.62 (-0.50 to 1.67)	1.09 (-0.32 to 2.42)	1.31 (-0.28 to 2.80)	1.52 (-0.33 to 3.23)	10.74 (-1.00 to 20.62)	13.68 (2.70 to 22.85)	13.55 (2.38 to 23.21)	12.98 (-2.13 to 24.18)
China	1.03 (-0.72 to 2.74)	1.63 (-1.20 to 4.35)	2.58 (-0.58 to 5.50)	2.79 (0.02 to 5.21)	13.52 (-4.98 to 27.38)	14.75 (-6.49 to 30.05)	19.49 (2.67 to 32.44)	22.43 (-1.30 to 38.39)
Colombia	0.50 (-0.81 to 1.74)	0.46 (-0.60 to 1.45)	0.62 (-0.80 to 1.94)	1.07 (-0.90 to 2.86)	8.98 (-12.42 to 27.19)	3.05 (-4.02 to 8.62)	4.89 (-4.04 to 12.77)	7.44 (-10.84 to 21.30)
Costa Rica	0.40 (-1.31 to 2.12)	0.21 (-1.04 to 1.47)	0.57 (-1.54 to 2.58)	0.97 (-2.04 to 3.83)	6.20 (-5.22 to 16.07)	4.92 (-8.97 to 16.37)	9.77 (-8.96 to 23.57)	7.61 (-5.25 to 18.13)
Czech Republic	0.34 (-0.37 to 0.99)	1.45 (-0.09 to 2.87)	3.63 (1.10 to 5.92)	4.91 (1.93 to 7.64)	/	/	9.22 (0.47 to 15.03)	26.37 (11.93 to 37.18)
Ecuador	0.15 (-0.68 to 1.00)	0.56 (-1.81 to 2.91)	1.15 (-1.44 to 3.60)	0.84 (-0.79 to 2.39)	7.59 (-12.45 to 21.34)	11.58 (-9.17 to 27.37)	7.42 (-3.31 to 16.62)	13.81 (-6.61 to 29.31)
Estonia	0.34 (-0.82 to 1.50)	0.82 (-0.86 to 2.43)	1.62 (-0.69 to 3.80)	1.96 (-1.24 to 4.91)	5.76 (-4.74 to 14.67)	14.86 (-4.45 to 29.80)	16.45 (-0.85 to 29.15)	8.01 (-14.39 to 23.75)
France	0.18 (-0.28 to 0.61)	0.35 (-0.53 to 1.19)	0.59 (-0.44 to 1.55)	0.63 (-0.34 to 1.53)	4.68 (-2.20 to 11.27)	7.12 (-0.22 to 13.82)	9.01 (2.46 to 15.02)	24.23 (19.51 to 28.25)
Germany	0.13 (-0.27 to 0.51)	0.54 (-0.46 to 1.50)	1.41 (-0.06 to 2.78)	2.13 (0.50 to 3.65)	4.73 (-2.76 to 11.63)	8.39 (0.66 to 15.39)	12.90 (7.70 to 17.84)	21.53 (16.59 to 26.06)
Greece	1.65 (0.09 to 3.10)	6.23 (3.89 to 8.33)	7.02 (4.28 to 9.42)	7.21 (3.15 to 10.78)	/	/	31.07 (22.59 to 38.92)	44.00 (34.40 to 51.90)
Guatemala	0.11 (-0.36 to 0.59)	0.01 (-0.11 to 0.12)	0.17 (-0.75 to 1.05)	0.42 (-1.36 to 2.11)	10.61 (-6.22 to 23.30)	3.29 (-7.99 to 12.63)	4.72 (-5.49 to 13.17)	4.38 (-2.08 to 10.54)
Iran	1.53 (-1.89 to 4.60)	3.15 (-0.71 to 6.76)	3.16 (-0.54 to 6.53)	2.53 (-0.65 to 5.47)	9.51 (-5.94 to 21.41)	16.83 (5.74 to 26.01)	20.86 (6.90 to 31.11)	23.47 (1.68 to 38.44)
Ireland	0.18 (-0.44 to 0.76)	0.42 (-0.43 to 1.22)	0.11 (-0.22 to 0.42)	0.64 (-0.60 to 1.78)	4.10 (-6.82 to 13.54)	5.44 (-5.57 to 14.42)	2.36 (-4.12 to 8.49)	7.81 (-4.69 to 17.72)
Italy	2.10 (0.10 to 4.00)	3.93 (1.11 to 6.57)	4.10 (1.27 to 6.70)	4.23 (1.52 to 6.72)	15.95 (4.13 to 25.72)	22.61 (10.80 to 31.84)	23.41 (11.05 to 33.37)	27.67 (9.82 to 39.98)
Japan	0.90 (0.10 to 1.64)	1.76 (0.52 to 2.92)	2.55 (1.12 to 3.89)	2.36 (1.04 to 3.58)	/	/	17.86 (7.48 to 26.89)	20.36 (7.04 to 31.97)
Mexico	0.22 (-0.29 to 0.70)	0.38 (-0.46 to 1.18)	1.14 (-0.47 to 2.65)	1.40 (-0.75 to 3.41)	27.01 (-111.76 to 130.51)	15.21 (-3.89 to 30.50)	12.62 (2.67 to 21.20)	9.45 (0.62 to 17.02)
Moldova	0.08 (-0.38 to 0.56)	0.35 (-0.97 to 1.67)	1.15 (-0.79 to 3.02)	3.98 (0.68 to 6.99)	/	/	/	63.11 (6.61 to 97.53)
Netherland	0.08 (-0.47 to 0.63)	0.41 (-0.85 to 1.60)	1.11 (-0.59 to 2.69)	2.23 (-0.38 to 4.63)	6.21 (-4.26 to 15.12)	10.23 (-2.13 to 20.22)	13.93 (4.24 to 22.67)	22.33 (11.18 to 31.24)
Panama	0.66 (-1.59 to 2.94)	2.87 (-3.54 to 8.30)	3.77 (-4.46 to 10.58)	1.38 (-1.38 to 3.96)	/	16.25 (-18.03 to 37.83)	18.49 (-18.27 to 41.41)	46.91 (-73.10 to 107.32)
Peru	0.55 (-1.40 to 2.50)	0.99 (-1.62 to 3.53)	0.79 (-1.04 to 2.53)	0.47 (-0.75 to 1.60)	8.57 (-5.98 to 20.49)	10.41 (-4.78 to 22.63)	12.16 (-4.62 to 25.08)	12.48 (-9.48 to 27.97)
Portugal	0.20 (-0.15 to 0.55)	1.08 (0.31 to 1.80)	1.75 (0.61 to 2.78)	2.73 (0.74 to 4.60)	11.31 (4.67 to 16.82)	22.30 (15.43 to 28.23)	20.83 (15.85 to 25.35)	25.15 (20.86 to 28.96)
Puerto Rico	0.33 (-2.04 to 2.70)	0.28 (-1.06 to 1.52)	0.42 (-1.41 to 2.18)	1.14 (-1.53 to 3.57)	7.19 (-13.54 to 24.88)	10.57 (-18.97 to 34.38)	8.55 (-14.84 to 26.35)	12.45 (-10.61 to 28.21)
Romania	0.66 (-0.28 to 1.59)	3.09 (1.06 to 4.93)	3.19 (0.98 to 5.29)	4.09 (2.00 to 6.04)	14.11 (3.66 to 22.78)	21.91 (12.26 to 30.27)	24.27 (13.33 to 32.96)	29.87 (18.76 to 38.28)
South Africa	0.39 (-0.86 to 1.59)	0.70 (-0.96 to 2.29)	0.88 (-1.07 to 2.70)	1.22 (-1.45 to 3.65)	7.87 (-5.73 to 18.89)	8.39 (-5.03 to 18.92)	9.29 (-5.85 to 21.12)	7.74 (-9.82 to 20.51)
South Korea	0.44 (-0.63 to 1.48)	0.90 (-0.51 to 2.25)	1.09 (-0.51 to 2.59)	0.65 (-0.24 to 1.48)	12.24 (-5.05 to 25.66)	17.49 (3.94 to 28.40)	12.41 (-0.96 to 23.67)	15.25 (-3.66 to 29.06)

Spain	1.31 (0.08 to 2.44)	2.83 (0.61 to 4.87)	3.32 (0.71 to 5.74)	3.51 (0.28 to 6.54)	13.21 (6.23 to 19.49)	16.55 (8.95 to 23.22)	19.43 (11.54 to 26.16)	21.88 (12.44 to 29.48)
Sweden	0.06 (-0.17 to 0.27)	0.40 (-0.45 to 1.21)	0.96 (-0.38 to 2.20)	2.01 (-0.04 to 3.91)	/	8.55 (-0.86 to 16.47)	15.18 (5.41 to 24.18)	13.40 (2.70 to 22.18)
Switzerland	0.33 (-0.58 to 1.22)	1.15 (-1.05 to 3.26)	2.52 (-0.82 to 5.64)	3.52 (-0.16 to 6.95)	18.41 (-1.21 to 34.02)	8.99 (-9.74 to 22.78)	14.78 (-9.31 to 32.06)	23.87 (2.12 to 39.17)
Thailand	0.34 (-0.91 to 1.58)	0.80 (-1.91 to 3.40)	1.64 (-1.71 to 4.76)	2.21 (-1.15 to 5.31)	10.50 (-1.73 to 21.51)	10.08 (-11.76 to 26.41)	14.15 (-5.02 to 28.75)	16.27 (-6.40 to 32.18)
UK	0.16 (-0.31 to 0.63)	0.36 (-0.47 to 1.15)	0.52 (-0.32 to 1.33)	1.09 (-0.10 to 2.21)	5.33 (-2.52 to 12.14)	7.23 (-2.39 to 15.33)	9.51 (-0.48 to 17.70)	13.60 (3.08 to 21.60)
Uruguay	0.50 (-0.13 to 1.10)	1.80 (-0.19 to 3.70)	2.87 (-0.00 to 5.53)	2.72 (0.32 to 4.98)	/	9.33 (2.08 to 15.26)	22.12 (9.08 to 32.29)	27.77 (6.81 to 42.10)
USA	0.63 (-0.70 to 1.90)	0.81 (-0.64 to 2.18)	0.97 (-0.47 to 2.31)	1.03 (-0.42 to 2.40)	8.14 (-1.11 to 16.24)	9.78 (-0.03 to 18.15)	12.17 (2.11 to 20.90)	11.55 (0.31 to 20.55)
Vietnam	2.45 (-0.33 to 5.13)	4.18 (0.71 to 7.47)	2.72 (-0.58 to 5.88)	2.12 (-0.10 to 4.14)	21.54 (11.65 to 29.37)	24.83 (11.41 to 34.96)	21.73 (10.12 to 30.66)	27.78 (11.68 to 41.01)
Total	0.66 (-0.35 to 1.62)	1.23 (-0.18 to 2.55)	1.71 (0.14 to 3.17)	1.83 (0.17 to 3.37)	8.61 (-1.82 to 17.49)	10.48 (-0.02 to 19.25)	12.90 (2.70 to 21.53)	16.36 (5.81 to 24.62)

Definition of abbreviations: TV = temperature variability; Q1 = the first quartile; Q2 = the second quartile; Q3 = the third quartile; Q4 = the fourth quartile; MMT = minimum mortality temperature; TVST = temperature variability sensitive heat threshold.

Table S9. Attributable fractions of mortality due to heat exposure stratified by quantiles of TV 0–2 in each country.

Country	Attributable fractions (%)			
	Q1 of TV 0–2	Q2 of TV 0–2	Q3 of TV 0–2	Q4 of TV 0–2
Argentina	1.22 (-0.50 to 2.85)	2.95 (0.82 to 4.96)	4.30 (2.21 to 6.24)	4.11 (1.82 to 6.23)
Australia	0.64 (-0.26 to 1.53)	0.41 (-0.42 to 1.21)	1.33 (-0.22 to 2.79)	2.66 (0.74 to 4.41)
Brazil	0.71 (-0.69 to 2.03)	1.63 (-0.75 to 3.85)	2.22 (-0.31 to 4.57)	3.00 (0.19 to 5.52)
Canada	0.58 (-0.54 to 1.65)	1.47 (-0.05 to 2.92)	1.31 (-0.30 to 2.77)	2.17 (0.29 to 3.82)
China	1.17 (-0.85 to 3.09)	1.79 (-1.08 to 4.52)	2.53 (-0.47 to 5.32)	3.37 (-0.00 to 6.21)
Colombia	0.78 (-0.49 to 1.98)	0.61 (-0.58 to 1.73)	0.56 (-0.87 to 1.88)	1.35 (-1.23 to 3.58)
Costa Rica	0.43 (-1.19 to 1.96)	0.42 (-1.37 to 2.26)	0.74 (-1.60 to 2.91)	1.10 (-1.89 to 3.89)
Czech Republic	0.29 (-0.32 to 0.87)	1.37 (-0.16 to 2.83)	3.75 (1.17 to 6.13)	4.73 (2.21 to 7.04)
Ecuador	0.29 (-0.33 to 0.89)	1.16 (-1.70 to 3.94)	1.39 (-1.33 to 3.92)	1.62 (-0.69 to 3.78)
Estonia	0.18 (-0.70 to 1.03)	0.88 (-0.88 to 2.58)	1.70 (-0.99 to 4.20)	2.49 (-1.95 to 6.46)
France	0.26 (-1.82 to 2.08)	0.63 (-0.38 to 1.59)	1.59 (-0.21 to 3.27)	9.36 (7.26 to 11.23)
Germany	0.09 (-0.24 to 0.42)	0.76 (-0.30 to 1.75)	2.03 (0.37 to 3.57)	7.69 (5.48 to 9.70)
Greece	0.88 (-0.39 to 2.08)	5.54 (3.30 to 7.55)	7.78 (4.82 to 10.43)	10.57 (6.59 to 13.99)
Guatemala	0.24 (-0.28 to 0.77)	0.17 (-0.44 to 0.77)	0.65 (-1.27 to 2.51)	0.53 (-1.52 to 2.45)
Iran	1.00 (-2.18 to 3.97)	3.54 (0.07 to 6.81)	3.34 (-0.40 to 6.84)	4.71 (1.23 to 7.92)
Ireland	0.20 (-0.28 to 0.66)	0.41 (-0.43 to 1.20)	0.20 (-0.52 to 0.88)	1.29 (-0.69 to 3.06)
Italy	1.93 (0.00 to 3.72)	3.96 (1.09 to 6.63)	4.57 (1.60 to 7.28)	4.91 (1.89 to 7.61)
Japan	0.97 (0.08 to 1.80)	1.85 (0.59 to 3.03)	2.22 (0.88 to 3.46)	2.38 (1.09 to 3.57)
Mexico	0.07 (-0.26 to 0.38)	0.42 (-0.58 to 1.38)	1.19 (-0.38 to 2.65)	2.72 (-0.07 to 5.31)
Moldova	0.08 (-0.28 to 0.43)	0.52 (-1.07 to 2.12)	0.96 (-1.11 to 2.89)	3.78 (0.58 to 6.59)
Netherland	0.07 (-0.33 to 0.47)	0.39 (-0.68 to 1.42)	1.69 (-0.42 to 3.66)	6.65 (3.57 to 9.45)
Panama	1.01 (-1.81 to 3.65)	4.23 (-2.74 to 9.75)	5.33 (-5.53 to 13.42)	1.57 (-1.57 to 4.50)
Peru	1.14 (-1.25 to 3.36)	1.70 (-1.44 to 4.64)	1.72 (-1.35 to 4.52)	1.27 (-0.89 to 3.05)
Portugal	0.14 (-0.18 to 0.45)	0.81 (0.03 to 1.53)	2.76 (1.35 to 4.04)	9.35 (6.94 to 11.48)
Puerto Rico	0.60 (-2.22 to 3.24)	0.82 (-1.81 to 3.36)	0.55 (-1.43 to 2.34)	1.90 (-1.82 to 5.22)
Romania	0.74 (-0.28 to 1.69)	2.14 (0.31 to 3.82)	4.08 (1.77 to 6.24)	5.96 (3.62 to 8.15)
South Africa	0.44 (-0.83 to 1.65)	0.73 (-0.98 to 2.35)	1.16 (-1.08 to 3.24)	1.86 (-1.10 to 4.41)
South Korea	0.50 (-0.61 to 1.57)	1.12 (-0.54 to 2.71)	1.09 (-0.55 to 2.59)	0.87 (-0.24 to 1.82)
Spain	1.18 (-0.08 to 2.33)	2.97 (0.74 to 5.02)	4.80 (1.69 to 7.62)	6.91 (3.26 to 10.12)
Sweden	0.04 (-0.11 to 0.19)	0.60 (-0.58 to 1.71)	1.33 (-0.09 to 2.64)	2.83 (0.12 to 5.28)
Switzerland	0.21 (-0.56 to 0.94)	1.21 (-1.09 to 3.43)	2.07 (-1.30 to 5.23)	5.85 (0.69 to 10.34)
Thailand	0.34 (-0.89 to 1.51)	0.94 (-1.70 to 3.50)	2.27 (-1.63 to 5.84)	3.57 (-1.18 to 7.67)
UK	0.11 (-0.29 to 0.49)	0.28 (-0.43 to 0.98)	0.51 (-0.35 to 1.33)	2.69 (0.55 to 4.61)
Uruguay	0.28 (-0.41 to 0.92)	2.60 (0.43 to 4.64)	4.77 (1.61 to 7.75)	5.31 (1.77 to 8.56)
USA	0.67 (-0.64 to 1.89)	1.08 (-0.54 to 2.61)	1.28 (-0.34 to 2.78)	1.82 (-0.07 to 3.52)
Vietnam	2.39 (-0.28 to 4.83)	4.98 (1.27 to 8.46)	3.03 (-0.56 to 6.60)	2.51 (-0.17 to 4.92)
Total	0.69 (-0.36 to 1.68)	1.36 (-0.12 to 2.76)	1.81 (0.14 to 3.36)	2.78 (0.81 to 4.55)

Definition of abbreviations: TV = temperature variability; Q1 = the first quartile; Q2 = the second quartile; Q3 = the third quartile; Q4 = the fourth quartile.

Table S10. Attributable fractions of mortality due to heat exposure stratified by quantiles of TV 0–3 in each country.

Country	Attributable fractions (%)			
	Q1 of TV 0–3	Q2 of TV 0–3	Q3 of TV 0–3	Q4 of TV 0–3
Argentina	1.54 (-0.18 to 3.19)	2.39 (0.24 to 4.39)	3.89 (1.77 to 5.85)	4.12 (1.68 to 6.40)
Australia	0.76 (-0.27 to 1.74)	0.52 (-0.38 to 1.35)	1.31 (-0.15 to 2.70)	2.06 (0.23 to 3.74)
Brazil	0.82 (-0.69 to 2.24)	1.70 (-0.78 to 3.97)	2.09 (-0.55 to 4.51)	3.03 (0.38 to 5.41)
Canada	0.71 (-0.46 to 1.81)	1.19 (-0.37 to 2.63)	1.62 (-0.15 to 3.25)	1.74 (0.08 to 3.18)
China	1.09 (-0.82 to 2.97)	1.88 (-1.16 to 4.70)	2.38 (-0.89 to 5.34)	3.35 (-0.02 to 6.10)
Colombia	0.73 (-0.68 to 2.05)	0.44 (-0.84 to 1.59)	1.03 (-1.26 to 3.18)	1.00 (-2.06 to 3.76)
Costa Rica	0.46 (-1.21 to 2.11)	0.61 (-1.73 to 2.82)	0.66 (-1.81 to 2.95)	0.97 (-1.68 to 3.43)
Czech Republic	0.35 (-0.39 to 1.05)	1.93 (0.14 to 3.56)	3.71 (1.23 to 6.00)	4.25 (1.84 to 6.47)
Ecuador	0.20 (-0.41 to 0.80)	1.31 (-1.56 to 3.98)	1.71 (-1.72 to 4.89)	1.41 (-0.62 to 3.35)
Estonia	0.18 (-0.67 to 0.99)	1.09 (-0.80 to 2.83)	1.47 (-1.28 to 4.04)	2.85 (-1.68 to 6.88)
France	0.37 (-1.71 to 2.17)	0.74 (-0.45 to 1.81)	1.60 (-0.17 to 3.23)	8.83 (6.68 to 10.72)
Germany	0.12 (-0.27 to 0.48)	0.75 (-0.39 to 1.78)	2.42 (0.61 to 4.08)	7.44 (5.13 to 9.54)
Greece	1.00 (-0.25 to 2.19)	6.69 (4.11 to 8.83)	8.70 (5.67 to 11.43)	11.28 (7.16 to 14.85)
Guatemala	0.08 (-0.13 to 0.29)	0.36 (-0.83 to 1.48)	0.73 (-1.63 to 2.95)	0.38 (-0.78 to 1.45)
Iran	2.03 (-1.69 to 5.42)	2.73 (-0.94 to 6.07)	3.05 (-0.52 to 6.36)	5.25 (1.33 to 8.81)
Ireland	0.24 (-0.45 to 0.91)	0.18 (-0.33 to 0.66)	0.41 (-0.57 to 1.33)	1.05 (-1.05 to 2.99)
Italy	1.85 (-0.03 to 3.62)	4.46 (1.41 to 7.21)	4.87 (1.77 to 7.71)	4.43 (1.41 to 7.12)
Japan	1.09 (0.15 to 1.98)	1.83 (0.52 to 3.03)	2.13 (0.82 to 3.37)	2.15 (0.93 to 3.29)
Mexico	0.11 (-0.29 to 0.48)	0.47 (-0.70 to 1.55)	0.82 (-0.41 to 1.99)	2.76 (0.22 to 5.12)
Moldova	0.06 (-0.27 to 0.40)	0.85 (-1.21 to 2.78)	0.89 (-1.17 to 2.83)	3.53 (0.47 to 6.08)
Netherland	0.07 (-0.28 to 0.42)	0.74 (-0.79 to 2.12)	1.50 (-0.64 to 3.50)	6.70 (3.80 to 9.35)
Panama	0.75 (-1.46 to 2.91)	4.71 (-3.57 to 11.17)	5.00 (-5.83 to 13.52)	1.39 (-1.62 to 4.28)
Peru	1.21 (-1.30 to 3.61)	1.52 (-1.55 to 4.36)	1.92 (-1.38 to 4.92)	1.05 (-1.08 to 2.75)
Portugal	0.15 (-0.20 to 0.48)	0.98 (0.04 to 1.83)	3.04 (1.48 to 4.44)	8.85 (6.66 to 10.81)
Puerto Rico	0.57 (-1.57 to 2.63)	0.66 (-1.92 to 3.08)	0.51 (-1.36 to 2.08)	1.82 (-2.18 to 5.36)
Romania	0.83 (-0.24 to 1.85)	1.89 (0.14 to 3.54)	4.07 (1.75 to 6.25)	5.96 (3.58 to 8.18)
South Africa	0.41 (-0.77 to 1.54)	0.76 (-0.98 to 2.35)	1.14 (-1.58 to 3.61)	1.85 (-1.45 to 4.67)
South Korea	0.55 (-0.58 to 1.65)	1.16 (-0.72 to 2.87)	1.03 (-0.50 to 2.40)	0.74 (-0.25 to 1.58)
Spain	1.25 (-0.00 to 2.40)	3.12 (0.66 to 5.35)	4.99 (1.87 to 7.83)	6.85 (2.99 to 10.22)
Sweden	0.03 (-0.07 to 0.14)	0.41 (-0.60 to 1.36)	1.37 (-0.45 to 3.05)	3.41 (0.79 to 5.77)
Switzerland	0.19 (-0.55 to 0.89)	0.91 (-1.38 to 3.04)	1.93 (-1.57 to 5.19)	6.08 (0.78 to 10.75)
Thailand	0.35 (-1.03 to 1.66)	0.85 (-1.87 to 3.36)	2.44 (-1.69 to 6.23)	3.38 (-1.34 to 7.39)
UK	0.11 (-0.26 to 0.46)	0.23 (-0.43 to 0.85)	0.53 (-0.41 to 1.43)	2.51 (0.45 to 4.34)
Uruguay	0.70 (-0.26 to 1.60)	2.05 (-0.07 to 4.03)	4.45 (1.45 to 7.23)	3.33 (-1.27 to 7.23)
USA	0.72 (-0.64 to 2.01)	1.08 (-0.59 to 2.61)	1.32 (-0.39 to 2.91)	1.71 (-0.13 to 3.36)
Vietnam	2.74 (0.07 to 5.33)	3.60 (-0.30 to 7.22)	4.47 (0.52 to 8.17)	2.12 (-0.68 to 4.62)
Total	0.76 (-0.33 to 1.80)	1.35 (-0.19 to 2.76)	1.82 (0.08 to 3.44)	2.61 (0.67 to 4.34)

Definition of abbreviations: TV = temperature variability; Q1 = the first quartile; Q2 = the second quartile; Q3 = the third quartile; Q4 = the fourth quartile.

Table S11. Attributable fractions of mortality due to heat exposure stratified by TVST for each quartile of TV 0–2 in each country.

Country	Attributable fractions (%): MMT–TVST				Attributable fractions (%): above TVST			
	Q1 of TV 0–2	Q2 of TV 0–2	Q3 of TV 0–2	Q4 of TV 0–2	Q1 of TV 0–2	Q2 of TV 0–2	Q3 of TV 0–2	Q4 of TV 0–2
Argentina	1.19 (-0.52 to 2.82)	2.57 (0.53 to 4.48)	2.90 (0.92 to 4.74)	2.57 (0.41 to 4.58)	10.78 (0.31 to 20.27)	15.25 (4.90 to 23.72)	27.77 (19.57 to 34.71)	19.77 (10.64 to 27.30)
Australia	0.64 (-0.27 to 1.52)	0.41 (-0.42 to 1.21)	1.33 (-0.22 to 2.79)	2.45 (0.62 to 4.09)	13.90 (-11.05 to 30.02)	/	/	34.67 (3.66 to 55.03)
Brazil	0.61 (-0.76 to 1.90)	1.50 (-0.81 to 3.66)	1.84 (-0.59 to 4.09)	2.12 (-0.49 to 4.50)	9.79 (-3.65 to 21.26)	11.86 (-1.82 to 22.96)	14.82 (3.89 to 23.91)	16.36 (2.86 to 26.76)
Canada	0.52 (-0.58 to 1.57)	1.20 (-0.24 to 2.56)	0.99 (-0.39 to 2.27)	1.21 (-0.16 to 2.49)	7.92 (-0.31 to 15.10)	12.55 (3.95 to 19.91)	8.81 (-0.92 to 17.03)	12.27 (0.69 to 21.19)
China	0.97 (-0.97 to 2.79)	1.57 (-1.14 to 4.20)	2.20 (-0.58 to 4.84)	2.50 (-0.20 to 4.91)	10.79 (-6.14 to 23.88)	15.98 (-3.43 to 29.98)	17.72 (-0.34 to 31.46)	23.27 (0.25 to 38.98)
Colombia	0.73 (-0.50 to 1.90)	0.56 (-0.58 to 1.64)	0.45 (-0.82 to 1.62)	0.97 (-0.93 to 2.74)	5.28 (-4.61 to 14.07)	4.10 (-3.27 to 10.25)	3.73 (-5.51 to 11.33)	6.82 (-10.32 to 20.37)
Costa Rica	0.23 (-1.14 to 1.54)	0.27 (-1.28 to 1.86)	0.35 (-1.28 to 1.96)	0.67 (-2.17 to 3.33)	4.53 (-3.55 to 12.29)	3.94 (-6.18 to 12.47)	6.41 (-7.50 to 17.17)	6.39 (-4.59 to 15.96)
Czech Republic	0.29 (-0.32 to 0.87)	1.37 (-0.16 to 2.82)	3.44 (0.90 to 5.78)	3.29 (0.93 to 5.52)	/	13.93 (0.55 to 24.43)	22.50 (13.35 to 31.00)	22.83 (12.43 to 31.24)
Ecuador	0.10 (-0.35 to 0.55)	0.80 (-1.73 to 3.24)	1.03 (-1.37 to 3.26)	0.73 (-1.06 to 2.39)	5.82 (-4.59 to 13.92)	8.13 (-5.35 to 18.91)	8.99 (-2.56 to 18.75)	11.38 (-3.45 to 23.26)
Estonia	0.18 (-0.70 to 1.01)	0.82 (-0.87 to 2.46)	0.99 (-1.11 to 2.97)	1.74 (-1.53 to 4.77)	7.35 (-5.47 to 18.94)	9.48 (-5.01 to 21.25)	12.04 (-4.10 to 24.42)	7.07 (-13.02 to 21.78)
France	0.21 (-1.83 to 2.00)	0.38 (-0.44 to 1.16)	0.67 (-0.60 to 1.85)	0.56 (-0.36 to 1.43)	4.05 (-1.83 to 9.83)	7.24 (0.16 to 13.73)	8.47 (1.86 to 14.53)	23.41 (18.80 to 27.36)
Germany	0.09 (-0.24 to 0.42)	0.69 (-0.33 to 1.64)	1.35 (-0.12 to 2.72)	1.86 (0.33 to 3.29)	4.01 (-3.61 to 11.57)	9.73 (2.04 to 16.66)	11.78 (6.14 to 17.14)	20.90 (16.04 to 25.33)
Greece	0.80 (-0.40 to 1.95)	4.97 (2.84 to 6.91)	5.47 (2.81 to 7.79)	4.56 (1.04 to 7.70)	7.51 (-0.13 to 14.72)	15.28 (10.82 to 19.44)	21.93 (15.80 to 27.55)	25.49 (19.15 to 31.13)
Guatemala	0.06 (-0.33 to 0.42)	0.04 (-0.33 to 0.42)	0.26 (-1.09 to 1.54)	0.38 (-1.50 to 2.17)	7.66 (-4.07 to 17.69)	4.32 (-5.12 to 12.37)	4.47 (-4.55 to 12.74)	3.09 (-2.16 to 7.94)
Iran	0.95 (-2.26 to 3.92)	3.20 (-0.25 to 6.39)	2.75 (-0.98 to 6.18)	2.97 (-0.26 to 5.88)	7.14 (-5.82 to 18.01)	15.50 (4.94 to 23.95)	19.24 (4.97 to 30.03)	32.01 (9.94 to 46.76)
Ireland	0.14 (-0.25 to 0.51)	0.30 (-0.41 to 0.97)	0.11 (-0.39 to 0.58)	0.60 (-0.69 to 1.82)	3.93 (-4.41 to 11.32)	4.52 (-3.36 to 11.69)	2.59 (-6.57 to 10.59)	6.46 (-4.27 to 15.37)
Italy	1.71 (-0.17 to 3.45)	3.38 (0.67 to 5.91)	3.96 (1.15 to 6.53)	3.44 (0.65 to 6.00)	10.82 (2.61 to 17.97)	17.83 (7.78 to 26.07)	20.31 (9.59 to 28.81)	20.24 (7.70 to 29.84)
Japan	0.97 (0.08 to 1.80)	1.85 (0.59 to 3.03)	2.20 (0.87 to 3.45)	2.35 (1.06 to 3.53)	11.82 (-36.66 to 54.47)	12.99 (4.08 to 21.07)	18.90 (9.36 to 27.43)	17.35 (4.73 to 28.67)
Mexico	0.07 (-0.26 to 0.38)	0.38 (-0.60 to 1.31)	0.90 (-0.51 to 2.23)	1.55 (-1.03 to 3.92)	20.29 (-13.73 to 47.23)	14.45 (-5.75 to 30.70)	9.86 (1.44 to 17.25)	9.20 (1.67 to 15.72)
Moldova	0.08 (-0.29 to 0.43)	0.49 (-1.06 to 2.06)	0.66 (-1.05 to 2.35)	1.35 (-0.75 to 3.28)	0.00 (0.00 to 0.00)	3.49 (-2.79 to 8.76)	5.97 (-2.89 to 13.33)	16.05 (2.43 to 26.44)
Netherland	0.05 (-0.34 to 0.42)	0.30 (-0.66 to 1.23)	1.05 (-0.78 to 2.73)	1.20 (-0.58 to 2.92)	4.89 (-3.06 to 12.17)	6.01 (-3.73 to 14.52)	10.62 (2.38 to 18.14)	19.37 (11.08 to 26.39)
Panama	1.01 (-1.81 to 3.65)	2.66 (-2.33 to 6.95)	2.88 (-3.69 to 8.71)	1.57 (-1.57 to 4.50)	/	26.95 (-16.74 to 57.75)	21.96 (-18.63 to 48.09)	/
Peru	0.54 (-1.38 to 2.35)	0.97 (-1.45 to 3.36)	0.87 (-1.33 to 2.95)	0.40 (-0.73 to 1.46)	8.71 (-3.82 to 19.42)	10.15 (-3.87 to 21.67)	11.05 (-4.85 to 23.49)	13.44 (-10.84 to 29.73)
Portugal	0.13 (-0.18 to 0.43)	0.63 (-0.09 to 1.31)	1.64 (0.44 to 2.72)	1.73 (-0.00 to 3.40)	5.61 (-0.05 to 10.97)	15.57 (9.19 to 21.30)	16.21 (11.36 to 20.76)	21.30 (17.24 to 24.97)
Puerto Rico	0.57 (-2.28 to 3.26)	0.52 (-1.63 to 2.72)	0.35 (-1.24 to 1.84)	1.00 (-2.11 to 3.87)	3.65 (-2.46 to 9.00)	8.46 (-8.88 to 22.61)	8.59 (-15.25 to 27.80)	11.67 (-13.28 to 29.23)
Romania	0.70 (-0.32 to 1.65)	2.10 (0.28 to 3.77)	3.83 (1.55 to 5.95)	4.10 (2.07 to 6.02)	13.40 (3.92 to 21.35)	19.53 (7.71 to 29.82)	22.73 (12.90 to 31.21)	29.56 (18.34 to 38.06)
South Africa	0.38 (-0.86 to 1.55)	0.56 (-0.96 to 2.03)	0.74 (-1.09 to 2.45)	1.08 (-1.04 to 3.06)	6.68 (-2.53 to 14.81)	6.56 (-4.26 to 15.41)	7.91 (-4.32 to 17.92)	7.77 (-6.81 to 18.70)
South Korea	0.50 (-0.61 to 1.57)	1.12 (-0.54 to 2.71)	1.09 (-0.54 to 2.59)	0.84 (-0.25 to 1.78)	/	/	13.08 (-14.58 to 31.43)	24.49 (-6.79 to 44.49)

Spain	1.10 (-0.13 to 2.22)	2.56 (0.43 to 4.52)	3.82 (0.90 to 6.48)	3.61 (0.47 to 6.43)	9.67 (3.35 to 15.55)	15.65 (8.65 to 21.92)	18.60 (10.54 to 25.51)	22.49 (13.10 to 29.98)
Sweden	0.04 (-0.11 to 0.18)	0.55 (-0.60 to 1.65)	0.96 (-0.30 to 2.13)	1.42 (-0.48 to 3.20)	4.45 (-2.93 to 11.00)	10.94 (1.06 to 19.33)	12.68 (3.96 to 20.58)	9.88 (0.27 to 18.01)
Switzerland	0.19 (-0.57 to 0.91)	1.13 (-1.11 to 3.28)	1.73 (-1.40 to 4.64)	2.88 (-0.75 to 6.29)	7.55 (-4.03 to 17.23)	11.31 (-3.74 to 23.55)	12.34 (-3.24 to 25.12)	18.99 (2.10 to 31.55)
Thailand	0.31 (-0.90 to 1.45)	0.87 (-1.70 to 3.38)	1.84 (-1.69 to 5.14)	2.23 (-1.40 to 5.57)	7.60 (-4.91 to 17.37)	11.39 (-5.58 to 24.77)	13.91 (-5.17 to 28.29)	15.60 (-7.88 to 32.08)
UK	0.09 (-0.29 to 0.46)	0.23 (-0.42 to 0.87)	0.29 (-0.37 to 0.92)	0.82 (-0.50 to 2.08)	3.55 (-2.20 to 8.82)	4.65 (-2.60 to 11.00)	5.71 (-1.03 to 11.71)	10.68 (2.65 to 17.22)
Uruguay	0.28 (-0.41 to 0.92)	2.25 (0.18 to 4.14)	2.99 (0.00 to 5.79)	2.71 (-0.17 to 5.41)	/	17.75 (7.84 to 26.08)	25.72 (14.24 to 34.55)	21.60 (4.40 to 34.02)
USA	0.49 (-0.72 to 1.63)	0.69 (-0.70 to 2.01)	0.66 (-0.58 to 1.81)	0.78 (-0.66 to 2.14)	6.03 (-0.57 to 11.96)	7.52 (0.13 to 14.15)	8.52 (0.84 to 15.30)	9.51 (0.89 to 16.71)
Vietnam	1.56 (-1.06 to 3.99)	3.18 (-0.21 to 6.41)	1.84 (-1.56 to 5.10)	1.77 (-0.68 to 4.05)	18.02 (8.41 to 25.90)	23.59 (10.32 to 34.02)	18.05 (5.39 to 28.28)	26.53 (6.33 to 41.65)
Total	0.62 (-0.39 to 1.57)	1.20 (-0.18 to 2.50)	1.48 (0.01 to 2.85)	1.68 (0.08 to 3.15)	6.45 (-0.95 to 13.03)	8.34 (0.19 to 15.47)	9.87 (1.44 to 17.16)	13.78 (4.76 to 21.09)

Definition of abbreviations: TV = temperature variability; Q1 = the first quartile; Q2 = the second quartile; Q3 = the third quartile; Q4 = the fourth quartile; MMT = minimum mortality temperature; TVST = temperature variability sensitive heat threshold.

Table S12. Attributable fractions of mortality due to heat exposure stratified by TVST for each quartile of TV 0–3 in each country.

Country	Attributable fractions (%): MMT–TVST				Attributable fractions (%): above TVST			
	Q1 of TV 0–3	Q2 of TV 0–3	Q3 of TV 0–3	Q4 of TV 0–3	Q1 of TV 0–3	Q2 of TV 0–3	Q3 of TV 0–3	Q4 of TV 0–3
Argentina	1.49 (-0.23 to 3.12)	2.02 (-0.05 to 3.96)	2.67 (0.73 to 4.51)	2.65 (0.32 to 4.82)	9.41 (1.68 to 16.35)	15.26 (3.33 to 25.55)	24.77 (16.51 to 31.74)	19.30 (10.14 to 26.95)
Australia	0.70 (-0.28 to 1.63)	0.45 (-0.36 to 1.18)	0.90 (-0.34 to 2.08)	0.67 (-0.46 to 1.75)	5.26 (-1.40 to 11.36)	5.21 (-3.71 to 13.23)	10.60 (2.81 to 17.85)	12.20 (1.01 to 21.15)
Brazil	0.79 (-0.72 to 2.21)	1.67 (-0.80 to 3.92)	1.98 (-0.64 to 4.37)	2.68 (0.16 to 4.97)	14.68 (-4.24 to 28.84)	12.50 (-6.48 to 26.73)	17.70 (4.13 to 28.50)	23.30 (3.14 to 37.24)
Canada	0.69 (-0.47 to 1.78)	1.04 (-0.46 to 2.43)	1.39 (-0.27 to 2.94)	1.11 (-0.10 to 2.24)	7.48 (-1.79 to 15.51)	13.71 (2.21 to 23.42)	12.44 (1.30 to 21.70)	12.84 (-0.95 to 22.98)
China	0.79 (-0.92 to 2.47)	1.40 (-1.37 to 3.96)	1.93 (-0.99 to 4.62)	1.77 (-0.32 to 3.68)	8.70 (-3.28 to 19.41)	13.97 (-2.62 to 26.89)	14.64 (-2.06 to 27.60)	19.24 (0.40 to 32.59)
Colombia	0.67 (-0.67 to 1.94)	0.40 (-0.84 to 1.52)	0.83 (-1.19 to 2.71)	0.81 (-1.62 to 3.12)	6.42 (-4.55 to 16.35)	2.58 (-5.80 to 9.52)	6.51 (-6.46 to 18.19)	3.98 (-17.92 to 20.45)
Costa Rica	0.28 (-1.22 to 1.70)	0.47 (-1.69 to 2.48)	0.36 (-1.54 to 2.21)	0.55 (-1.94 to 2.87)	4.65 (-3.30 to 12.44)	5.31 (-6.04 to 15.54)	6.08 (-8.17 to 17.50)	6.46 (-5.32 to 15.79)
Czech Republic	0.35 (-0.39 to 1.05)	1.93 (0.14 to 3.56)	3.62 (1.15 to 5.90)	3.54 (1.24 to 5.70)	/	/	25.92 (16.50 to 34.46)	25.91 (12.49 to 36.25)
Ecuador	0.09 (-0.37 to 0.55)	0.92 (-1.67 to 3.30)	1.27 (-1.69 to 4.05)	0.64 (-0.93 to 2.11)	4.99 (-3.86 to 12.54)	11.83 (-9.61 to 27.14)	9.91 (-4.31 to 21.94)	11.67 (-4.02 to 24.18)
Estonia	0.17 (-0.67 to 0.98)	1.01 (-0.82 to 2.71)	1.01 (-1.28 to 3.17)	1.84 (-1.53 to 4.97)	8.64 (-4.86 to 20.41)	11.01 (-3.68 to 23.20)	11.31 (-6.37 to 24.96)	9.99 (-12.12 to 25.81)
France	0.33 (-1.70 to 2.08)	0.47 (-0.51 to 1.36)	0.76 (-0.47 to 1.89)	0.41 (-0.50 to 1.23)	3.74 (-2.38 to 9.83)	7.72 (0.34 to 14.33)	7.74 (1.23 to 13.75)	22.48 (17.75 to 26.55)
Germany	0.12 (-0.27 to 0.48)	0.68 (-0.42 to 1.68)	1.66 (0.02 to 3.17)	1.80 (0.11 to 3.37)	3.22 (-3.75 to 10.02)	9.54 (1.33 to 17.03)	13.09 (7.48 to 18.40)	20.35 (15.44 to 24.83)
Greece	1.00 (-0.25 to 2.19)	6.38 (3.84 to 8.51)	7.59 (4.60 to 10.23)	7.06 (2.98 to 10.65)	/	23.32 (16.97 to 29.14)	24.90 (19.01 to 30.14)	34.30 (26.37 to 41.28)
Guatemala	0.01 (-0.12 to 0.14)	0.13 (-0.80 to 1.01)	0.32 (-1.42 to 1.96)	0.20 (-0.78 to 1.12)	5.64 (-3.95 to 14.39)	8.27 (-6.84 to 20.10)	4.88 (-5.10 to 13.74)	3.13 (-1.64 to 7.68)
Iran	2.03 (-1.69 to 5.42)	2.68 (-0.99 to 6.05)	2.77 (-0.76 to 6.09)	4.35 (0.56 to 7.75)	/	19.22 (0.84 to 33.13)	27.80 (9.37 to 39.96)	31.85 (6.33 to 48.52)
Ireland	0.18 (-0.45 to 0.80)	0.12 (-0.25 to 0.48)	0.28 (-0.42 to 0.94)	0.62 (-1.09 to 2.22)	4.70 (-3.11 to 12.04)	2.42 (-5.51 to 9.99)	4.09 (-5.98 to 12.91)	4.64 (-6.44 to 13.85)
Italy	1.74 (-0.12 to 3.48)	4.15 (1.14 to 6.87)	4.38 (1.38 to 7.12)	3.77 (0.96 to 6.37)	14.67 (5.35 to 22.58)	24.74 (11.94 to 35.26)	27.61 (13.52 to 38.31)	23.87 (4.22 to 37.48)
Japan	1.01 (0.08 to 1.88)	1.57 (0.32 to 2.73)	1.60 (0.41 to 2.73)	1.46 (0.40 to 2.44)	9.02 (3.32 to 14.47)	9.28 (4.01 to 14.34)	10.39 (5.15 to 15.26)	9.83 (4.42 to 14.90)
Mexico	0.11 (-0.29 to 0.48)	0.45 (-0.72 to 1.50)	0.63 (-0.46 to 1.65)	1.55 (-0.68 to 3.65)	24.72 (-16.70 to 59.26)	15.71 (-10.17 to 36.41)	8.65 (-1.11 to 17.22)	10.40 (2.67 to 17.09)
Moldova	0.06 (-0.27 to 0.40)	0.82 (-1.24 to 2.75)	0.67 (-1.13 to 2.46)	1.19 (-0.41 to 2.80)	/	2.90 (-0.98 to 6.10)	4.75 (-2.51 to 10.82)	16.81 (1.77 to 28.16)
Netherland	0.05 (-0.28 to 0.38)	0.59 (-0.81 to 1.88)	0.89 (-0.92 to 2.57)	1.14 (-0.50 to 2.70)	3.59 (-3.25 to 10.16)	7.56 (-1.29 to 15.52)	9.76 (0.75 to 17.83)	20.07 (12.02 to 26.82)
Panama	0.75 (-1.46 to 2.91)	2.87 (-2.41 to 7.48)	3.70 (-4.94 to 10.89)	1.39 (-1.62 to 4.28)	/	24.92 (-20.23 to 54.12)	30.34 (-28.17 to 69.57)	/
Peru	1.10 (-1.32 to 3.43)	1.40 (-1.55 to 4.13)	1.67 (-1.44 to 4.54)	0.80 (-0.90 to 2.26)	12.91 (-5.95 to 27.13)	19.26 (-13.71 to 43.46)	23.22 (-7.23 to 43.51)	28.06 (-31.42 to 61.97)
Portugal	0.14 (-0.21 to 0.47)	0.80 (-0.08 to 1.59)	1.90 (0.59 to 3.07)	1.24 (-0.18 to 2.60)	6.55 (0.78 to 12.20)	13.11 (6.92 to 18.76)	15.35 (10.29 to 20.08)	21.38 (17.27 to 25.03)
Puerto Rico	0.45 (-1.61 to 2.44)	0.61 (-1.90 to 2.96)	0.19 (-0.72 to 1.00)	1.08 (-2.31 to 4.17)	4.39 (-2.33 to 10.35)	9.85 (-11.98 to 28.79)	6.71 (-15.50 to 24.48)	9.85 (-17.64 to 28.07)
Romania	0.80 (-0.28 to 1.81)	1.83 (0.08 to 3.47)	3.88 (1.60 to 6.03)	4.08 (1.99 to 6.05)	12.50 (1.95 to 21.13)	21.35 (8.97 to 31.98)	20.10 (10.48 to 28.24)	29.65 (18.45 to 38.14)
South Africa	0.35 (-0.78 to 1.44)	0.61 (-0.96 to 2.06)	0.82 (-1.54 to 2.99)	1.14 (-1.40 to 3.44)	6.80 (-2.43 to 15.14)	7.51 (-5.37 to 18.23)	7.29 (-5.82 to 17.85)	7.90 (-7.58 to 19.30)
South Korea	0.48 (-0.60 to 1.52)	0.81 (-0.81 to 2.31)	0.50 (-0.48 to 1.43)	0.27 (-0.27 to 0.77)	7.16 (-2.06 to 15.28)	8.12 (-0.57 to 15.88)	7.45 (-1.79 to 15.09)	10.85 (-3.68 to 21.71)

Spain	1.17 (-0.04 to 2.29)	2.68 (0.31 to 4.83)	4.01 (1.06 to 6.67)	3.58 (0.16 to 6.63)	9.40 (3.21 to 15.36)	15.76 (8.34 to 22.22)	18.76 (10.62 to 25.84)	22.46 (13.00 to 30.05)
Sweden	0.03 (-0.07 to 0.14)	0.36 (-0.63 to 1.30)	1.04 (-0.62 to 2.59)	1.74 (-0.12 to 3.51)	/	8.82 (-1.55 to 17.97)	11.29 (2.28 to 19.23)	12.98 (2.93 to 21.33)
Switzerland	0.17 (-0.56 to 0.87)	0.83 (-1.38 to 2.89)	1.55 (-1.62 to 4.55)	3.49 (-0.53 to 7.25)	8.06 (-1.71 to 16.24)	9.95 (-7.91 to 24.32)	11.70 (-3.51 to 23.99)	17.88 (1.12 to 30.42)
Thailand	0.32 (-1.04 to 1.62)	0.79 (-1.87 to 3.24)	2.03 (-1.72 to 5.55)	2.09 (-1.40 to 5.32)	7.85 (-3.17 to 17.65)	8.55 (-9.02 to 22.34)	13.67 (-4.89 to 27.93)	14.97 (-8.94 to 31.70)
UK	0.11 (-0.26 to 0.46)	0.23 (-0.43 to 0.84)	0.50 (-0.42 to 1.37)	1.73 (0.10 to 3.25)	6.25 (-3.61 to 14.34)	7.26 (-7.40 to 18.76)	9.64 (-2.31 to 19.37)	16.05 (2.15 to 26.09)
Uruguay	0.70 (-0.26 to 1.60)	1.75 (-0.32 to 3.62)	3.35 (0.53 to 5.93)	2.20 (-0.94 to 5.10)	/	15.56 (4.84 to 24.68)	26.13 (14.88 to 35.15)	10.19 (-13.37 to 26.51)
USA	0.61 (-0.70 to 1.86)	0.85 (-0.70 to 2.29)	0.94 (-0.56 to 2.34)	1.01 (-0.51 to 2.43)	7.69 (-0.06 to 14.78)	9.99 (0.36 to 18.45)	11.04 (1.20 to 19.43)	11.34 (0.18 to 20.17)
Vietnam	1.90 (-0.72 to 4.36)	2.41 (-1.13 to 5.69)	3.62 (-0.27 to 7.20)	1.59 (-1.03 to 4.07)	23.76 (11.25 to 34.12)	15.94 (1.31 to 27.45)	25.55 (10.88 to 37.58)	23.59 (-1.58 to 40.36)
Total	0.69 (-0.37 to 1.69)	1.14 (-0.31 to 2.48)	1.37 (-0.19 to 2.83)	1.44 (-0.13 to 2.88)	8.26 (0.90 to 15.02)	9.69 (1.87 to 16.68)	10.90 (3.17 to 17.64)	13.82 (4.97 to 20.99)

Definition of abbreviations: TV = temperature variability; Q1 = the first quartile; Q2 = the second quartile; Q3 = the third quartile; Q4 = the fourth quartile; MMT = minimum mortality temperature; TVST = temperature variability sensitive heat threshold.

Table S13. Sensitivity analyses of overall attributable fractions after adding separate predictor in meta-regression with BLUP, stratified by quartiles of TV.

Predictors	Attributable fractions (%)			
	Q1 of TV	Q2 of TV	Q3 of TV	Q4 of TV
Without predictors	0.70 (-0.33 to 1.69)	1.34 (-0.14 to 2.73)	1.99 (0.29 to 3.57)	2.73 (0.76 to 4.50)
Region	0.79 (-0.27 to 1.76)	1.35 (0.06 to 2.55)	1.99 (0.47 to 3.40)	2.88 (1.01 to 4.56)
Average mean temperature	0.75 (-0.27 to 1.72)	1.37 (-0.13 to 2.78)	2.00 (0.30 to 3.58)	2.74 (0.75 to 4.52)
Range of mean temperature	0.71 (-0.33 to 1.72)	1.36 (-0.16 to 2.80)	1.99 (0.26 to 3.60)	2.74 (0.73 to 4.54)
Koeppen-Geiger Climatic Zones 1st	0.75 (-0.45 to 1.88)	1.39 (-0.22 to 2.88)	2.02 (0.19 to 3.70)	2.76 (0.66 to 4.64)
Koeppen-Geiger Climatic Zones 2nd	0.74 (-0.33 to 1.72)	1.39 (-0.15 to 2.81)	2.03 (0.20 to 3.71)	2.81 (0.67 to 4.71)
Koeppen-Geiger Climatic Zones 3rd	0.76 (-0.37 to 1.79)	1.39 (-0.17 to 2.83)	2.04 (0.18 to 3.75)	2.83 (0.59 to 4.82)
GDP per capita ($)	0.72 (-0.34 to 1.72)	1.35 (-0.16 to 2.77)	2.00 (0.27 to 3.60)	2.75 (0.74 to 4.55)
Latitude	0.72 (-0.33 to 1.72)	1.36 (-0.17 to 2.80)	1.98 (0.26 to 3.58)	2.73 (0.76 to 4.50)
longitude	0.70 (-0.32 to 1.66)	1.35 (-0.13 to 2.72)	1.99 (0.31 to 3.55)	2.72 (0.73 to 4.51)

Definition of abbreviations: BLUP = the best linear unbiased prediction; TV = temperature variability; Q1 = the first quartile; Q2 = the second quartile; Q3 = the third quartile; Q4 = the fourth quartile.

Table S14. Sensitivity analyses of overall attributable fractions after changing the lag of mean temperature in the main model, stratified by quartiles of TV.

Lag	Attributable fractions (%)			
	Q1 of TV	Q2 of TV	Q3 of TV	Q4 of TV
7	0.80 (-0.34 to 1.83)	1.56 (-0.05 to 3.03)	2.24 (0.51 to 3.84)	2.99 (1.01 to 4.78)
8	0.78 (-0.34 to 1.80)	1.48 (-0.06 to 2.90)	2.14 (0.42 to 3.74)	2.87 (0.93 to 4.61)
9	0.72 (-0.35 to 1.72)	1.39 (-0.11 to 2.80)	2.07 (0.35 to 3.67)	2.81 (0.82 to 4.61)
10 [a]	0.70 (-0.33 to 1.69)	1.34 (-0.14 to 2.73)	1.99 (0.29 to 3.57)	2.73 (0.76 to 4.50)
11	0.67 (-0.34 to 1.62)	1.28 (-0.16 to 2.64)	1.92 (0.25 to 3.46)	2.62 (0.67 to 4.37)
12	0.64 (-0.37 to 1.58)	1.24 (-0.21 to 2.60)	1.86 (0.19 to 3.41)	2.54 (0.58 to 4.30)
13	0.62 (-0.36 to 1.55)	1.20 (-0.25 to 2.57)	1.80 (0.13 to 3.35)	2.47 (0.52 to 4.21)

Definition of abbreviations: TV = temperature variability; Q1 = the first quartile; Q2 = the second quartile; Q3 = the third quartile; Q4 = the fourth quartile.

[a] lag day used in the primary model.

Table S15. Sensitivity analyses of overall attributable fractions using different definitions of warm season, stratified by quartiles of TV.

Warm season	Attributable fractions (%)			
	Q1 of TV	Q2 of TV	Q3 of TV	Q4 of TV
two consecutive months	1.63 (-1.33 to 4.32)	2.28 (-1.21 to 5.33)	3.06 (-0.80 to 6.48)	4.69 (0.31 to 8.49)
three consecutive months	1.00 (-0.38 to 2.37)	1.53 (-0.20 to 3.17)	2.26 (0.14 to 4.19)	3.13 (0.75 to 5.32)
four consecutive months [a]	0.70 (-0.33 to 1.69)	1.34 (-0.14 to 2.73)	1.99 (0.29 to 3.57)	2.73 (0.76 to 4.50)

Definition of abbreviations: TV = temperature variability; Q1 = the first quartile; Q2 = the second quartile; Q3 = the third quartile; Q4 = the fourth quartile.

[a] warm season used in the primary model.

Table S16. Sensitivity analyses of overall attributable fractions after adjusting relative humidity in the main model using 556 cities with relative humidity data, stratified by quartiles of TV.

Model	Attributable fractions (%)			
	Q1 of TV	Q2 of TV	Q3 of TV	Q4 of TV
Without relative humidity	0.68 (-0.26 to 1.56)	1.34 (-0.05 to 2.64)	2.00 (0.44 to 3.46)	2.66 (0.99 to 4.17)
With relative humidity	0.73 (-0.29 to 1.69)	1.41 (-0.03 to 2.74)	2.08 (0.49 to 3.55)	2.73 (0.96 to 4.32)

Definition of abbreviations: TV = temperature variability; Q1 = the first quartile; Q2 = the second quartile; Q3 = the third quartile; Q4 = the fourth quartile.

Table S17. Sensitivity analyses of overall attributable fractions using different complete data after removing cities with missing vlaues or after imputaion, stratified by quartiles of TV.

Dataset	Attributable fractions (%)			
	Q1 of TV	Q2 of TV	Q3 of TV	Q4 of TV
complete data after removing cities with missing values	0.59 (-0.41 to 1.51)	1.10 (-0.30 to 2.41)	1.69 (0.14 to 3.11)	2.65 (0.70 to 4.37)
complete data after imputation	0.71 (-0.39 to 1.73)	1.35 (-0.14 to 2.75)	1.99 (0.28 to 3.57)	2.73 (0.79 to 4.48)
primary results	0.70 (-0.33 to 1.69)	1.34 (-0.14 to 2.73)	1.99 (0.29 to 3.57)	2.73 (0.76 to 4.50)

Definition of abbreviations: TV = temperature variability; Q1 = the first quartile; Q2 = the second quartile; Q3 = the third quartile; Q4 = the fourth quartile.

Reference:

1. Zhao Q, Guo Y, Ye T, et al. Global, regional, and national burden of mortality associated with non-optimal ambient temperatures from 2000 to 2019: a three-stage modelling study. *The Lancet Planetary health* 2021; **5**(7): e415-e25.
2. Xu R, Xiong X, Abramson MJ, Li S, Guo Y. Ambient temperature and intentional homicide: A multi-city case-crossover study in the US. *Environ Int* 2020; **143**: 105992.
3. Meng X, Liu C, Chen R, et al. Short term associations of ambient nitrogen dioxide with daily total, cardiovascular, and respiratory mortality: multilocation analysis in 398 cities. *BMJ (Clinical research ed)* 2021; **372**: n534.
