# Supplementary material for: Fluctuating temperature modifies heat-mortality association around the globe
Source: Innovation (Camb). 2022 Mar 11;3(2):100225. doi: 10.1016/j.xinn.2022.100225 (PMC8942841; doi:10.1016/j.xinn.2022.100225)
Supplement: Supplementary file 2 [file mmc4.pdf]

www.the-innovation.org

Fluctuating temperature modifies heat-mortality association around the globe

Yao Wu,[1,2] Bo Wen,[1,2] Shanshan Li,[1,2,*] Antonio Gasparrini,[3,4,5] Shilu Tong,[6,7,8,9] Ala Overcenco,[10] Aleš Urban,[11,12] Alexandra Schneider,[13] Alireza Entezari,[2,14] Ana Maria Vicedo-Cabrera,[3,15,16] Antonella Zanobetti,[17] Antonis Analitis,[18] Ariana Zeka,[19] Aurelio Tobias,[20,21] Barrak Alahmad,[17] Ben Armstrong,[3] Bertil Forsberg,[22] Carmen Íñiguez,[23,24] Caroline Ameling,[25] César De la Cruz Valencia,[26] Christofer Åström,[22] Danny Houthuijs,[25] Do Van Dung,[27] Dominic Royé,[24,28] Ene Indermitte,[29] Eric Lavigne,[30,31] Fatemeh Mayvaneh,[14] Fiorella Acquaotta,[32] Francesca de'Donato,[33] Francesco Sera,[34] Gabriel Carrasco-Escobar,[35,36] Haidong Kan,[37] Hans Orru,[29] Ho Kim,[38] Iulian-Horia Holobaca,[39] Jan Kyselý,[11,12] Joana Madureira,[40,41,42] Joel Schwartz,[17] Klea Katsouyanni,[18,43] Magali Hurtado-Diaz,[26] Martina S. Ragettli,[44,45] Masahiro Hashizume,[46] Mathilde Pascal,[47] Micheline de Sousa Zanotti Stagliorio Coélho,[48] Noah Scovronick,[49] Paola Michelozzi,[33] Patrick Goodman,[50] Paulo Hilario Nascimento Saldiva,[51] Rosana Abrutzky,[52] Samuel Osorio,[53] Tran Ngoc Dang,[27] Valentina Colistro,[54] Veronika Huber,[55,56] Whanhee Lee,[57,58] Xerxes Seposo,[21] Yasushi Honda,[59] Michelle L. Bell,[57] and Yuming Guo[1,2,*]

*Correspondence: yuming.guo@monash.edu (Y.G.); shanshan.li@monash.edu (S.L.)

Received: December 16, 2021; Accepted: March 2, 2022; Published Online: March 11, 2022; https://doi.org/10.1016/j.xinn.2022.100225

© 2022 The Author(s). This is an open access article under the CC BY license (http://creativecommons.org/licenses/by/4.0/).

GRAPHICAL ABSTRACT

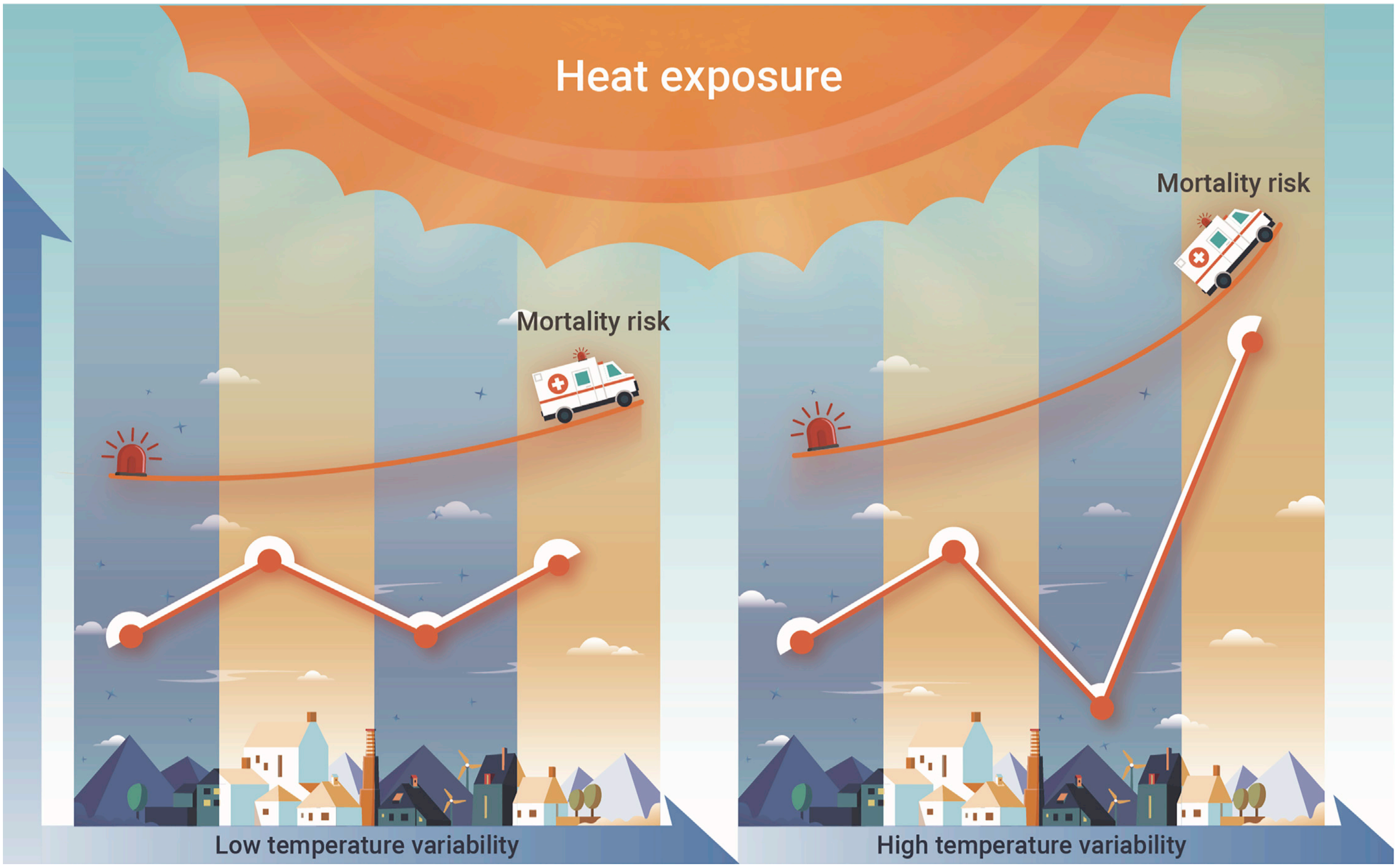

PUBLIC SUMMARY

- Increased temperature variability (TV) poses a greater mortality risk due to heat
- TV has a more profound modification effect on extreme heat-mortality association
- Strategies against heat and TV simultaneously would benefit public health

Fluctuating temperature modifies heat-mortality association around the globe

Yao Wu,[1,2] Bo Wen,[1,2] Shanshan Li,[1,2,*] Antonio Gasparrini,[3,4,5] Shilu Tong,[6,7,8,9] Ala Overcenco,[10] Aleš Urban,[11,12] Alexandra Schneider,[13] Alireza Entezari,[2,14] Ana Maria Vicedo-Cabrera,[3,15,16] Antonella Zanobetti,[17] Antonis Analitis,[18] Ariana Zeka,[19] Aurelio Tobias,[20,21] Barrak Alahmad,[17] Ben Armstrong,[3] Bertil Forsberg,[22] Carmen Íñiguez,[23,24] Caroline Ameling,[25] César De la Cruz Valencia,[26] Christofer Åström,[22] Danny Houthuijs,[25] Do Van Dung,[27] Dominic Royé,[24,28] Ene Indermitte,[29] Eric Lavigne,[30,31] Fatemeh Mayvaneh,[14] Fiorella Acquaotta,[32]

(Author list continued on next page)

Studies have investigated the effects of heat and temperature variability (TV) on mortality. However, few assessed whether TV modifies the heat-mortality association. Data on daily temperature and mortality in the warm season were collected from 717 locations across 36 countries. TV was calculated as the standard deviation of the average of the same and previous days' minimum and maximum temperatures. We used location-specific quasi-Poisson regression models with an interaction term between the cross-basis term for mean temperature and quartiles of TV to obtain heat-mortality associations under each quartile of TV, and then pooled estimates at the country, regional, and global levels. Results show the increased risk in heat-related mortality with increments in TV, accounting for 0.70% (95% confidence interval [CI]: −0.33 to 1.69), 1.34% (95% CI: −0.14 to 2.73), 1.99% (95% CI: 0.29–3.57), and 2.73% (95% CI: 0.76–4.50) of total deaths for Q1–Q4 (first quartile–fourth quartile) of TV. The modification effects of TV varied geographically. Central Europe had the highest attributable fractions (AFs), corresponding to 7.68% (95% CI: 5.25–9.89) of total deaths for Q4 of TV, while the lowest AFs were observed in North America, with the values for Q4 of 1.74% (95% CI: −0.09 to 3.39). TV had a significant modification effect on the heat-mortality association, causing a higher heat-related mortality burden with increments of TV. Implementing targeted strategies against heat exposure and fluctuant temperatures simultaneously would benefit public health.

INTRODUCTION

Global warming pervasively affects human life and undermines the years of gains in public health.[1,2] Owing to the increasing rate of 0.2°C in temperature per decade, human-induced warming has been associated with an increase in frequency and intensity of hot days, reaching 2.9 billion additional person-days of exposure to heatwave events of vulnerable populations older than 65 years in 2019.[3–6] Increasing heat exposure, in turn, results in excess morbidity or mortality.[7,8] For the past 20 years, an average of 489,075 heat-related excess deaths per year was estimated at the global level, and the heat-related excess death ratio increased from 0.83% (95% confidence interval [CI]: 0.52–1.25) in 2000–2003 to 1.04% (95% CI: 0.64–1.55) in 2016–2019.[9] During 1991–2018, 37.0% (95% CI: 20.5–76.3) of heat-related deaths in the warm season can be attributed to human-induced heating.[10]

Another challenging issue driven by climate change is temperature variability (TV), an indicator of short-term temperature fluctuations or stability. Previous studies have projected an increasing trend of TV in some regions, in particular in tropical countries.[11,12] Extensive studies have established evidence of the health effects of TV, showing a significant association between TV and mortality in many parts of the world and substantial public health burden stemming from TV.[13,14] Traditionally, studies of the health effects of TV have controlled for mean temperature as a confounder.[13,15,16] Based on similar biological mechanisms underlying the health effects of TV and heat exposure, it is worth investigating whether there exists a synergistic effect between them.[17] Previous studies observed season-differentiated effects of TV on mortality, which suggests potential effect modification of mean temperature on TV-related mortality.[13,18] However, to the best of our knowledge, few studies have examined whether TV modifies the heat-mortality association. For example, the temperatures fluctuations from 5°C to 25°C and from 13°C to 17°C represent the same mean temperature of 15°C, but their effects on mortality are very likely to be different. Assessing heat-mortality association without considering the modification of TV may fail to recognize the most severe weather in relation to heat and to implement an effective early warning system.

[1]Department of Epidemiology and Preventive Medicine, School of Public Health and Preventive Medicine, Monash University, Melbourne 3004, Australia
[2]Climate, Air Quality Research Unit, School of Public Health and Preventive Medicine, Monash University, Melbourne 3004, Australia
[3]Department of Public Health, Environments, and Society, London School of Hygiene & Tropical Medicine, London WC1E 7HT, UK
[4]Centre for Statistical Methodology, London School of Hygiene & Tropical Medicine, London WC1E 7HT, UK
[5]Centre on Climate Change & Planetary Health, London School of Hygiene & Tropical Medicine, London WC1E 7HT, UK
[6]Shanghai Children's Medical Centre, Shanghai Jiao Tong University, Shanghai 200025, China
[7]School of Public Health, Institute of Environment and Human Health, Anhui Medical University, Hefei 230032, China
[8]Center for Global Health, Nanjing Medical University, Nanjing 211166, China
[9]School of Public Health and Social Work, Queensland University of Technology, Brisbane 4000, Australia
[10]National Agency for Public Health of the Ministry of Health, Labour, and Social Protection of the Republic of Moldova, Chisinau 2009, Republic of Moldova
[11]Institute of Atmospheric Physics, Academy of Sciences of the Czech Republic, Prague 117 20, Czech Republic
[12]Institute of Epidemiology, Helmholtz Zentrum München–German Research Center for Environmental Health, Neuherberg 85747, Germany
[13]Faculty of Geography and Environmental Sciences, Hakim Sabzevari University, Sabzevar 9617976487, Iran
[14]Institute of Social and Preventive Medicine, University of Bern, Bern 3012, Switzerland
[15]Oeschger Center for Climate Change Research, University of Bern, Bern 3012, Switzerland
[16]Department of Environmental Health, Harvard T.H. Chan School of Public Health, Harvard University, Boston, MA 02115, USA
[17]Department of Hygiene, Epidemiology and Medical Statistics, National and Kapodistrian University of Athens, Athens 11527, Greece
[18]Institute for Environment, Health, and Societies, Brunel University London, London UB8 3PN, UK
[19]Institute of Environmental Assessment and Water Research, Spanish Council for Scientific Research, Barcelona 08034, Spain
[20]School of Tropical Medicine and Global Health, Nagasaki University, Nagasaki 852-8521, Japan
[21]Department of Public Health and Clinical Medicine, Umeå University, Umeå 901 87, Sweden
[22]Department of Statistics and Computational Research, Universitat de València, València 46003, Spain
[23]CIBER de Epidemiología y Salud Pública (CIBERESP), Madrid 28029, Spain
[24]National Institute for Public Health and the Environment (RIVM), Centre for Sustainability and Environmental Health, Bilthoven 3720 BA, Netherlands

(Affiliations continued on next page)

Francesca de'Donato,[33] Francesco Sera,[34] Gabriel Carrasco-Escobar,[35,36] Haidong Kan,[37] Hans Orru,[29] Ho Kim,[38] Iulian-Horia Holobaca,[39] Jan Kyselý,[11,12] Joana Madureira,[40,41,42] Joel Schwartz,[17] Klea Katsouyanni,[18,43] Magali Hurtado-Diaz,[26] Martina S. Ragettli,[44,45] Masahiro Hashizume,[46] Mathilde Pascal,[47] Micheline de Sousa Zanotti Stagliorio Coélho,[48] Noah Scovronick,[49] Paola Michelozzi,[33] Patrick Goodman,[50] Paulo Hilario Nascimento Saldiva,[51] Rosana Abrutzky,[52] Samuel Osorio,[53] Tran Ngoc Dang,[27] Valentina Colistro,[54] Veronika Huber,[55,56] Whanhee Lee,[57,58] Xerxes Seposo,[21] Yasushi Honda,[59] Michelle L. Bell,[57] Yuming Guo[1,2,*]

www.the-innovation.org

In this study, using data from the Multi-Country Multi-City (MCC) Collaborative Research Network, we systematically evaluated the contribution of TV to the heat-mortality association in 717 locations across 36 countries over the period 1972–2018. Through this study, we aimed to provide a more complete picture of the TV-differentiated heat-related mortality burden and to provide scientific evidence that could improve the sensitivity of current heat-health warning systems in hot temperatures with dramatic temperature fluctuations.

RESULTS

The descriptive statistics from each country are shown in Table 1. A total of 36.42 million deaths from total or non-external causes were identified during the warm season from 1972 to 2018 (Table 1). On average, the median daily TV across 717 locations was 5.8°C (25th–75th percentile: 4.9–6.7). The average daily mean temperature under each quartile of TV was 20.6°C (Q1), 21.7°C (Q2), 22.3°C (Q3), and 23.0°C (Q4) (Table 1). The overall correlation coefficient between daily mean temperature and TV was 0.10 (Table S2). The summary descriptions of the daily mean temperature and TV for each location are shown in Tables S3–S5.

Figure 1 shows exposure-response curves between daily mean temperature and mortality in warm season. In general, J-shaped associations between daily mean temperature and mortality were found under different groups of TV, with the risks increasing dramatically at extreme hot temperatures (Figure 1A). The MMTs for different quartiles of TV were 17.62°C (Q1), 17.62°C (Q2), 18.39°C (Q3), and 19.80°C (Q4). The difference in four curves was tested as statistically significant. Regional exposure-response curves indicate potential geographical patterns in the modification effect of TV on the heat-mortality association (Figure 1B). In general, higher mortality risks were observed for higher TV levels across different regions. Southern Europe and central Europe generated a greater difference in heat-related mortality risks between Q1 and Q4 of TV. For most countries, the country-specific curve demonstrated increased mortality risks as TV rose (Figure S1).

From the Q1 to the Q4 of TV, there was an increasing trend of AF due to heat exposure, with a value of 0.70% (95% CI: −0.33 to 1.69) for Q1, 1.34% (95% CI: −0.14 to 2.73) for Q2, 1.99% (95% CI: 0.29–3.57) for Q3, and 2.73% (95% CI: 0.76–4.50) for Q4 (Table 2). The corresponding attributable deaths are shown in Table S6. Central Europe had the highest AFs for Q4 of TV among all of the regions. The country-specific AFs stratified by TV groups are shown in Table S7. Dividing the AF according to the country-specific TVST (an average of the 96.34th percentile across all of the countries), temperatures between MMT and TVST were responsible for a small fraction, increasing from 0.66% (95% CI: −0.35 to 1.62) for Q1 to 1.83% (95% CI: 0.17–3.37) for Q4 of TV. AF for temperatures above TVST changed dramatically, with increasing AFs of 8.61%

[25]Department of Environmental Health, National Institute of Public Health, Cuernavaca Morelos 62100, Mexico
[26]Department of Environmental Health, Faculty of Public Health, University of Medicine and Pharmacy at Ho Chi Minh City, Ho Chi Minh City 17000, Vietnam
[27]Department of Geography, University of Santiago de Compostela, Santiago de Compostela 15705, Spain
[28]Institute of Family Medicine and Public Health, University of Tartu, Tartu 50090, Estonia
[29]School of Epidemiology & Public Health, Faculty of Medicine, University of Ottawa, Ottawa, ON K1N 6N5, Canada
[30]Air Health Science Division, Health Canada, Ottawa, ON K1A 0K9, Canada
[31]Department of Earth Sciences, University of Turin, Turin 10125, Italy
[32]Department of Epidemiology, Lazio Regional Health Service, Rome 00147, Italy
[33]Department of Statistics, Computer Science, and Applications "G. Parenti", University of Florence, Florence 50121, Italy
[34]Institute of Tropical Medicine "Alexander von Humboldt", Universidad Peruana Cayetano Heredia, Lima 15102, Peru
[35]Department of Medicine, Division of Infectious Diseases, University of California, San Diego, San Diego, CA 92093, USA
[36]Department of Environmental Health, School of Public Health, Fudan University, Shanghai 200032, China
[37]Graduate School of Public Health, Seoul National University, Seoul 08826, Republic of Korea
[38]Faculty of Geography, Babeş-Bolyai University, Cluj-Napoca 400084, Romania
[39]Faculty of Environmental Sciences, Czech University of Life Sciences, Prague 165 00, Czech Republic
[40]EPIUnit–Instituto de Saúde Pública, Universidade do Porto, Porto 4099-002, Portugal
[41]Laboratório para a Investigação Integrativa e Translacional em Saúde Populacional (ITR), Porto 4050-600, Portugal
[42]School of Population Health and Environmental Sciences, King's College London, London WC2R 2LS, UK
[43]Swiss Tropical and Public Health Institute, Basel 4051, Switzerland
[44]University of Basel, Basel 4001, Switzerland
[45]Department of Global Health Policy, Graduate School of Medicine, The University of Tokyo, Tokyo 113-8654, Japan
[46]Santé Publique France, Department of Environmental Health, French National Public Health Agency, Saint Maurice 94 410, France
[47]Department of Pathology, Faculty of Medicine, University of São Paulo, São Paulo 05508-270, Brazil
[48]Department of Environmental Health, Rollins School of Public Health, Emory University, Atlanta, GA 30322, USA
[49]Technological University Dublin, Dublin D07 EWV4, Ireland
[50]INSPER, São Paulo 04546-042, Brazil
[51]Instituto de Investigaciones Gino Germani, Facultad de Ciencias Sociales, Universidad de Buenos Aires, Buenos Aires C1053ABH, Argentina
[52]Department of Environmental Health, University of São Paulo, São Paulo 01246-904, Brazil
[53]Institute of Research and Development, Duy Tan University, Da Nang 550000, Vietnam
[54]Department of Quantitative Methods, School of Medicine, University of the Republic, Montevideo 11200, Uruguay
[55]IBE-Chair of Epidemiology, Ludwig Maximilian University Munich, Munich 81377, Germany
[56]Department of Physical, Chemical, and Natural Systems, Universidad Pablo de Olavide, Sevilla 41013, Spain
[57]School of the Environment, Yale University, New Haven, CT 06511, USA
[58]Center for Climate Change Adaptation, National Institute for Environmental Studies, Tsukuba, Ibaraki 305-8506, Japan
*Correspondence: yuming.guo@monash.edu (Y.G.), shanshan.li@monash.edu (S.L.)
Received: December 16, 2021; Accepted: March 2, 2022; Published Online: March 11, 2022; https://doi.org/10.1016/j.xinn.2022.100225
© 2022 The Author(s). This is an open access article under the CC BY license (http://creativecommons.org/licenses/by/4.0/).
Citation: Wu Y., Wen B., Li S., et al., (2022). Fluctuating temperature modifies heat-mortality association around the globe. The Innovation **3(2)**, 100225.

Table 1. Mortality data and description of daily TV and mean temperature in each stratum (from Q1 to Q4) of TV in 717 locations from 36 countries during the warm season

Country	No. cities	Total death (thousands)	Temperature variability (°C) Median (P_{25}–P_{75})	Mean temperature (°C) Q1	Q2	Q3	Q4	Difference between Q4 and Q1 of TV
Argentina	3	199	7.1 (6.1–8.1)	22.3	23.6	24.2	24.8	2.4
Australia	3	348	4.5 (3.7–5.6)	21.2	21.7	22.4	23.2	2.0
Brazil	18	1,064	5.5 (4.8–6.2)	25.0	25.8	26.2	26.5	1.5
Canada	26	1,135	6.4 (5.3–7.6)	16.4	17.3	17.7	18.5	2.1
China	12	284	5.2 (4.3–6.2)	24.1	25.2	25.6	25.7	1.6
Colombia	5	291	5.5 (4.9–6.1)	21.9	22.4	22.7	23.0	1.1
Costa Rica	1	10	5.5 (4.9–6.2)	23.0	23.3	23.5	23.6	0.6
Czech Republic	4	227	4.3 (3.3–5.2)	14.3	16.5	18.5	20.1	5.8
Ecuador	2	33	4.9 (4.2–5.7)	21.0	21.4	21.6	21.7	0.7
Estonia	5	46	5.7 (4.4–7.0)	14.0	14.9	15.9	17.1	3.2
France	18	513	5.9 (4.8–7.1)	17.6	18.6	19.7	21.4	3.8
Germany	12	974	5.9 (4.7–7.3)	14.8	16.5	18.2	20.7	6.0
Greece	1	82	5.3 (4.7–5.9)	25.5	26.6	27.9	29.1	3.7
Guatemala	1	21	5.0 (4.5–5.8)	19.8	20.5	20.7	20.9	1.1
Iran	1	41	8.7 (7.9–9.6)	25.2	26.1	26.2	26.0	0.8
Ireland	6	317	4.4 (3.7–5.2)	14.0	14.1	14.1	14.6	0.6
Italy	17	246	4.6 (4.0–5.4)	22.5	23.6	24.1	24.5	2.0
Japan	47	12,049	4.7 (3.9–5.5)	23.0	24.4	25.0	24.6	1.6
Mexico	10	757	7.5 (6.3–8.6)	21.3	22.7	23.5	24.1	2.8
Moldova	4	19	7.2 (6.1–8.4)	18.2	20.2	21.3	22.7	4.5
Netherland	5	142	5.5 (4.4–6.8)	15.4	16.0	16.8	18.9	3.6
Panama	1	2	4.7 (4.0–5.5)	28.1	28.9	29.2	28.7	0.5
Peru	18	174	6.6 (5.8–7.5)	19.7	20.2	20.2	20.1	0.4
Portugal	5	499	7.6 (6.3–8.9)	19.0	20.6	21.9	24.0	5.0
Puerto Rico	1	8	3.9 (3.6–4.3)	28.2	28.1	28.1	28.4	0.2
Romania	8	300	7.4 (6.3–8.5)	17.9	20.0	21.2	22.4	4.5
South Africa	52	2,148	7.8 (6.6–8.9)	21.0	22.2	22.7	23.1	2.0
South Korea	36	967	4.9 (3.9–6.0)	22.9	23.8	23.9	22.7	−0.2
Spain	45	830	7.7 (6.6–8.7)	19.4	21.5	22.6	23.8	4.4
Sweden	3	220	4.7 (3.6–6.0)	14.7	15.6	16.4	18.3	3.6
Switzerland	8	75	5.5 (4.3–6.5)	15.2	17.1	18.7	20.6	5.4
Thailand	62	571	5.5 (4.8–6.4)	28.0	28.8	29.3	29.6	1.6
UK	65	1,784	5.1 (4.1–6.3)	15.0	15.4	15.7	16.6	1.6
Uruguay	1	45	4.9 (3.6–5.9)	21.4	23.8	25.2	25.9	4.5
USA	209	9,968	7.0 (6.1–7.9)	22.1	22.9	23.1	23.0	0.9
Vietnam	2	38	5.5 (4.9–6.0)	28.5	29.3	29.6	30.2	1.6
Total	717	36,424	5.8 (4.9–6.7)	20.6	21.7	22.3	23.0	2.4

IQR = interquartile range; Q1 = the 1st quartile; Q2 = the 2nd quartile; Q3 = the 3rd quartile; Q4 = the 4th quartile; P_{25} = the 25th percentile; P_{75} = the 75th percentile; TV = temperature variability.

(95% CI: −1.82 to 17.49), 10.48% (95% CI: −0.02 to 19.25), 12.90% (95% CI: 2.70–21.53), and 16.36% (95% CI: 5.81–24.62) for Q1–Q4 of TV, respectively (Table S8). Country-specific TVST ranged between the 87.47th percentile and the 100th percentile (Figure 2A). For most countries, AFs for the Q4 of TV were higher than the AFs for Q1 of TV, especially for temperatures above TVST (Figure 2B; Table S8).

In the sensitivity analyses, our results were robust. The same patterns were observed after changing the length of exposure to TV (Figures S2–S5; Tables

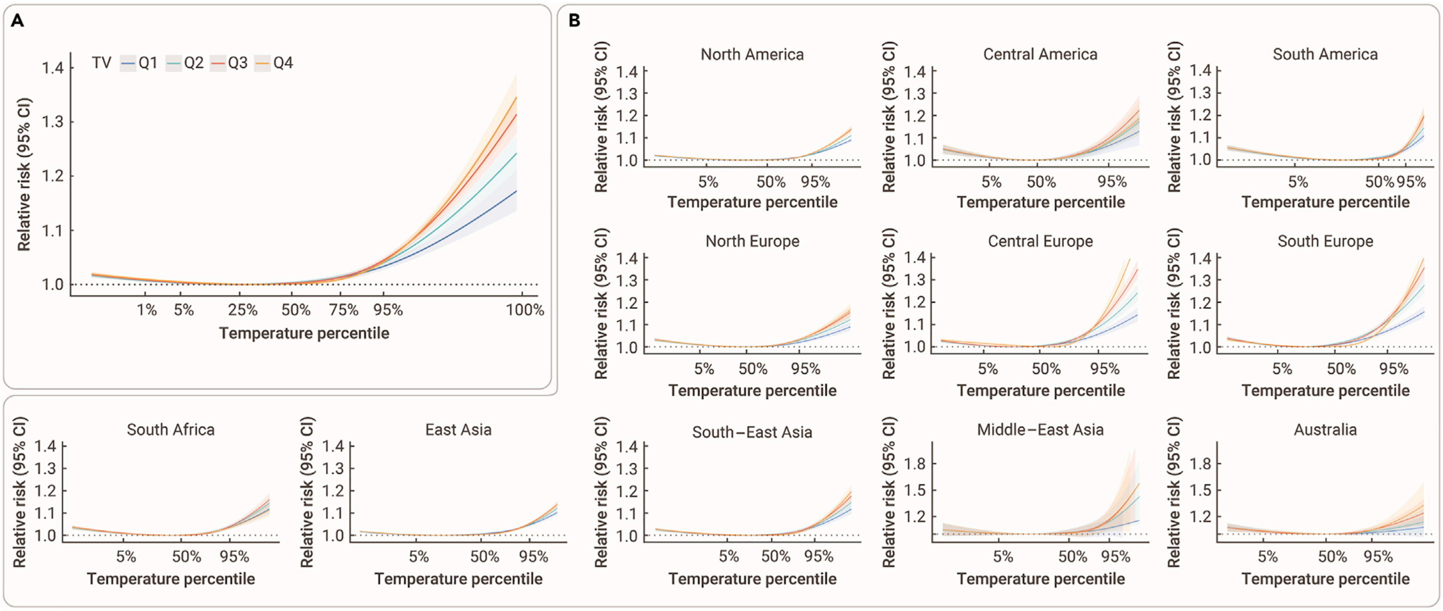

Figure 1. Overall cumulative exposure-response associations by temperature variability (A) Overall exposure-response curves between daily mean temperature and mortality in the warm season, stratified by quartiles of TV. (B) Regional exposure-response curves between daily mean temperature and daily mortality in the warm season, stratified by quartiles of TV. Shaded areas indicate the 95% CI. Definition of abbreviations:Q1 = the 1st quartile; Q2 = the 2nd quartile; Q3 = the 3rd quartile; Q4 = the 4th quartile; TV = temperature variability.

S9–S12) and after adding separate predictors in meta-regression with BLUP (Table S13). Using incremental lag periods, shortening the duration of the warm season, and adding relative humidity to the model, the modification effect of TV still existed (Tables S14–S16). The AFs changed slightly after using different methods to handle missing values (Table S17).

DISCUSSION

Our study showed that heat exposure together with high TV could significantly increase the mortality risk in the warm season. We saw an upward trend in premature death due to heat exposure with the increase in TV. For temperatures higher than TVST (96.34th percentile on average), AF showed a greater difference across 4 TV groups. The TV-modified heat-mortality burden showed disparate geographical variations.

The physiological mechanisms that explain the synergistic effects of TV and heat exposure on mortality are not yet clearly defined. However, there are several ways in which the two exposures may interact. When exposed to heat, people expend more of their reserves on thermoregulation to respond to heat.[19] This process involves elevations in heart rate and blood pressure, vasodilatation to transfer heat to the skin, and respiration to lose heat with the expired air.[20–22] Physiological adaptation to higher temperatures takes time. If the temperature suddenly changes in a short period of time, then people may have difficulty with internal thermoregulation, resulting in inflammatory responses and coagulation abnormalities induced by heat stress.[23] In addition, sudden temperature changes may also activate bronchopulmonary vagal afferent nerves and the inflammatory response.[24] For people with underlying conditions (e.g., preexisting illness, chronic diseases, poor fitness level), heat exposure may place extra pressure on the cardiovascular and respiratory systems,[25] especially when temperature changes dramatically in a short period. Consequently, heat-related deaths may occur.

Our findings are generally consistent with previous studies focusing on TV or heat, indicating that both heat exposure and TV were positively associated with mortality.[13,26,27] Some studies have explored the modification effect of temperature on the association between mortality and diurnal temperature range (DTR) or TV.[17,28–30] A study in Japan showed a higher risk of cardiovascular mortality associated with TV during extremely hot days in comparison with extremely cold days.[17] Another study in England and Wales showed a J-shaped curve of the relationship between percentiles of DTR and mortality in the warm season, while an inverted-V-shaped association was observed during the cold season.[28] Although many studies have quantified the mortality burden associated with heat exposure, to the best of our knowledge, none of them explored the potential heterogeneity attributable to TV.[31–33] Without taking TV into account when assessing the association between heat exposure and mortality, the heat-related mortality burden may be underestimated.

Some heterogeneity across regions was also found. For countries such as Guatemala and Colombia, temperatures above TVST together with low TV showed a higher mortality burden. The potential reasons for these results may derive from the low variation in TV and thus a small difference between Q1 and Q4 of TV. TV can be affected by many factors, such as greenness, soil moisture, and precipitation.[34,35] For example, vegetation removal and soil aridation would act to increase daily temperature fluctuation with more rainfall.[34] Temperate desert steppe generally has comparable warming effects on T_{min} and T_{max}, while temperate meadow and temperate steppe may have larger cooling effects on T_{max} than T_{min}.[35] In addition, population aging may mediate the association. The variation in heat exposure is caused by the differences in both vulnerable populations (in particular, the elderly) and temperature across regions.[36] For the countries such as South Africa (7.7%) and Cambodia (6.8%), the vulnerable population aged 60 or older accounted for only a small proportion of total population, lower than the global average level of 12.3% in 2015.[37] The lower the fraction of older persons, the less sensitivity to heat exposure for the whole country, and the lower the mortality burden attributable to heat exposure. With the deepening of the aging of society, vulnerable populations are expected to increase.[37] Further research is warranted to explore the geographic variation in TV-differentiated heat-related mortality burden and call for targeted strategies in mitigation and adaptation against climate change.

As a response to the increasing heat conditions, heat-health warning systems are developed in some parts of the world. For example, the WHO Regional Office for Europe developed the heat-health action plans (HHAPs) in 2008, covering 35 out of 53 member states of the WHO European Region by the end of 2018.[38] Although it is hard to assess how much heat-health prevention was associated with HHAPs, a substantial reduction in heat-related deaths was observed since the implementation of preventive measures (either HHAPs or other types of intervention).[38–41] However, the majority of warning systems use mean temperature or T_{max} as indices to trigger the warnings and inform the general public through the mass media without user-oriented attractive notification.[42,43] As suggested by our findings, the health effects of heat exposure could be magnified if they are accompanied by a higher TV and the findings were consistent across countries in Europe where HHAPs are implemented,[44] implying that it is difficult for current warning systems to obtain effective heat prevention when the TV is high.

Strengths and limitations

This study has several strengths. First, to the best of our knowledge, this is the first study to systematically explore the modifying effects of TV on heat-related

Table 2. Attributable fractions of mortality due to heat exposure, stratified by quantiles of TV in each region

	Attributable fraction (%)			
Region	Q1	Q2	Q3	Q4
North America	0.71 (−0.64 to 2.00)	1.07 (−0.52 to 2.56)	1.45 (−0.22 to 3.00)	1.74 (−0.09 to 3.39)
Central America	0.22 (−0.31 to 0.74)	0.42 (−0.46 to 1.25)	1.37 (−0.33 to 2.95)	2.19 (−0.25 to 4.44)
South America	0.82 (−0.75 to 2.32)	1.59 (−0.65 to 3.67)	2.21 (0.04–4.21)	2.98 (0.20–5.46)
Northern Europe	0.17 (−0.34 to 0.67)	0.44 (−0.49 to 1.32)	0.76 (−0.27 to 1.72)	2.52 (0.52–4.31)
Central Europe	0.26 (−0.30 to 0.80)	1.08 (−0.22 to 2.29)	2.41 (0.55–4.13)	7.68 (5.25–9.89)
Southern Europe	1.19 (0.07–2.24)	2.95 (0.99–4.76)	4.09 (1.76–6.23)	7.34 (4.17–10.20)
South Africa	0.44 (−0.85 to 1.68)	0.82 (−0.98 to 2.51)	1.22 (−1.06 to 3.32)	1.79 (−1.60 to 4.72)
Middle East Asia	1.65 (−1.76 to 4.77)	3.64 (−0.38 to 7.33)	4.23 (0.37–7.75)	3.73 (0.26–6.89)
East Asia	0.87 (0.03–1.66)	1.70 (0.41–2.92)	2.47 (0.97–3.87)	2.29 (0.94–3.54)
Southeast Asia	0.55 (−0.83 to 1.91)	1.16 (−1.68 to 3.87)	2.22 (−1.49 to 5.62)	3.56 (−0.87 to 7.36)
Australia	0.25 (−0.41 to 0.86)	0.69 (−0.33 to 1.64)	1.19 (−0.18 to 2.49)	2.62 (0.70–4.39)
International	0.70 (−0.33 to 1.69)	1.34 (−0.14 to 2.73)	1.99 (0.29–3.57)	2.73 (0.76–4.50)

Definition of abbreviations: Q1 = the 1st quartile; Q2 = the 2nd quartile; Q3 = the 3rd quartile; Q4 = the 4th quartile; TV = temperature variability.

mortality risks. The assessment of TV-differentiated mortality risks associated with heat exposure provides a better understanding of heat vulnerability. When quantifying the heat-related mortality burden, the effect of TV should not be ignored. Second, this study benefits from the large-scale investigation across multiple countries and a long period of time. It enables us to provide a global vision of the heat-related mortality burden under different TV exposure and to explore potential variation in estimations in terms of socioeconomic status and climatic and geographic features. Finally, our study targeted the heat-related mortality burden more precisely by highlighting the modification effect of TV when temperatures were higher than TVST.

Several limitations should be acknowledged. As the time series design and temperature data from fixed monitoring stations were used, ecological fallacy and measurement errors in exposure seem to be inevitable. Due to the lack of data for some areas of the world, some regions contain only one country. The extrapolation and interpretation of the findings are restricted to the generalization of our results. In addition, we are unable to investigate the modification effect of TV on the association between heat exposure and age- or cause-specific mortality. As the biological mechanisms are sensitive to causes of death, further research is warranted to investigate the disease-differentiated modification effect of TV on heat-related mortality burden.

CONCLUSIONS

Our findings, which are based on multi-country data, revealed that higher TV over a short period of time increased the mortality risks associated with heat exposure. It is imperative to raise public awareness of the potential health risks of TV. Targeted adaptation strategies against heat-related mortality burden should be implemented after taking into account the fluctuation of temperatures and geographical patterns.

MATERIALS AND METHODS

Data collection

The MCC Collaborative Research Network database (http://mccstudy.lshtm.ac.uk/) was used. Daily death counts and meteorological data, including mean temperature, maximum temperature (T_{max}), minimum temperature (T_{min}), and relative humidity were extracted. The *International Classification of Diseases*, 9th and 10th revisions (ICD-9 and ICD-10) codes were used to identify the causes of death. We extracted the data series on non-external causes of death (ICD-9: 0–799; ICD-10: A00–R99), or, if not available, all-cause mortality. Our analyses were restricted to locations with complete weather data in the warm season (the warmest 4 consecutive months) for at least 2 consecutive years. Detailed information on data cleaning is described in Text S1. Finally, 717 locations across 36 countries were included. Daily mortality, mean temperature, T_{min}, and T_{max} data had overall missing rates of 0.14%, 0.84%, 0.86%, and 0.73%, respectively (Table S1).

Calculation of temperature variability

TV was calculated as the standard deviation (SD) of the average of T_{min} and T_{max} for the current day and 1 day before ($T_{max\text{-}lag0}$, $T_{max\text{-}lag1}$, $T_{min\text{-}lag0}$, $T_{min\text{-}lag1}$).[13] In the sensitivity analysis, higher lengths of exposure (0–2 days and 0–3 days) were applied.

Statistical analysis

We used a two-stage time series design to assess the modification effect of TV on the heat-related mortality burden in the warm season. In the first stage, a generalized linear regression with the quasi-Poisson family allowing overdispersion in the death counts was applied for each location to obtain location-specific estimates for the heat-mortality association. To capture the modification effect of TV on the heat-mortality association, we introduced an interaction term between a cross-basis function of daily mean temperature and quartiles (Qs) of TV. We used relative rather than absolute levels of TV to accommodate different levels of adaptive capacity to TV across locations. The equation was as follows:

$$Y_{it} \sim \text{Poisson}(\mu;\theta)$$

$$\text{E}(Y_{it}) = \exp(\alpha_i + \beta_i TV_{it} + cb(\text{Temp}_{it}, \text{lag} = 10) + cb(\text{Temp}_{it}, \text{lag} = 10) \times \text{Quartile}_{TV} + ns(\text{Time}_{it}, df = 4\,/\,\text{year}) + \gamma_i DOW_{it})$$

$$\text{VAR}(Y_{it}) = \theta\mu$$

where Y_{it} is daily deaths counts in location i on day t; TV_{it} is the linear function of TV; and $cb(\text{Temp}_{it}, \text{lag} = 10)$, built by distributed-lag nonlinear models (DLNMs), is a cross-basis function of daily mean temperature featuring the nonlinear and delayed association over 10 days of lag.[45] It incorporates a natural cubic spline function with two internal knots placed at the 50th and 90th percentiles of the location-specific temperature distributions during the warm season and a natural cubic spline function of lag days with 2 internal knots placed at equally spaced values in the log scale to capture the delayed effect of temperature.[10] Quartile_{TV} stands for the dummy coded categorical variable of TV groups (from Q1 to Q4). To control for unmeasured temporal trends such as seasonality and long-term trend, $ns(\text{Time}_{it}, df = 4\,/\text{year})$, a natural cubic function of calendar days with 4 degrees of freedom (df) per year was included. In addition, an indicator for the day of the week (DOW_{it}) was adjusted in the model to control for weekly variations in risk.

In the second stage, multivariate random-effects meta-analysis without predictors was used to pool the location-specific estimates at the global, regional, and national levels. Precise estimates for each location were obtained using the best linear unbiased prediction (BLUP) estimations. BLUPs can provide more accurate estimates in locations with small daily death counts or short time series by borrowing information across locations.[46]

Quantification of temperature-specific risks and attributable fraction

For each quartile of TV, we showed the risk over a 10-day lag period at each temperature value compared with the risk at minimum mortality temperature (MMT), at which the

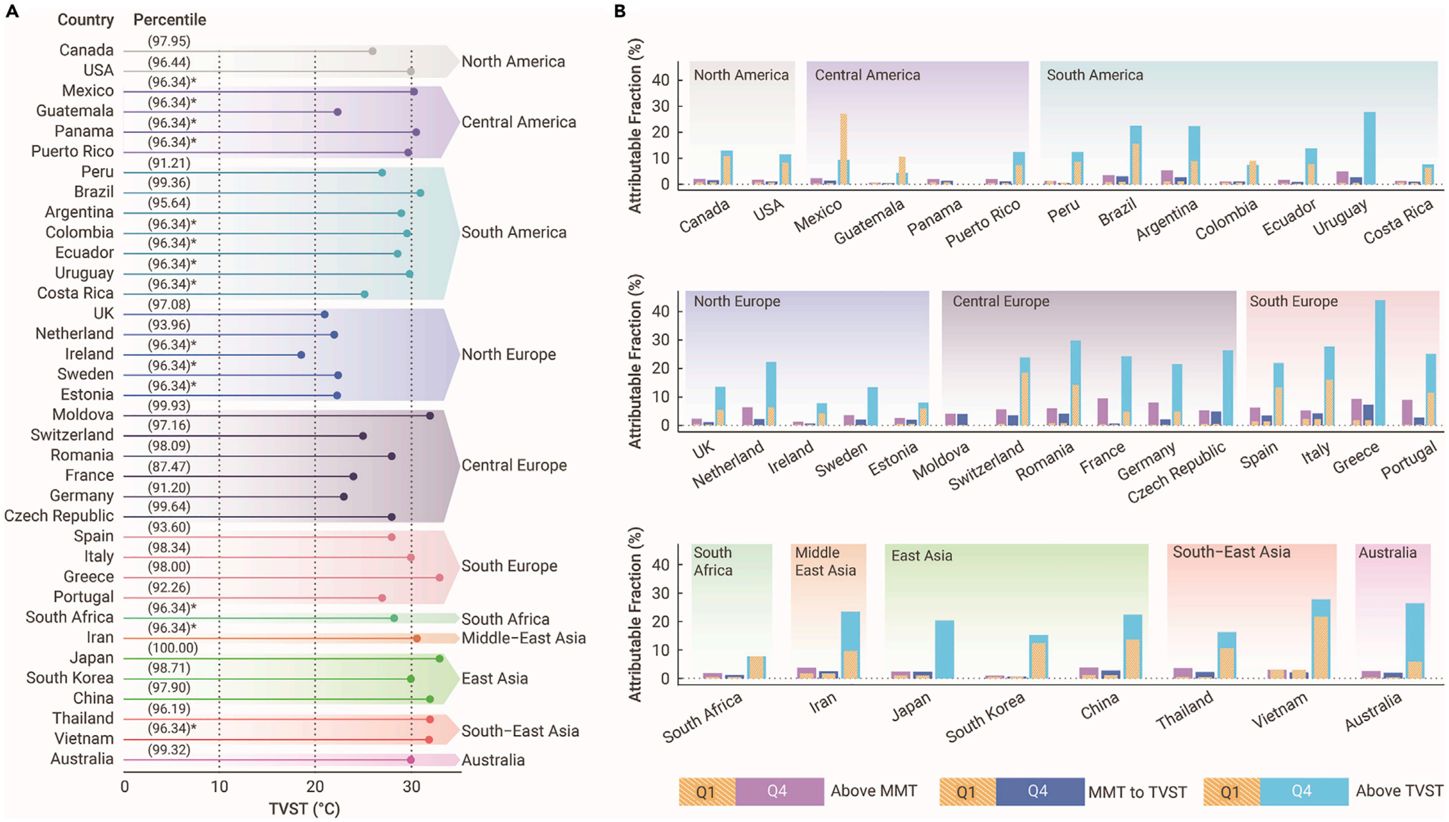

Figure 2. Figure 2 Fractions of all-cause mortality attributable to heat exposure by temperature variability (A) TVST identified for each country for TV. *For countries without identifiable TVST, the quantile threshold of the 96.34th percentile (the average of all identifiable country-specific TVSTs) in the temperature distribution for each country was used. (B) Comparison of AFs of mortality due to heat exposure for Q1 and Q4 of TV in each country, stratified by TVST. Yellow bars: the AFs for the Q1 of TV; purple bars: the AFs of mortality due to heat exposure above MMT for the Q4 of TV; dark blue bars: the AFs of mortality due to heat exposure from MMT to TVST for the Q4 of TV; light blue bars: the AFs of mortality due to heat exposure above TVST for the Q4 of TV. AF = attributable fraction; MMT = minimum mortality temperature; Q1 = the 1st quartile; Q4 = the 4th quartile; TV = temperature variability; TVST = temperature variability sensitive heat threshold.

risk of mortality was the lowest. The statistical significance of the difference in heat-mortality risks across quartiles of TV was assessed using repeated-measures multivariate meta-analysis. Briefly, based on location-specific estimates of four quartiles of TV, we performed a random effects meta-regression with TV quartiles as the only meta-predictor to test the difference in overall exposure-response curves of heat-mortality risks across quartiles of TV. Furthermore, in each country, to identify the potential targeted percentile ranges of temperature modified by TV, we tested the statistical significance of the difference in heat-mortality risks between Q1 and Q4 of TV using a fixed-effects meta-regression model at each temperature value. The modification effect of TV was identified as being statistically significant if there was a significant difference in heat-mortality risks between Q1 and Q4 of TV. The fixed-effects model was used because these country-specific estimates were based on the same samples. The country-specific temperature percentiles above which statistically significant modification effects of TV were observed, were called TV sensitive heat thresholds (TVSTs) and used to separate components of mortality burden attributable to heat exposure.

We compared the attributable deaths and attributable fractions (AFs) associated with heat exposure above MMT for each quartile of TV. Two components of heat exposure were used to separate the overall AF: from the quantile of MMT to TVST and above TVST. Daily attributable deaths due to each component of heat were calculated using BLUP location-specific association and then summed to obtain the total attributable deaths during the study period.[47] AF was computed by dividing the attributable deaths by the total death counts. Monte Carlo simulation (n = 1,000) was used to derive 95% CIs.

Sensitivity analysis

We conducted several sensitivity analyses to check the robustness of our results: (1) using different lengths of exposure to TV (TV 0–2 and TV 0–3); (2) adding separately location-specific predictors (region, average mean temperature, range of mean temperature, indicators for Köppen-Geiger climatic zones, gross domestic product [GDP] per capita, latitude, and longitude) to the meta-analytical model with BLUP in the second stage to check whether the pooled estimates would change while adjusting for the effects of each predictor on the location-specific estimates; (3) choosing alternative 2 and 3 warmest consecutive months to define the warm season; (4) changing the lag days of heat from 7 to 13 days; and (5) controlling the potential effect of relative humidity using a natural cubic spline with 3 df. Finally, although the overall missing rates for mortality and temperature data were generally small (Table S1), we also performed sensitivity analyses by restricting our analyses to locations with complete data and by using complete data after imputation. Missing values in time-series data were imputed by the spline interpolation method.

R software (version 3.6.2) with packages "dlnm" (for the construction of the cross-basis functions), "mvmeta" (for meta-regression), and "imputeTS" (for spline interpolation of the time-series data) was used to perform all of the analyses. A two-sided $p < 0.05$ was set as statistically significant. The code is available at the personal website of the first author (Github: https://github.com/yaowu-ops/Modification-effect-of-TV-on-heat-mortality-association.git).

Data sharing

Data were collected within the MCC Collaborative Research Network under a data-sharing agreement and cannot be made publicly available. Researchers can refer to MCC participants, who are listed as coauthors of this article, for information on accessing the data for each country.

REFERENCES

1. Cheng, H. (2020). Future earth and sustainable developments. The Innovation ***1***, 100055.
2. Zhou, T.J. (2021). New physical science behind climate change: what does IPCC AR6 tell us? The Innovation ***2***, 100173.
3. Watts, N., Amann, M., Arnell, N., et al. (2021). The 2020 report of the Lancet Countdown on health and climate change: responding to converging crises. Lancet ***397***, 129–170.
4. Kueh, M., Lin, C., Chuang, Y., et al. (2017). Climate variability of heat waves and their associated diurnal temperature range variations in Taiwan. Environ. Res. Lett. ***12***, 074017.
5. Christidis, N., Jones, G.S., and Stott, P.A. (2015). Dramatically increasing chance of extremely hot summers since the 2003 European heatwave. Nat. Clim. Change ***5***, 46–50.
6. Stocker, T.F., Qin, D., Plattner, G.-K., et al. (2014). Climate Change 2013: The Physical Science Basis. Working Group I Contribution to the Fifth Assessment Report (The Intergovernmental Panel on Climate Change) (Cambridge University Press).

7. Wellenius, G.A., Eliot, M.N., Bush, K.F., et al. (2017). Heat-related morbidity and mortality in New England: evidence for local policy. Environ. Res. ***156***, 845–853.
8. Murray, C.J., Aravkin, A.Y., Zheng, P., et al. (2020). Global burden of 87 risk factors in 204 countries and territories, 1990–2019: a systematic analysis for the Global Burden of Disease Study 2019. The Lancet ***396***, 1223–1249.
9. Zhao, Q., Guo, Y., Ye, T., et al. (2021). Global, regional, and national burden of mortality associated with non-optimal ambient temperatures from 2000 to 2019: a three-stage modelling study. Lancet Planet. Health ***5***, e415–e425.
10. Vicedo-Cabrera, A.M., Scovronick, N., Sera, F., et al. (2021). The burden of heat-related mortality attributable to recent human-induced climate change. Nat. Clim. Change ***11***, 492–500.
11. Bathiany, S., Dakos, V., Scheffer, M., and Lenton, T.M. (2018). Climate models predict increasing temperature variability in poor countries. Sci. Adv. ***4***, eaar5809.
12. Tamarin-Brodsky, T., Hodges, K., Hoskins, B.J., and Shepherd, T.G. (2020). Changes in Northern Hemisphere temperature variability shaped by regional warming patterns. Nat. Geosci. ***13***, 414–421.
13. Guo, Y., Gasparrini, A., Armstrong, B.G., et al. (2016). Temperature variability and mortality: a multi-country study. Environ. Health Perspect. ***124***, 1554–1559.
14. Zhao, Q., Coelho, M.S., Li, S., et al. (2018). Spatiotemporal and demographic variation in the association between temperature variability and hospitalizations in Brazil during 2000–2015: a nationwide time-series study. Environ. Int. ***120***, 345–353.
15. Shi, L., Kloog, I., Zanobetti, A., et al. (2015). Impacts of temperature and its variability on mortality in New England. Nat. Clim. Change ***5***, 988–991.
16. Deng, J., Hu, X., Xiao, C., et al. (2020). Ambient temperature and non-accidental mortality: a time series study. Environ. Sci. Pollut. Res. ***27***, 4190–4196.
17. Lee, W., Kim, Y., Honda, Y., and Kim, H. (2018). Association between diurnal temperature range and mortality modified by temperature in Japan, 1972–2015: investigation of spatial and temporal patterns for 12 cause-specific deaths. Environ. Int. ***119***, 379–387.
18. Zhang, Y., Yu, Y., Peng, M., et al. (2018). Temporal and seasonal variations of mortality burden associated with hourly temperature variability: a nationwide investigation in England and Wales. Environ. Int. ***115***, 325–333.
19. Nakamura, M., Yoda, T., Crawshaw, L.I., et al. (2008). Regional differences in temperature sensation and thermal comfort in humans. J. Appl. Physiol. ***105***, 1897–1906.
20. Ravanelli, N.M., Hodder, S.G., Havenith, G., and Jay, O. (2015). Heart rate and body temperature responses to extreme heat and humidity with and without electric fans. Jama ***313***, 724–725.
21. Madaniyazi, L., Zhou, Y., Li, S., et al. (2016). Outdoor temperature, heart rate and blood pressure in Chinese adults: effect modification by individual characteristics. Scientific Rep. ***6***, 21003.
22. Havenith, G. (2005). Temperature regulation, heat balance and climatic stress. In Extreme Weather Events and Public Health Responses, W. Kirch, R. Bertollini, and B. Menne, eds. (Springer Berlin Heidelberg), pp. 69–80.
23. Basu, R., Pearson, D., Malig, B., et al. (2012). The effect of high ambient temperature on emergency room visits. Epidemiology ***23***, 813–820.
24. Wu, Y., Xu, R., Wen, B., et al. (2021). Temperature variability and asthma hospitalisation in Brazil, 2000–2015: a nationwide case-crossover study. Thorax ***76***, 962–969.
25. Leon, L.R., and Helwig, B.G. (2010). Heat stroke: role of the systemic inflammatory response. J. Appl. Physiol. ***109***, 1980–1988.
26. Guo, Y., Gasparrini, A., Armstrong, B.G., et al. (2017). Heat wave and mortality: a multicountry, multicommunity study. Environ. Health Perspect. ***125***, 087006.
27. Liu, T., Zhou, C., Zhang, H., et al. (2021). Ambient temperature and years of life lost: a national study in China. The Innovation ***2***, 100072.
28. Zhang, Y., Peng, M., Wang, L., and Yu, C. (2018). Association of diurnal temperature range with daily mortality in England and Wales: a nationwide time-series study. Sci. Total Environ. ***619***, 291–300.
29. Sharafkhani, R., Khanjani, N., Bakhtiari, B., et al. (2017). Diurnal temperature range and mortality in Urmia, the Northwest of Iran. J. Therm. Biol. ***69***, 281–287.
30. Zhang, Y., Yu, C., Bao, J., and Li, X. (2017). Impact of temperature variation on mortality: an observational study from 12 counties across Hubei Province in China. Sci. Total Environ. ***587***, 196–203.
31. Gasparrini, A., Guo, Y., Hashizume, M., et al. (2015). Mortality risk attributable to high and low ambient temperature: a multicountry observational study. Lancet ***386***, 369–375.
32. Chen, R.J., Yin, P., Wang, L.J., et al. (2018). Association between ambient temperature and mortality risk and burden: time series study in 272 main Chinese cities. Br. Med. J. ***363***, k4306–k4317.
33. Son, J.-Y., Liu, J.C., and Bell, M.L. (2019). Temperature-related mortality: a systematic review and investigation of effect modifiers. Environ. Res. Lett. ***14***, 073004.
34. Zhou, L., Dickinson, R.E., Tian, Y., et al. (2007). Impact of vegetation removal and soil aridation on diurnal temperature range in a semiarid region: application to the Sahel. Proc. Natl. Acad. Sci.U S A ***104***, 17937–17942.
35. Shen, X.J., Liu, B.H., Zhou, D.W., and Lu, X.G. (2016). Effect of grassland vegetation on diurnal temperature range in China's temperate grassland region. Ecol. Eng. ***97***, 292–296.
36. Liu, Z., Anderson, B., Yan, K., et al. (2017). Global and regional changes in exposure to extreme heat and the relative contributions of climate and population change. Scientific Rep. ***7***, 43909.
37. Department of Economic and Social Affairs, Population Division (2015). World Population Ageing 2015 (United Nations).
38. Martinez, G.S., Linares, C., Ayuso, A., et al. (2019). Heat-health action plans in Europe: challenges ahead and how to tackle them. Environ. Res. ***176***, 108548.
39. Fouillet, A., Rey, G., Wagner, V., et al. (2008). Has the impact of heat waves on mortality changed in France since the European heat wave of summer 2003? A study of the 2006 heat wave. Int. J. Epidemiol. ***37***, 309–317.
40. De'Donato, F., Scortichini, M., De Sario, M., et al. (2018). Temporal variation in the effect of heat and the role of the Italian heat prevention plan. Public Health ***161***, 154–162.
41. Baccini, M., Kosatsky, T., Analitis, A., et al. (2011). Impact of heat on mortality in 15 European cities: attributable deaths under different weather scenarios. J. Epidemiol. Community Health ***65***, 64–70.
42. Casanueva, A., Burgstall, A., Kotlarski, S., et al. (2019). Overview of existing heat-health warning systems in Europe. Int. J. Environ. Res. Public Health ***16***, 2657.
43. Koppe, C., Sari Kovats, R., Jendritzky, G., and Menne, B. (2004). Heat-waves: Risks and Responses (World Health Organization).
44. European Climate and Health Observatory (2021). National heat-health warning systems and action plans. https://climate-adapt.eea.europa.eu/observatory/evidence/national-and-sub-national-warning-systems.
45. Gasparrini, A. (2011). Distributed lag linear and non-linear models in R: the package dlnm. J. Stat. Softw. ***43***, 1–20.
46. Gasparrini, A., Armstrong, B., and Kenward, M.G. (2012). Multivariate meta-analysis for non-linear and other multi-parameter associations. Stat. Med. ***31***, 3821–3839.
47. Gasparrini, A., and Leone, M. (2014). Attributable risk from distributed lag models. BMC Med. Res. Methodol. ***14***, 55–62.

ACKNOWLEDGMENTS

This study was supported by the Australian Research Council (DP210102076) and the Australian National Health and Medical Research Council (APP2000581). Y.W and B.W. were supported by the China Scholarship Council (nos. 202006010044 and 202006010043); S.L. was supported by an Emerging Leader Fellowship of the Australian National Health and Medical Research Council (no. APP2009866); Y.G. was supported by Career Development Fellowship (no. APP1163693) and Leader Fellowship (no. APP2008813) of the Australian National Health and Medical Research Council; J.K. and A.U. were supported by the Czech Science Foundation (project no. 20–28560S); N.S. was supported by the National Institute of Environmental Health Sciences-funded HERCULES Center (no. P30ES019776); Y.H. was supported by the Environment Research and Technology Development Fund (JPMEERF15S11412) of the Environmental Restoration and Conservation Agency; M.d.S.Z.S.C. and P.H.N.S. were supported by the São Paulo Research Foundation (FAPESP); H.O. and E.I. were supported by the Estonian Ministry of Education and Research (IUT34–17); J.M. was supported by a fellowship of Fundação para a Ciência e a Tecnlogia (SFRH/BPD/115112/2016); A.G. and F.S. were supported by the Medical Research Council UK (grant ID MR/R013349/1), the Natural Environment Research Council UK (grant ID NE/R009384/1), and the EU's Horizon 2020 project, Exhaustion (grant ID 820655); A.S. and F.d.D. were supported by the EU's Horizon 2020 project, Exhaustion (grant ID 820655); V.H. was supported by the Spanish Ministry of Economy, Industry and Competitiveness (grant ID PCIN-2017–046); and A.T. by MCIN/AEI/10.13039/501100011033 (grant CEX2018-000794-S). Statistics South Africa kindly provided the mortality data, but had no other role in the study.

AUTHOR CONTRIBUTIONS

Y.G., A.G., M.H., and B. Armstrong set up the collaborative network. Y.G., S.L., and Y.W. designed the study. Y.G., S.L., and A.G. developed the statistical methods. Y.W., B.W., S.L., and Y.G. took the lead in drafting the manuscript and interpreting the results. Y.W., B.W., Y.G., A.G., S.T., A.O., A.U., A.S., A.E., A.M.V.-C., A. Zanobetti, A.A., A. Zeka, A.T., B. Alahmad, B. Armstrong, B.F., C.Í., C. Ameling, C.D.I.C.V., C. Åström, D.H., D.V.D., D.R., E.I., E.L., F.M., F.A., F.D., F.S., G.C.-E., H. Kan, H.O., H. Kim, I.-H.H., J.K., J.M., J.S., K.K., M.H.-D., M.S.R., M.H., M.P., M.d.S.Z.S.C., N.S., P.M., P.G., P.H.N.S., R.A., S.O., T.N.D., V.C., V.H., W.L., X.S., Y.H., M.L.B., and S.L. provided the data and contributed to the interpretation of the results and the submitted version of the manuscript. Y.G., S.L., and Y.W. accessed and verified the data. All of the authors had full access to all of the data in the study and had final responsibility for the decision to submit for publication.

DECLARATION OF INTERESTS

The authors declare no competing interests.

SUPPLEMENTAL INFORMATION

Supplemental information can be found online at https://doi.org/10.1016/j.xinn.2022.100225.

LEAD CONTACT WEBSITES

https://research.monash.edu/en/persons/yuming-guo
https://research.monash.edu/en/persons/shandy-li

The Innovation, Volume *3*

Supplemental Information

Fluctuating temperature modifies heat-mortality association around the globe

Yao Wu, Bo Wen, Shanshan Li, Antonio Gasparrini, Shilu Tong, Ala Overcenco, Aleš Urban, Alexandra Schneider, Alireza Entezari, Ana Maria Vicedo-Cabrera, Antonella Zanobetti, Antonis Analitis, Ariana Zeka, Aurelio Tobias, Barrak Alahmad, Ben Armstrong, Bertil Forsberg, Carmen Íñiguez, Caroline Ameling, César De la Cruz Valencia, Christofer Åström, Danny Houthuijs, Do Van Dung, Dominic Royé, Ene Indermitte, Eric Lavigne, Fatemeh Mayvaneh, Fiorella Acquaotta, Francesca de'Donato, Francesco Sera, Gabriel Carrasco-Escobar, Haidong Kan, Hans Orru, Ho Kim, Iulian-Horia Holobaca, Jan Kyselý, Joana Madureira, Joel Schwartz, Klea Katsouyanni, Magali Hurtado-Diaz, Martina S. Ragettli, Masahiro Hashizume, Mathilde Pascal, Micheline de Sousa Zanotti Stagliorio Coélho, Noah Scovronick, Paola Michelozzi, Patrick Goodman, Paulo Hilario Nascimento Saldiva, Rosana Abrutzky, Samuel Osorio, Tran Ngoc Dang, Valentina Colistro, Veronika Huber, Whanhee Lee, Xerxes Seposo, Yasushi Honda, Michelle L. Bell, and Yuming Guo

Supplemental materials and methods

Table of contents

Text S1. Information about Data collection.

Figure S1. Country-specific exposure-response curve between mean temperature and mortality in warm season, stratified by quantiles of TV. Definition of abbreviations: TV = temperature variability; Q1 = the first quartile; Q2 = the second quartile; Q3 = the third quartile; Q4 = the fourth quartile.

Figure S2. Overall exposure-response curve between mean temperature and mortality in warm season, stratified by quantiles of TV 0–2. Definition of abbreviations: TV = temperature variability; Q1 = the first quartile; Q2 = the second quartile; Q3 = the third quartile; Q4 = the fourth quartile.

Figure S3. Overall exposure-response curve between mean temperature and mortality in warm season, stratified by quantiles of TV 0–3. Definition of abbreviations: TV = temperature variability; Q1 = the first quartile; Q2 = the second quartile; Q3 = the third quartile; Q4 = the fourth quartile.

Figure S4. Country-specific exposure-response curve between mean temperature and mortality in warm season, stratified by quantiles of TV 0–2. Definition of abbreviations: TV = temperature variability; Q1 = the first quartile; Q2 = the second quartile; Q3 = the third quartile; Q4 = the fourth quartile.

Figure S5. Country-specific exposure-response curve between mean temperature and mortality in warm season, stratified by quantiles of TV 0–3. Definition of abbreviations: TV = temperature variability; Q1 = the first quartile; Q2 = the second quartile; Q3 = the third quartile; Q4 = the fourth quartile.

Table S1. Descriptive information on missing rates of mortality and temperature data at the country level.

Table S2. Correlation coefficients between daily mean temperature and temperature variability.

Table S3. Description of TV and daily mean temperature in each quartile of TV for each location included in our study.

Table S4. Description of TV 0–2 and daily mean temperature in each quartile of TV 0–2 for each location included in our study.

Table S5. Description of TV 0–3 and daily mean temperature in each quartile of TV 0–3 for each location included in our study.

Table S6. Attributable deaths of mortality due to heat exposure, stratified by quantiles of TV in each region.

Table S7. Attributable fractions of mortality due to heat exposure stratified by quantiles of TV in each country.

Table S8. Attributable fractions of mortality due to heat exposure stratified by TVST for each quartile of TV in each country.

Table S9. Attributable fractions of mortality due to heat exposure stratified by quantiles of TV 0–2 in each country.

Table S10. Attributable fractions of mortality due heat exposure stratified by quantiles of TV 0–3 in each country.

Table S11. Attributable fractions of mortality due to heat exposure stratified by TVST for each quartile of TV 0–2 in each country.

Table S12. Attributable fractions of mortality due to heat exposure stratified by TVST for each quartile of TV 0–3 in each country.

Table S13. Overall attributable fractions after adding separate predictor in meta-regression with BLUP, stratified by quartiles of TV.

Table S14. Sensitivity analyses of overall attributable fractions after changing the lag of mean temperature in the main model, stratified by quartiles of TV.

Table S15. Sensitivity analyses of overall attributable fractions using different definitions of warm season, stratified by quartiles of TV.

Table S16. Sensitivity analyses of overall attributable fractions after adjusting relative humidity in the main model using 556 cities with relative humidity data, stratified by quartiles of TV.

Table S17. Sensitivity analyses of overall attributable fractions using different complete data after removing cities with missing vlaues or after imputaion, stratified by quartiles of TV.

Text S1. Information on data collection and data cleansing

We collected data on daily mortality and mean temperature (T_{mean}) in warm season for 750 cities across 43 countries or regions from the database of the MCC Collaborative Research Network (http://mccstudy.lshtm.ac.uk/). Detailed information on the process of data collection has been described in previous publications [1]. As daily minimum temperature (T_{min}) and maximum temperature (T_{max}), the primary exposures of interest in our study, were not available for some countries, we downloaded data from official websites instead. For European countries including U.K., Finland, Germany, Spain, we use the daily T_{mean}, T_{min}, and T_{max} data (10 km × 10 km resolution) extracted from the European daily high-resolution gridded dataset of surface temperature and precipitation (E-OBS) 23.1 gridded temperature datasets (https://surfobs.climate.copernicus.eu/dataaccess/access_eobs.php). E-OBS is a European land only daily high-resolution gridded dataset of maximum, mean, and minimum surface temperatures, precipitation amounts and sea level pressure, which ensured the uniformity of the temperature data in those countries. For the U.S., the daily T_{mean}, T_{min}, and T_{max} data were obtained from a national meteorological dataset (4 km × 4 km resolution) created by Parameter–Elevation Regressions on Independent Slopes Model (PRISM) (http://prism.oregonstate.edu/explorer/). The reliability of the above datasets has been proved in the previous study, ensuring a high level of consistency between them and observations from monitoring stations [2]. For China, we obtained data on daily T_{mean}, T_{min}, and T_{max} for each location included in our study from the China Meteorological Data Sharing Service Center. For locations with more than one monitoring station, daily meteorological data were calculated by averaging measurements from all valid monitoring stations in each location.

For each location, the following data cleansing procedures were used. First, we collected locations that have daily measurements of T_{mean}, T_{min}, T_{max}, and mortality for more than 99 days of warm season per year (corresponding to 80% of the complete data in warm season), and with a coverage over 2 years. For locations with several discontinuous periods over 2 years, we separated data into different datasets according to each period. Second, we identified the type of missing values for each location. As time-series data was used in this study, two types of missing manners were identified: the lack of measurements for certain variables and a small amount of missing values scattered during the study time period of each location. If it's unavailable of measurements for certain variable in one location, we excluded this location in the first step. For the second type of missing data, there is no suggestion that item being missing is correlated with death counts or any other predictors of interest. Previous studies using the same dataset

also suggested that measurements being missing were mainly caused by data logging errors or abnormal operations of the monitoring equipment [3]. Thus, they are more likely to go missing at random. A summary of missing rates of temperature data and mortality data for each country (**Table S1**). Total mortality, mean temperature, minimum temperature and maximum temperature data have an overall missing rate of 0.13%, 0.82%, 0.84%, and 0.71%, respectively. The total missing rates were relatively small, which has non-substantial influence on our estimates. To provide more convincing evidence, we also perform sensitivity analyses by restricting our analyses to locations with complete data and by using complete data after imputation.

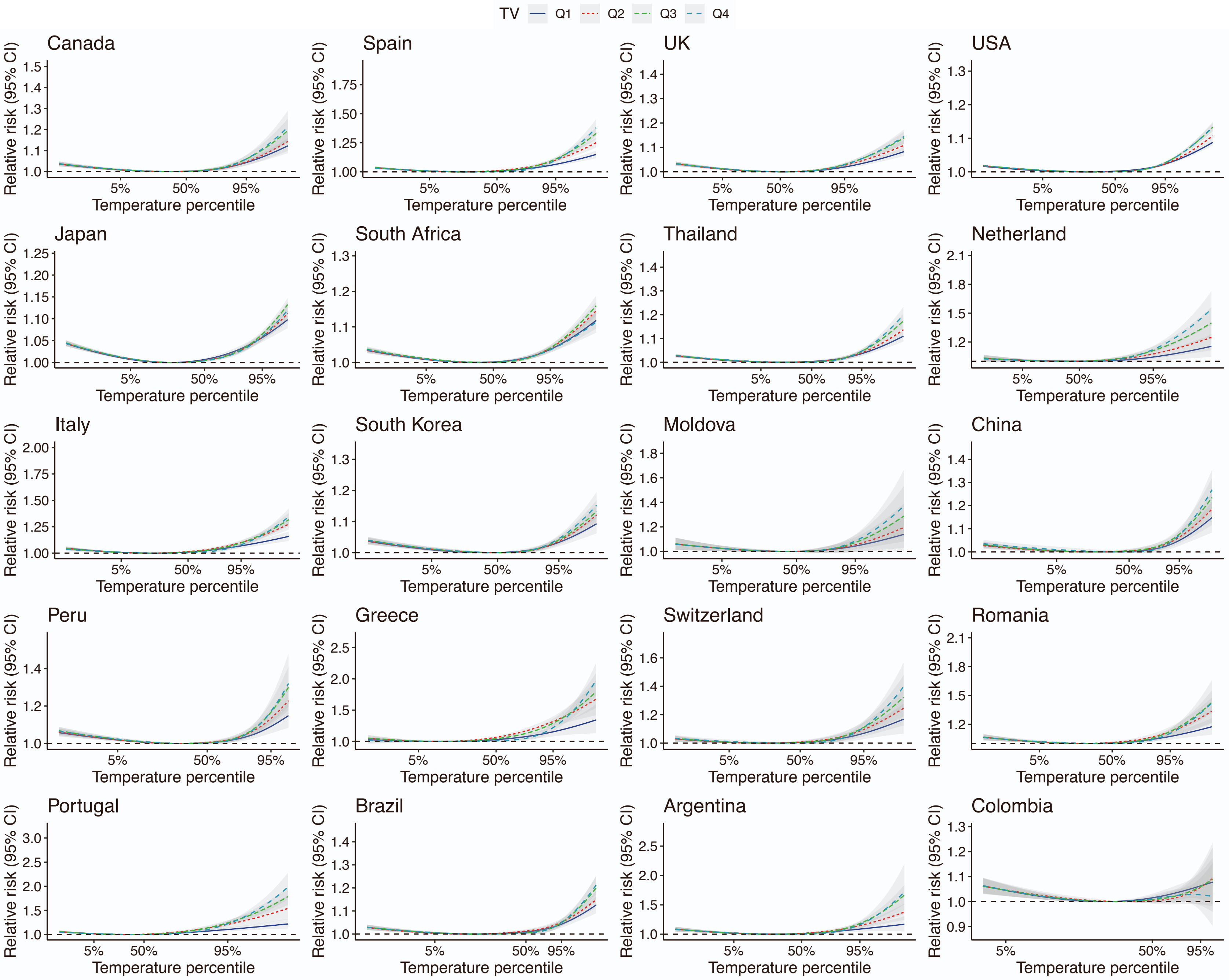
TV Q1 Q2 Q3 Q4
Canada
Spain
UK
USA
Japan
South Africa
Thailand
Netherland
Italy
South Korea
Moldova
China
Peru
Greece
Switzerland
Romania
Portugal
Brazil
Argentina
Colombia
Relative risk (95% CI)
Temperature percentile
5%
50%
95%

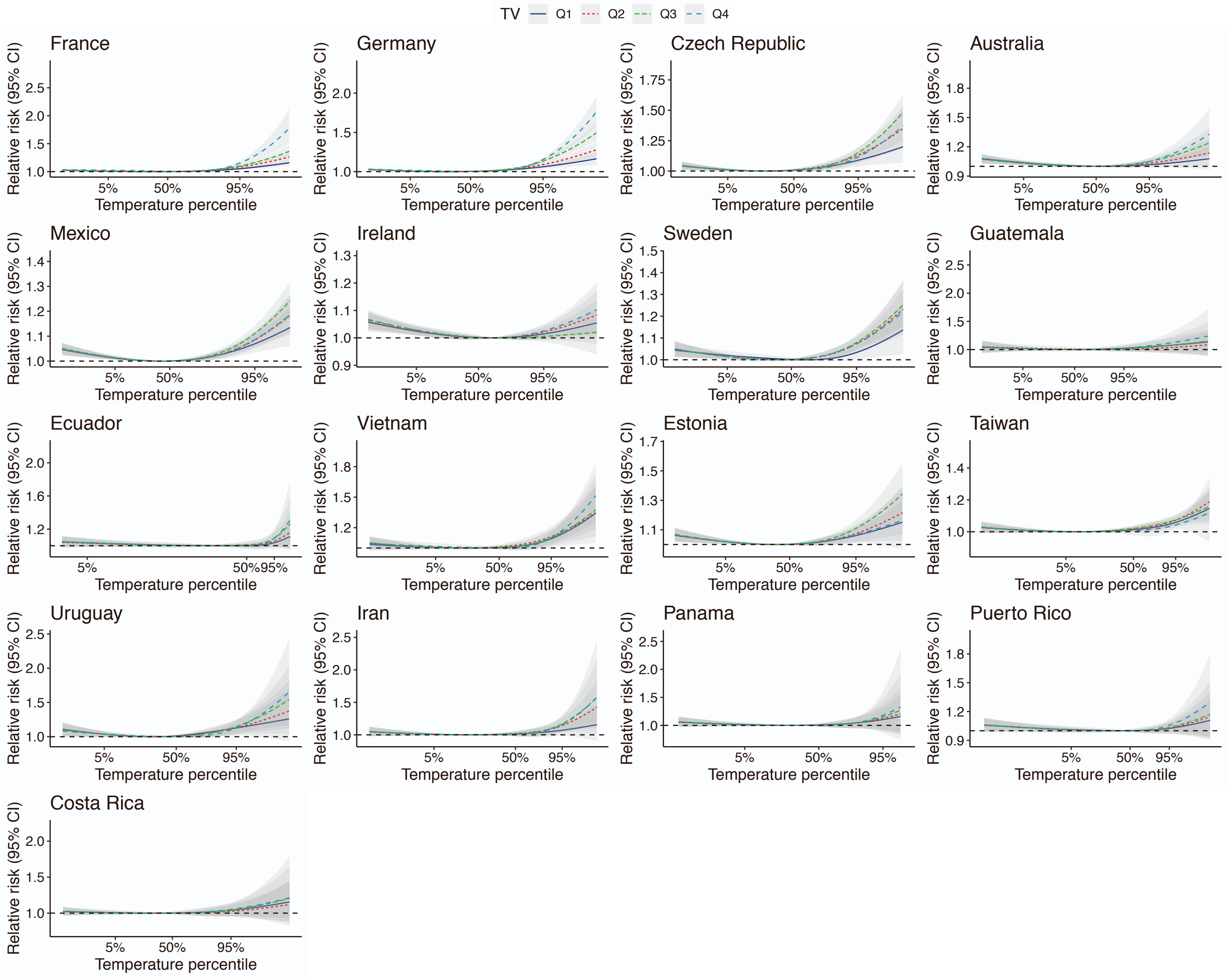

Figure S1. Country-specific exposure-response curve between mean temperature and mortality in warm season, stratified by quantiles of TV. Definition of abbreviations: TV = temperature variability; Q1 = the first quartile; Q2 = the second quartile; Q3 = the third quartile; Q4 = the fourth quartile.

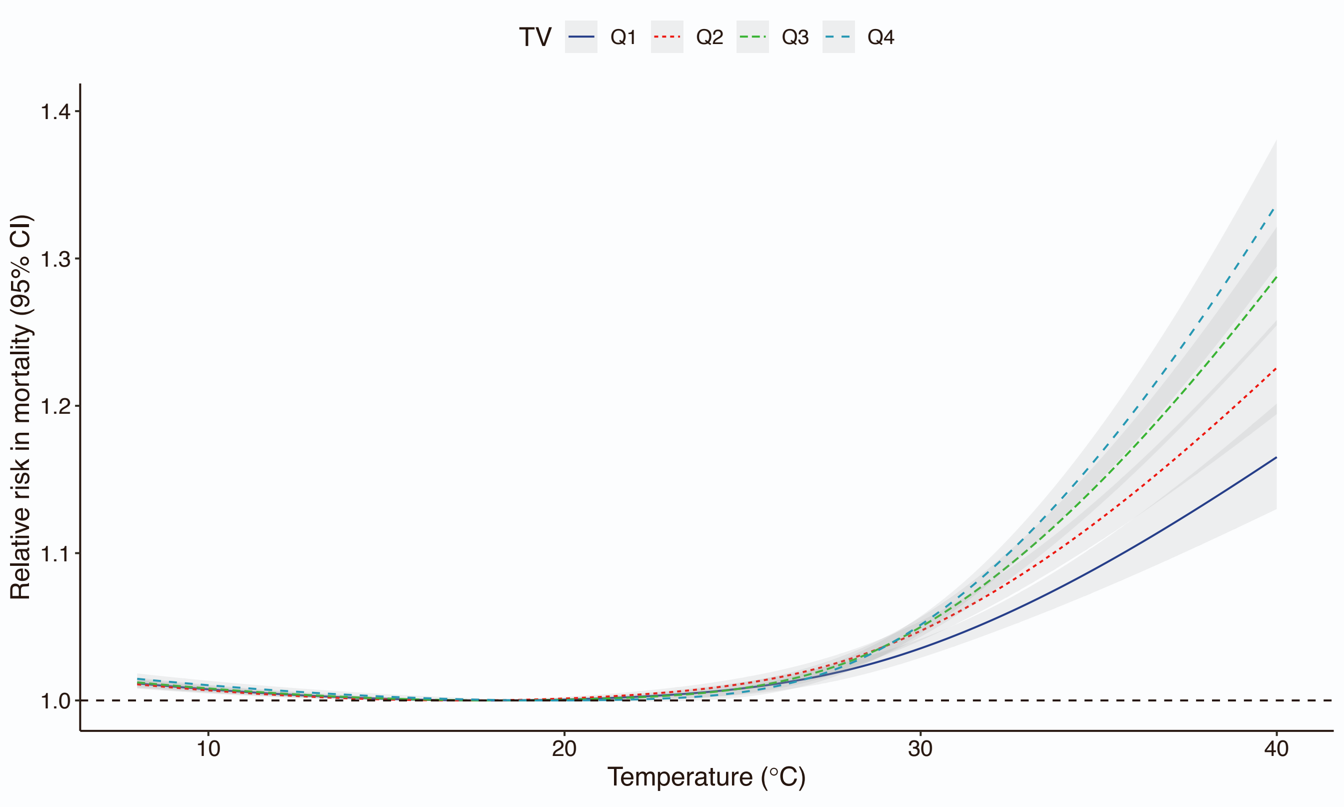

Figure S2. Overall exposure-response curve between mean temperature and mortality in warm season, stratified by quantiles of TV 0–2. Definition of abbreviations: TV = temperature variability; Q1 = the first quartile; Q2 = the second quartile; Q3 = the third quartile; Q4 = the fourth quartile.

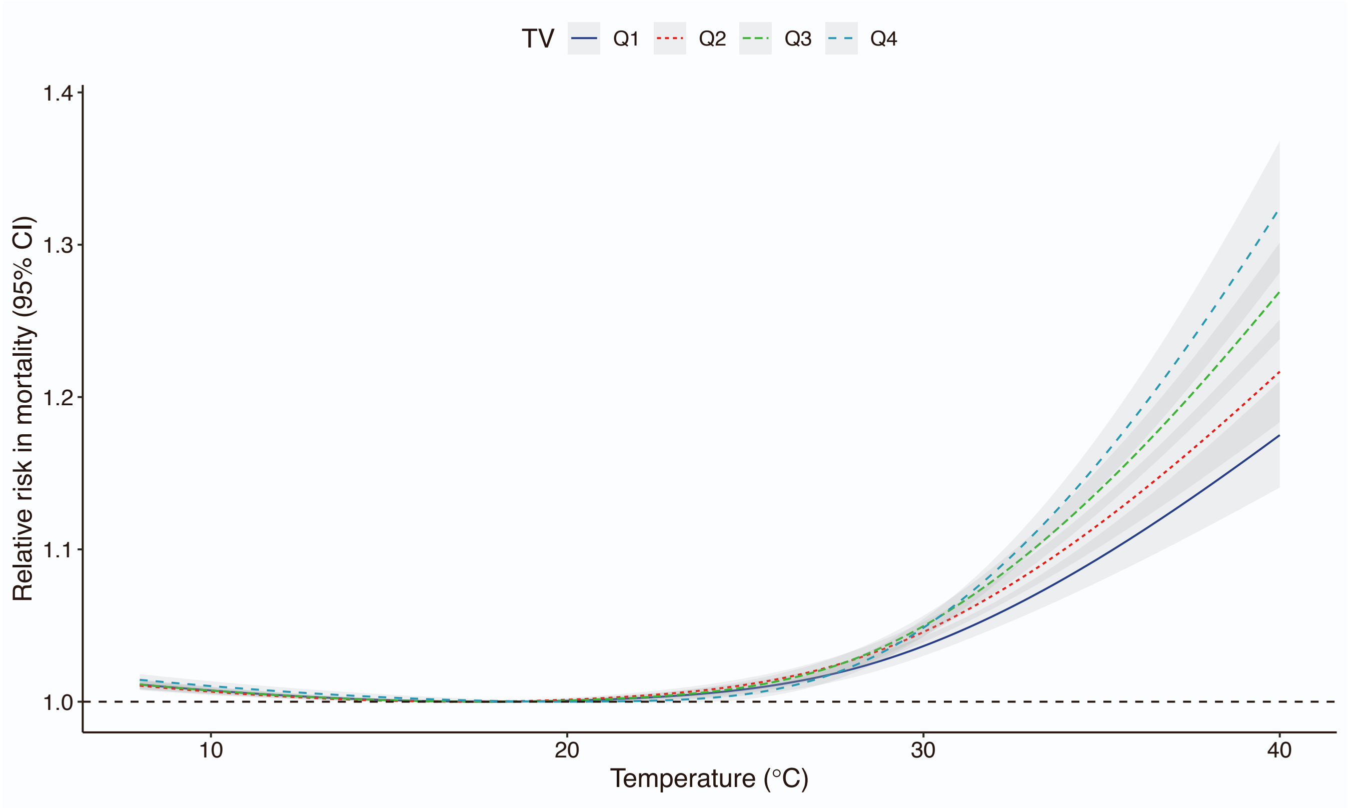

Figure S3. Overall exposure-response curve between mean temperature and mortality in warm season, stratified by quantiles of TV 0–3. Definition of abbreviations: TV = temperature variability; Q1 = the first quartile; Q2 = the second quartile; Q3 = the third quartile; Q4 = the fourth quartile.

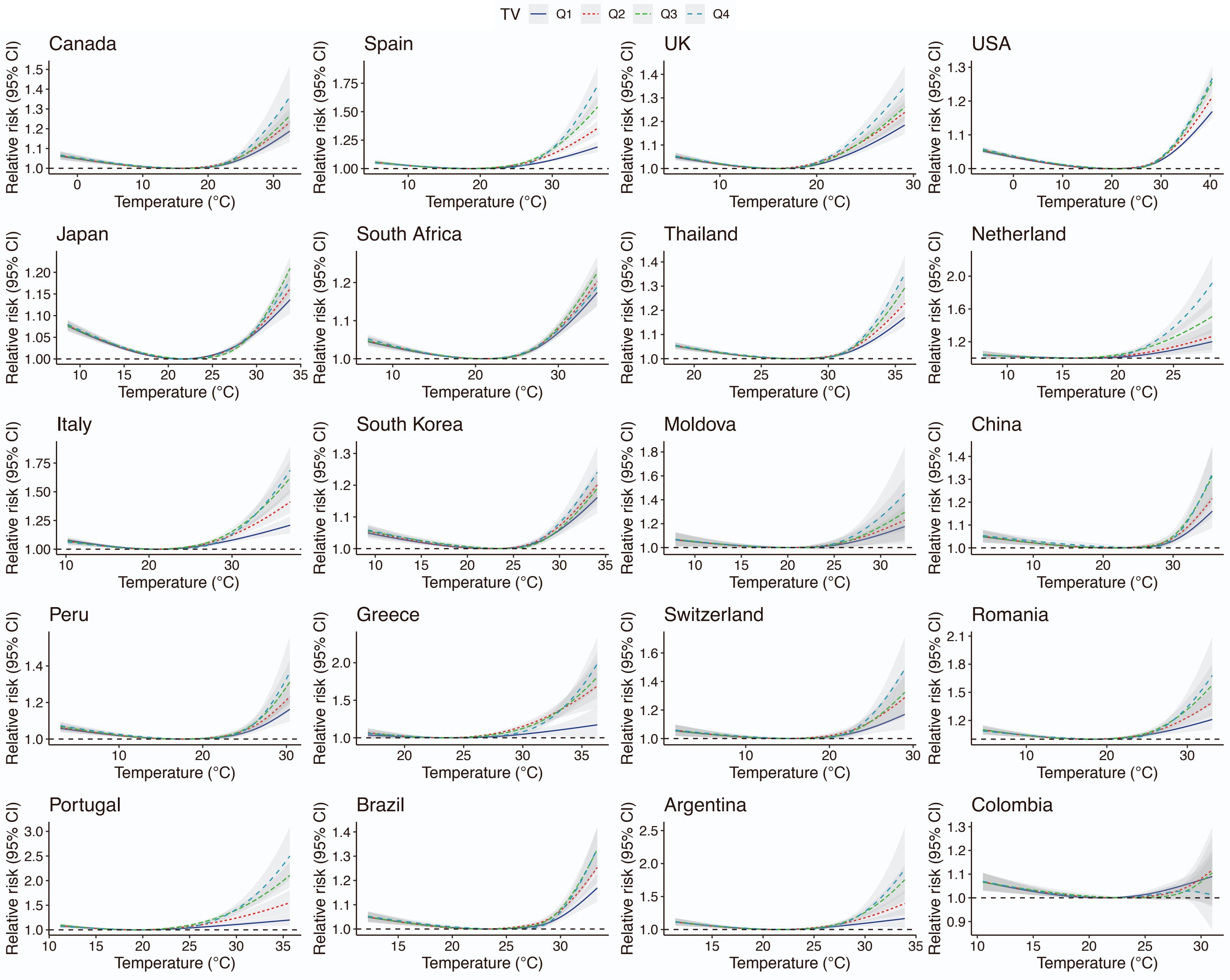
TV Q1 Q2 Q3 Q4
Canada
Spain
UK
USA
Japan
South Africa
Thailand
Netherland
Italy
South Korea
Moldova
China
Peru
Greece
Switzerland
Romania
Portugal
Brazil
Argentina
Colombia
Relative risk (95% CI)
Temperature (°C)

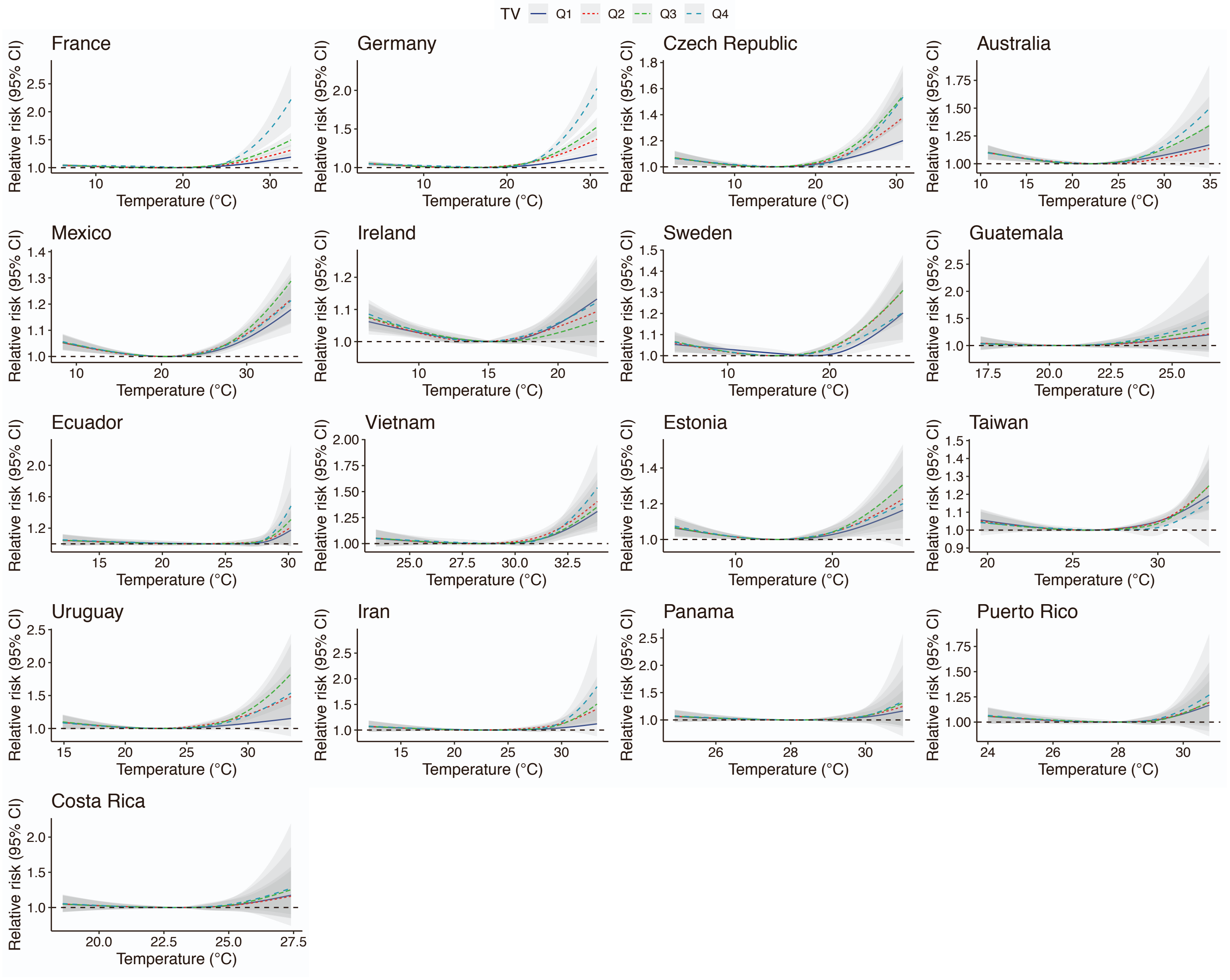

Figure S4. Country-specific exposure-response curve between mean temperature and mortality in warm season, stratified by quantiles of TV 0–2. Definition of abbreviations: TV = temperature variability; Q1 = the first quartile; Q2 = the second quartile; Q3 = the third quartile; Q4 = the fourth quartile.

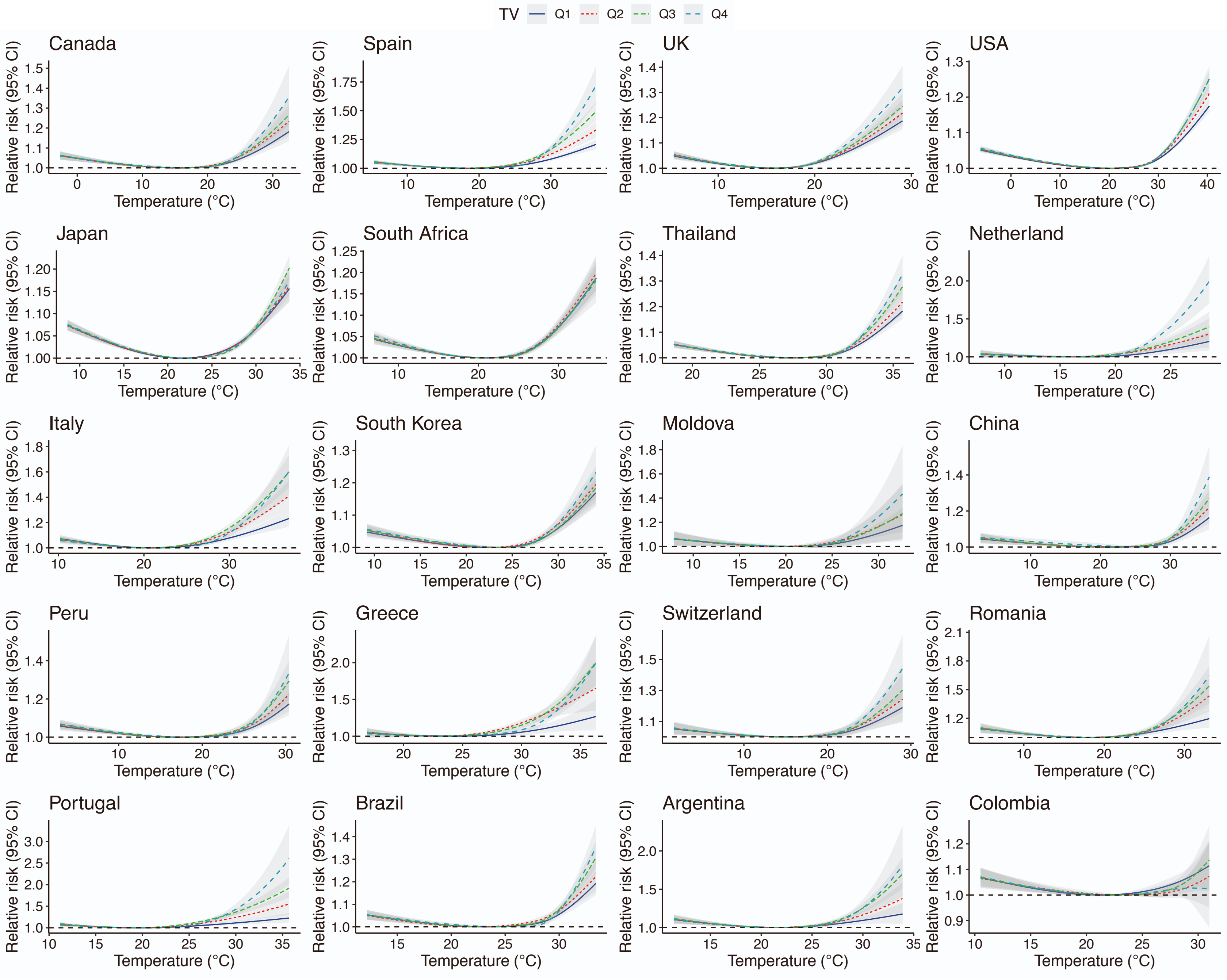
TV
Q1
Q2
Q3
Q4
Canada
Spain
UK
USA
Japan
South Africa
Thailand
Netherland
Italy
South Korea
Moldova
China
Peru
Greece
Switzerland
Romania
Portugal
Brazil
Argentina
Colombia
Relative risk (95% CI)
Temperature (°C)

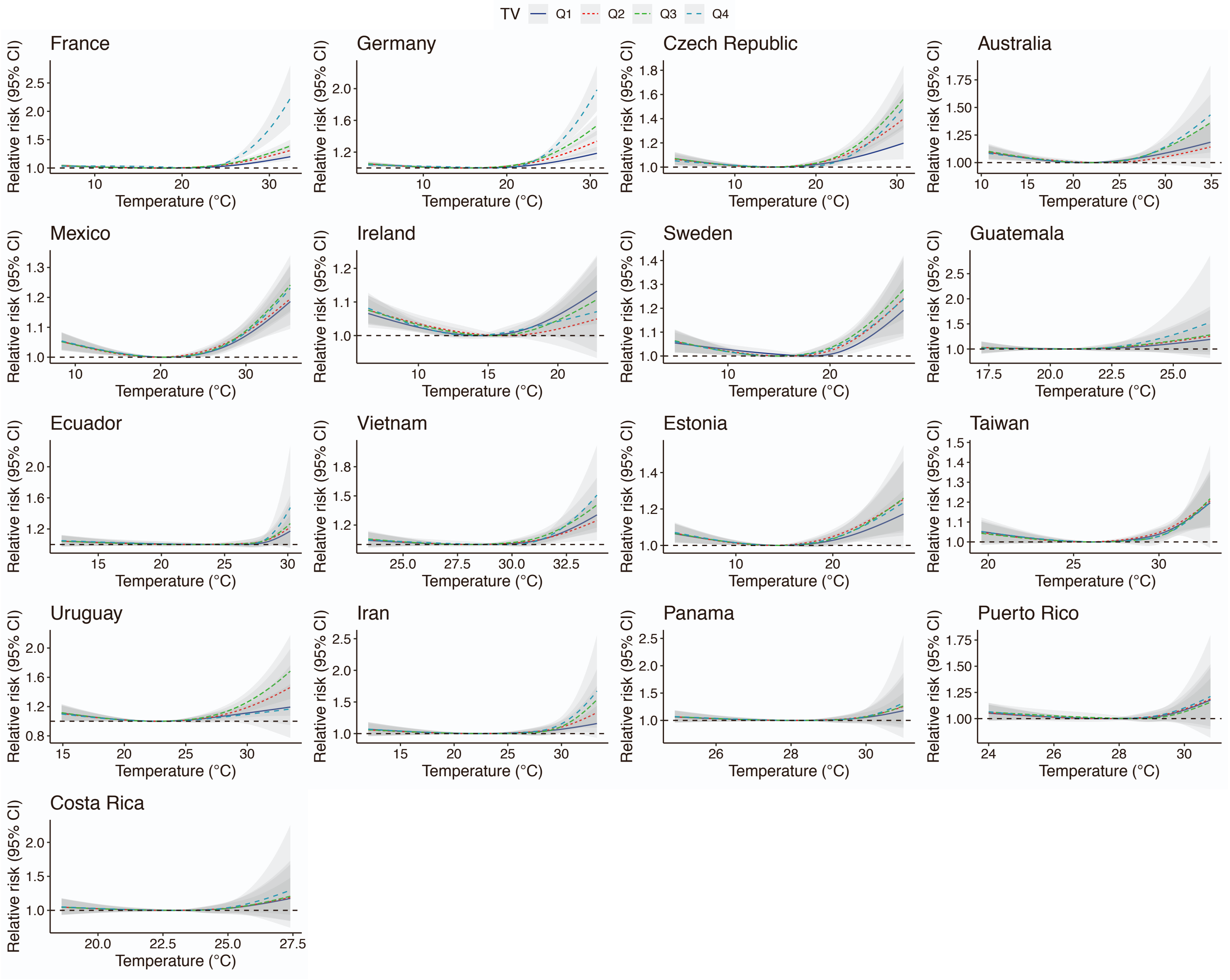

Figure S5. Country-specific exposure-response curve between mean temperature and mortality in warm season, stratified by quantiles of TV 0–3. Definition of abbreviations: TV = temperature variability; Q1 = the first quartile; Q2 = the second quartile; Q3 = the third quartile; Q4 = the fourth quartile.

Table S1. Descriptive information on missing rates of mortality and temperature data at the country level.

Country	Missing rates (%)			
	Minimum temperature	Mean temperature	Maximum temperature	Mortality
Argentina	0.05	0.00	0.00	0.95
Australia	0.00	0.00	0.00	0.31
Brazil	0.59	0.43	0.20	0.01
Canada	0.04	0.05	0.03	1.44
China	0.00	0.00	0.00	0.00
Colombia	2.41	2.09	1.16	0.00
Costa Rica	0.97	0.97	0.97	0.00
Czech Republic	0.00	0.00	0.00	0.00
Ecuador	2.66	2.66	2.66	0.00
Estonia	0.00	0.00	0.00	0.00
France	0.04	0.04	0.04	0.25
Germany	0.00	0.00	0.00	0.00
Greece	7.03	7.08	6.91	0.00
Guatemala	2.15	2.15	2.15	0.00
Iran	0.00	0.00	0.00	0.00
Ireland	0.00	0.00	0.00	0.01
Italy	1.08	0.03	0.19	0.01
Japan	0.01	0.02	0.01	0.00
Mexico	1.61	1.63	1.61	0.00
Moldova	0.00	0.00	0.00	0.00
Netherland	0.00	0.00	0.00	0.00
Panama	5.33	5.33	4.92	0.00
Peru	2.42	2.80	2.33	1.83
Portugal	0.25	0.25	0.25	0.12
Puerto Rico	1.78	2.05	0.65	0.00
Romania	0.00	0.00	0.00	0.00
South Africa	1.85	1.98	1.48	0.00
South Korea	0.00	0.02	0.00	0.00
Spain	0.00	0.00	0.00	0.00
Sweden	0.00	0.00	0.00	0.00
Switzerland	0.00	0.00	0.11	0.00
Thailand	0.07	0.07	0.04	0.00
UK	0.00	0.00	0.00	0.00
Uruguay	0.00	0.00	0.00	0.00
USA	0.00	0.00	0.00	0.00
Vietnam	0.57	0.57	0.57	0.00
Pooled	0.86	0.84	0.73	0.14

Table S2. Correlation coefficients between daily mean temperature and temperature variability.

Country	TV 0–1		TV 0–2		TV 0–3	
	Correlation coefficient	P value	Correlation coefficient	P value	Correlation coefficient	P value
Global	0.10	<0.001	0.10	<0.001	0.10	<0.001
Argentina	0.25	<0.001	0.25	<0.001	0.25	<0.001
Australia	0.27	<0.001	0.27	<0.001	0.27	<0.001
Brazil	0.08	<0.001	0.08	<0.001	0.08	<0.001
Canada	0.08	<0.001	0.08	<0.001	0.08	<0.001
China	-0.29	<0.001	-0.29	<0.001	-0.29	<0.001
Colombia	-0.31	<0.001	-0.31	<0.001	-0.31	<0.001
Costa Rica	0.21	<0.001	0.21	<0.001	0.21	<0.001
Czech Republic	0.52	<0.001	0.52	<0.001	0.52	<0.001
Ecuador	-0.58	<0.001	-0.58	<0.001	-0.58	<0.001
Estonia	0.22	<0.001	0.22	<0.001	0.22	<0.001
France	0.30	<0.001	0.30	<0.001	0.30	<0.001
Germany	0.53	<0.001	0.53	<0.001	0.53	<0.001
Greece	0.42	<0.001	0.42	<0.001	0.42	<0.001
Guatemala	0.35	<0.001	0.35	<0.001	0.35	<0.001
Iran	0.04	0.21	0.04	0.21	0.04	0.21
Ireland	0.05	<0.001	0.05	<0.001	0.05	<0.001
Italy	0.22	<0.001	0.22	<0.001	0.22	<0.001
Japan	0.14	<0.001	0.14	<0.001	0.14	<0.001
Mexico	0.10	<0.001	0.10	<0.001	0.10	<0.001
Moldova	0.32	<0.001	0.32	<0.001	0.32	<0.001
Netherland	0.39	<0.001	0.39	<0.001	0.39	<0.001
Panama	0.07	0.31	0.07	0.31	0.07	0.31
Peru	-0.17	<0.001	-0.17	<0.001	-0.17	<0.001
Portugal	0.58	<0.001	0.58	<0.001	0.58	<0.001
Puerto Rico	0.16	<0.001	0.16	<0.001	0.16	<0.001
Romania	0.27	<0.001	0.27	<0.001	0.27	<0.001
South Africa	0.32	<0.001	0.32	<0.001	0.32	<0.001
South Korea	-0.01	<0.001	-0.01	<0.001	-0.01	<0.001
Spain	0.26	<0.001	0.26	<0.001	0.26	<0.001
Sweden	0.39	<0.001	0.39	<0.001	0.39	<0.001
Switzerland	0.47	<0.001	0.47	<0.001	0.47	<0.001
Thailand	0.38	<0.001	0.38	<0.001	0.38	<0.001
UK	0.07	<0.001	0.07	<0.001	0.07	<0.001
Uruguay	0.54	<0.001	0.54	<0.001	0.54	<0.001
USA	-0.02	<0.001	-0.02	<0.001	-0.02	<0.001
Vietnam	0.51	<0.001	0.51	<0.001	0.51	<0.001

Table S6. Attributable deaths of mortality due to heat exposure, stratified by quantiles of TV in each region.

Region	Attributable deaths (thousand)			
	Q1	Q2	Q3	Q4
North America	19.84 (-17.71 to 55.61)	29.76 (-14.30 to 71.17)	40.21 (-6.13 to 83.16)	48.31 (-2.45 to 93.97)
Central America	0.43 (-0.60 to 1.41)	0.81 (-0.89 to 2.43)	2.69 (-0.65 to 5.82)	4.39 (-0.49 to 8.90)
South America	3.64 (-3.33 to 10.28)	7.15 (-2.94 to 16.52)	10.10 (0.18 to 19.20)	13.65 (0.90 to 24.98)
North Europe	1.08 (-2.11 to 4.19)	2.71 (-3.04 to 8.17)	4.74 (-1.66 to 10.71)	16.19 (3.35 to 27.65)
Central Europe	1.35 (-1.51 to 4.12)	5.57 (-1.14 to 11.84)	12.69 (2.89 to 21.82)	42.31 (28.92 to 54.50)
South Europe	4.79 (0.29 to 9.01)	12.07 (4.06 to 19.45)	16.96 (7.30 to 25.84)	31.38 (17.81 to 43.62)
South Africa	2.25 (-4.30 to 8.52)	4.31 (-5.12 to 13.15)	6.49 (-5.66 to 17.66)	9.77 (-8.72 to 25.81)
Middle-East Asia	0.16 (-0.17 to 0.46)	0.37 (-0.04 to 0.74)	0.44 (0.04 to 0.81)	0.40 (0.03 to 0.73)
East Asia	28.43 (1.04 to 54.09)	56.67 (13.58 to 97.00)	82.96 (32.71 to 129.98)	77.09 (31.52 to 119.04)
South-East Asia	0.81 (-1.23 to 2.85)	1.76 (-2.54 to 5.87)	3.38 (-2.26 to 8.56)	5.51 (-1.34 to 11.40)
Australia	0.22 (-0.35 to 0.75)	0.59 (-0.29 to 1.42)	1.03 (-0.15 to 2.16)	2.31 (0.62 to 3.86)
International	63.01 (-29.99 to 151.28)	121.77 (-12.66 to 247.77)	181.71 (26.61 to 325.73)	251.30 (70.14 to 414.47)

Table S7. Attributable fractions of mortality due to heat exposure stratified by quantiles of TV in each country.

Country	Q1 of TV	Q2 of TV	Q3 of TV	Q4 of TV
Argentina	1.05 (-0.48 to 2.50)	2.94 (0.69 to 5.06)	5.32 (3.05 to 7.45)	4.41 (2.14 to 6.49)
Australia	0.25 (-0.41 to 0.86)	0.69 (-0.33 to 1.64)	1.19 (-0.18 to 2.49)	2.62 (0.70 to 4.39)
Brazil	0.85 (-0.69 to 2.32)	1.61 (-0.72 to 3.78)	2.07 (-0.11 to 4.06)	3.38 (0.34 to 6.08)
Canada	0.65 (-0.48 to 1.72)	1.24 (-0.23 to 2.61)	1.62 (-0.10 to 3.21)	2.08 (-0.10 to 4.02)
China	1.27 (-0.66 to 3.10)	1.80 (-1.20 to 4.61)	3.03 (-0.35 to 6.11)	3.57 (0.08 to 6.44)
Colombia	0.51 (-0.81 to 1.76)	0.51 (-0.62 to 1.56)	0.72 (-0.79 to 2.13)	1.39 (-1.17 to 3.63)
Costa Rica	0.59 (-1.37 to 2.55)	0.38 (-1.29 to 1.89)	0.92 (-1.58 to 3.29)	1.32 (-1.83 to 4.23)
Czech Republic	0.34 (-0.37 to 0.99)	1.45 (-0.09 to 2.87)	3.63 (1.11 to 5.92)	5.24 (2.26 to 7.99)
Ecuador	0.26 (-0.77 to 1.24)	1.02 (-1.96 to 3.74)	1.30 (-1.49 to 3.87)	1.67 (-0.74 to 3.93)
Estonia	0.35 (-0.81 to 1.51)	0.92 (-0.83 to 2.59)	2.28 (-0.42 to 4.78)	2.59 (-1.61 to 6.35)
France	0.23 (-0.29 to 0.74)	0.63 (-0.48 to 1.68)	1.63 (0.08 to 3.09)	9.49 (7.39 to 11.36)
Germany	0.13 (-0.27 to 0.52)	0.59 (-0.45 to 1.57)	2.20 (0.58 to 3.71)	8.02 (5.74 to 10.12)
Greece	1.65 (0.09 to 3.10)	6.23 (3.89 to 8.33)	7.80 (4.98 to 10.25)	9.27 (5.28 to 12.73)
Guatemala	0.29 (-0.36 to 0.91)	0.08 (-0.24 to 0.35)	0.46 (-0.95 to 1.81)	0.59 (-1.30 to 2.40)
Iran	1.65 (-1.76 to 4.77)	3.64 (-0.38 to 7.33)	4.23 (0.37 to 7.75)	3.73 (0.26 to 6.89)
Ireland	0.22 (-0.48 to 0.88)	0.51 (-0.47 to 1.41)	0.16 (-0.28 to 0.59)	1.25 (-0.57 to 2.82)
Italy	2.21 (0.18 to 4.15)	4.22 (1.35 to 6.92)	4.37 (1.50 to 7.02)	5.15 (2.22 to 7.79)
Japan	0.90 (0.10 to 1.64)	1.76 (0.52 to 2.92)	2.55 (1.12 to 3.89)	2.37 (1.05 to 3.59)
Mexico	0.22 (-0.29 to 0.71)	0.42 (-0.45 to 1.25)	1.39 (-0.29 to 2.96)	2.23 (-0.20 to 4.48)
Moldova	0.08 (-0.38 to 0.56)	0.35 (-0.97 to 1.67)	1.15 (-0.79 to 3.02)	4.12 (0.72 to 7.17)
Netherland	0.10 (-0.47 to 0.66)	0.46 (-0.83 to 1.69)	1.55 (-0.33 to 3.31)	6.35 (2.83 to 9.44)
Panama	0.66 (-1.59 to 2.94)	3.77 (-4.10 to 10.02)	5.15 (-5.97 to 13.42)	2.04 (-1.80 to 5.23)
Peru	1.12 (-1.47 to 3.56)	1.83 (-1.70 to 5.02)	1.66 (-1.02 to 4.08)	1.26 (-0.87 to 3.07)
Portugal	0.21 (-0.15 to 0.56)	1.29 (0.48 to 2.06)	2.73 (1.48 to 3.87)	9.12 (6.80 to 11.19)
Puerto Rico	0.35 (-2.01 to 2.76)	0.37 (-1.14 to 1.74)	0.54 (-1.54 to 2.45)	2.03 (-1.48 to 5.17)
Romania	0.70 (-0.25 to 1.63)	3.16 (1.11 to 5.01)	3.46 (1.23 to 5.56)	5.96 (3.58 to 8.17)
South Africa	0.44 (-0.85 to 1.68)	0.82 (-0.98 to 2.51)	1.22 (-1.06 to 3.32)	1.79 (-1.60 to 4.72)
South Korea	0.44 (-0.63 to 1.49)	1.01 (-0.46 to 2.40)	1.33 (-0.46 to 2.99)	0.97 (-0.18 to 2.00)
Spain	1.44 (0.17 to 2.61)	3.23 (0.91 to 5.35)	4.46 (1.70 to 7.01)	6.69 (3.00 to 10.05)
Sweden	0.06 (-0.17 to 0.27)	0.43 (-0.43 to 1.27)	1.29 (-0.16 to 2.61)	3.35 (0.75 to 5.74)
Switzerland	0.33 (-0.57 to 1.23)	1.18 (-1.08 to 3.30)	2.62 (-0.83 to 5.83)	5.64 (0.72 to 9.92)
Thailand	0.39 (-0.89 to 1.67)	0.86 (-1.93 to 3.52)	2.11 (-1.62 to 5.51)	3.59 (-0.95 to 7.50)
UK	0.18 (-0.31 to 0.66)	0.41 (-0.47 to 1.25)	0.70 (-0.27 to 1.61)	2.33 (0.55 to 3.93)
Uruguay	0.50 (-0.13 to 1.10)	1.91 (-0.11 to 3.84)	4.17 (1.02 to 7.10)	4.94 (1.68 to 7.94)
USA	0.72 (-0.65 to 2.03)	1.05 (-0.55 to 2.56)	1.43 (-0.23 to 2.98)	1.70 (-0.09 to 3.32)
Vietnam	2.87 (0.11 to 5.60)	5.63 (2.03 to 9.04)	3.90 (0.52 to 7.27)	2.99 (0.48 to 5.21)
Total	0.70 (-0.33 to 1.69)	1.34 (-0.14 to 2.73)	1.99 (0.29 to 3.57)	2.73 (0.76 to 4.50)

Definition of abbreviations: TV = temperature variability; Q1 = the first quartile; Q2 = the second quartile; Q3 = the third quartile; Q4 = the fourth quartile.

Table S8. Attributable fractions of mortality due to heat exposure stratified by TVST for each quartile of TV in each country.

Country	Attributable fractions (%): MMT–TVST				Attributable fractions (%): above TVST			
	Q1 of TV	Q2 of TV	Q3 of TV	Q4 of TV	Q1 of TV	Q2 of TV	Q3 of TV	Q4 of TV
Argentina	1.02 (-0.49 to 2.45)	2.62 (0.42 to 4.69)	3.60 (1.48 to 5.57)	2.71 (0.66 to 4.67)	8.90 (-1.13 to 18.06)	18.01 (6.98 to 27.26)	29.16 (21.63 to 36.17)	22.33 (13.29 to 29.75)
Australia	0.25 (-0.40 to 0.85)	0.68 (-0.33 to 1.63)	1.16 (-0.19 to 2.45)	2.01 (0.33 to 3.58)	5.75 (-10.36 to 16.87)	8.90 (-3.30 to 18.97)	15.69 (0.26 to 27.67)	26.46 (4.71 to 41.51)
Brazil	0.82 (-0.71 to 2.28)	1.57 (-0.74 to 3.71)	1.95 (-0.19 to 3.92)	3.04 (0.08 to 5.71)	15.44 (-7.66 to 32.03)	12.65 (-6.28 to 26.59)	20.64 (4.99 to 31.93)	22.48 (3.06 to 35.91)
Canada	0.62 (-0.50 to 1.67)	1.09 (-0.32 to 2.42)	1.31 (-0.28 to 2.80)	1.52 (-0.33 to 3.23)	10.74 (-1.00 to 20.62)	13.68 (2.70 to 22.85)	13.55 (2.38 to 23.21)	12.98 (-2.13 to 24.18)
China	1.03 (-0.72 to 2.74)	1.63 (-1.20 to 4.35)	2.58 (-0.58 to 5.50)	2.79 (0.02 to 5.21)	13.52 (-4.98 to 27.38)	14.75 (-6.49 to 30.05)	19.49 (2.67 to 32.44)	22.43 (-1.30 to 38.39)
Colombia	0.50 (-0.81 to 1.74)	0.46 (-0.60 to 1.45)	0.62 (-0.80 to 1.94)	1.07 (-0.90 to 2.86)	8.98 (-12.42 to 27.19)	3.05 (-4.02 to 8.62)	4.89 (-4.04 to 12.77)	7.44 (-10.84 to 21.30)
Costa Rica	0.40 (-1.31 to 2.12)	0.21 (-1.04 to 1.47)	0.57 (-1.54 to 2.58)	0.97 (-2.04 to 3.83)	6.20 (-5.22 to 16.07)	4.92 (-8.97 to 16.37)	9.77 (-8.96 to 23.57)	7.61 (-5.25 to 18.13)
Czech Republic	0.34 (-0.37 to 0.99)	1.45 (-0.09 to 2.87)	3.63 (1.10 to 5.92)	4.91 (1.93 to 7.64)	/	/	9.22 (0.47 to 15.03)	26.37 (11.93 to 37.18)
Ecuador	0.15 (-0.68 to 1.00)	0.56 (-1.81 to 2.91)	1.15 (-1.44 to 3.60)	0.84 (-0.79 to 2.39)	7.59 (-12.45 to 21.34)	11.58 (-9.17 to 27.37)	7.42 (-3.31 to 16.62)	13.81 (-6.61 to 29.31)
Estonia	0.34 (-0.82 to 1.50)	0.82 (-0.86 to 2.43)	1.62 (-0.69 to 3.80)	1.96 (-1.24 to 4.91)	5.76 (-4.74 to 14.67)	14.86 (-4.45 to 29.80)	16.45 (-0.85 to 29.15)	8.01 (-14.39 to 23.75)
France	0.18 (-0.28 to 0.61)	0.35 (-0.53 to 1.19)	0.59 (-0.44 to 1.55)	0.63 (-0.34 to 1.53)	4.68 (-2.20 to 11.27)	7.12 (-0.22 to 13.82)	9.01 (2.46 to 15.02)	24.23 (19.51 to 28.25)
Germany	0.13 (-0.27 to 0.51)	0.54 (-0.46 to 1.50)	1.41 (-0.06 to 2.78)	2.13 (0.50 to 3.65)	4.73 (-2.76 to 11.63)	8.39 (0.66 to 15.39)	12.90 (7.70 to 17.84)	21.53 (16.59 to 26.06)
Greece	1.65 (0.09 to 3.10)	6.23 (3.89 to 8.33)	7.02 (4.28 to 9.42)	7.21 (3.15 to 10.78)	/	/	31.07 (22.59 to 38.92)	44.00 (34.40 to 51.90)
Guatemala	0.11 (-0.36 to 0.59)	0.01 (-0.11 to 0.12)	0.17 (-0.75 to 1.05)	0.42 (-1.36 to 2.11)	10.61 (-6.22 to 23.30)	3.29 (-7.99 to 12.63)	4.72 (-5.49 to 13.17)	4.38 (-2.08 to 10.54)
Iran	1.53 (-1.89 to 4.60)	3.15 (-0.71 to 6.76)	3.16 (-0.54 to 6.53)	2.53 (-0.65 to 5.47)	9.51 (-5.94 to 21.41)	16.83 (5.74 to 26.01)	20.86 (6.90 to 31.11)	23.47 (1.68 to 38.44)
Ireland	0.18 (-0.44 to 0.76)	0.42 (-0.43 to 1.22)	0.11 (-0.22 to 0.42)	0.64 (-0.60 to 1.78)	4.10 (-6.82 to 13.54)	5.44 (-5.57 to 14.42)	2.36 (-4.12 to 8.49)	7.81 (-4.69 to 17.72)
Italy	2.10 (0.10 to 4.00)	3.93 (1.11 to 6.57)	4.10 (1.27 to 6.70)	4.23 (1.52 to 6.72)	15.95 (4.13 to 25.72)	22.61 (10.80 to 31.84)	23.41 (11.05 to 33.37)	27.67 (9.82 to 39.98)
Japan	0.90 (0.10 to 1.64)	1.76 (0.52 to 2.92)	2.55 (1.12 to 3.89)	2.36 (1.04 to 3.58)	/	/	17.86 (7.48 to 26.89)	20.36 (7.04 to 31.97)
Mexico	0.22 (-0.29 to 0.70)	0.38 (-0.46 to 1.18)	1.14 (-0.47 to 2.65)	1.40 (-0.75 to 3.41)	27.01 (-111.76 to 130.51)	15.21 (-3.89 to 30.50)	12.62 (2.67 to 21.20)	9.45 (0.62 to 17.02)
Moldova	0.08 (-0.38 to 0.56)	0.35 (-0.97 to 1.67)	1.15 (-0.79 to 3.02)	3.98 (0.68 to 6.99)	/	/	/	63.11 (6.61 to 97.53)
Netherland	0.08 (-0.47 to 0.63)	0.41 (-0.85 to 1.60)	1.11 (-0.59 to 2.69)	2.23 (-0.38 to 4.63)	6.21 (-4.26 to 15.12)	10.23 (-2.13 to 20.22)	13.93 (4.24 to 22.67)	22.33 (11.18 to 31.24)
Panama	0.66 (-1.59 to 2.94)	2.87 (-3.54 to 8.30)	3.77 (-4.46 to 10.58)	1.38 (-1.38 to 3.96)	/	16.25 (-18.03 to 37.83)	18.49 (-18.27 to 41.41)	46.91 (-73.10 to 107.32)
Peru	0.55 (-1.40 to 2.50)	0.99 (-1.62 to 3.53)	0.79 (-1.04 to 2.53)	0.47 (-0.75 to 1.60)	8.57 (-5.98 to 20.49)	10.41 (-4.78 to 22.63)	12.16 (-4.62 to 25.08)	12.48 (-9.48 to 27.97)
Portugal	0.20 (-0.15 to 0.55)	1.08 (0.31 to 1.80)	1.75 (0.61 to 2.78)	2.73 (0.74 to 4.60)	11.31 (4.67 to 16.82)	22.30 (15.43 to 28.23)	20.83 (15.85 to 25.35)	25.15 (20.86 to 28.96)
Puerto Rico	0.33 (-2.04 to 2.70)	0.28 (-1.06 to 1.52)	0.42 (-1.41 to 2.18)	1.14 (-1.53 to 3.57)	7.19 (-13.54 to 24.88)	10.57 (-18.97 to 34.38)	8.55 (-14.84 to 26.35)	12.45 (-10.61 to 28.21)
Romania	0.66 (-0.28 to 1.59)	3.09 (1.06 to 4.93)	3.19 (0.98 to 5.29)	4.09 (2.00 to 6.04)	14.11 (3.66 to 22.78)	21.91 (12.26 to 30.27)	24.27 (13.33 to 32.96)	29.87 (18.76 to 38.28)
South Africa	0.39 (-0.86 to 1.59)	0.70 (-0.96 to 2.29)	0.88 (-1.07 to 2.70)	1.22 (-1.45 to 3.65)	7.87 (-5.73 to 18.89)	8.39 (-5.03 to 18.92)	9.29 (-5.85 to 21.12)	7.74 (-9.82 to 20.51)
South Korea	0.44 (-0.63 to 1.48)	0.90 (-0.51 to 2.25)	1.09 (-0.51 to 2.59)	0.65 (-0.24 to 1.48)	12.24 (-5.05 to 25.66)	17.49 (3.94 to 28.40)	12.41 (-0.96 to 23.67)	15.25 (-3.66 to 29.06)

Spain	1.31 (0.08 to 2.44)	2.83 (0.61 to 4.87)	3.32 (0.71 to 5.74)	3.51 (0.28 to 6.54)	13.21 (6.23 to 19.49)	16.55 (8.95 to 23.22)	19.43 (11.54 to 26.16)	21.88 (12.44 to 29.48)
Sweden	0.06 (-0.17 to 0.27)	0.40 (-0.45 to 1.21)	0.96 (-0.38 to 2.20)	2.01 (-0.04 to 3.91)	/	8.55 (-0.86 to 16.47)	15.18 (5.41 to 24.18)	13.40 (2.70 to 22.18)
Switzerland	0.33 (-0.58 to 1.22)	1.15 (-1.05 to 3.26)	2.52 (-0.82 to 5.64)	3.52 (-0.16 to 6.95)	18.41 (-1.21 to 34.02)	8.99 (-9.74 to 22.78)	14.78 (-9.31 to 32.06)	23.87 (2.12 to 39.17)
Thailand	0.34 (-0.91 to 1.58)	0.80 (-1.91 to 3.40)	1.64 (-1.71 to 4.76)	2.21 (-1.15 to 5.31)	10.50 (-1.73 to 21.51)	10.08 (-11.76 to 26.41)	14.15 (-5.02 to 28.75)	16.27 (-6.40 to 32.18)
UK	0.16 (-0.31 to 0.63)	0.36 (-0.47 to 1.15)	0.52 (-0.32 to 1.33)	1.09 (-0.10 to 2.21)	5.33 (-2.52 to 12.14)	7.23 (-2.39 to 15.33)	9.51 (-0.48 to 17.70)	13.60 (3.08 to 21.60)
Uruguay	0.50 (-0.13 to 1.10)	1.80 (-0.19 to 3.70)	2.87 (-0.00 to 5.53)	2.72 (0.32 to 4.98)	/	9.33 (2.08 to 15.26)	22.12 (9.08 to 32.29)	27.77 (6.81 to 42.10)
USA	0.63 (-0.70 to 1.90)	0.81 (-0.64 to 2.18)	0.97 (-0.47 to 2.31)	1.03 (-0.42 to 2.40)	8.14 (-1.11 to 16.24)	9.78 (-0.03 to 18.15)	12.17 (2.11 to 20.90)	11.55 (0.31 to 20.55)
Vietnam	2.45 (-0.33 to 5.13)	4.18 (0.71 to 7.47)	2.72 (-0.58 to 5.88)	2.12 (-0.10 to 4.14)	21.54 (11.65 to 29.37)	24.83 (11.41 to 34.96)	21.73 (10.12 to 30.66)	27.78 (11.68 to 41.01)
Total	0.66 (-0.35 to 1.62)	1.23 (-0.18 to 2.55)	1.71 (0.14 to 3.17)	1.83 (0.17 to 3.37)	8.61 (-1.82 to 17.49)	10.48 (-0.02 to 19.25)	12.90 (2.70 to 21.53)	16.36 (5.81 to 24.62)

Definition of abbreviations: TV = temperature variability; Q1 = the first quartile; Q2 = the second quartile; Q3 = the third quartile; Q4 = the fourth quartile; MMT = minimum mortality temperature; TVST = temperature variability sensitive heat threshold.

Table S9. Attributable fractions of mortality due to heat exposure stratified by quantiles of TV 0–2 in each country.

Country	Attributable fractions (%)			
	Q1 of TV 0–2	Q2 of TV 0–2	Q3 of TV 0–2	Q4 of TV 0–2
Argentina	1.22 (-0.50 to 2.85)	2.95 (0.82 to 4.96)	4.30 (2.21 to 6.24)	4.11 (1.82 to 6.23)
Australia	0.64 (-0.26 to 1.53)	0.41 (-0.42 to 1.21)	1.33 (-0.22 to 2.79)	2.66 (0.74 to 4.41)
Brazil	0.71 (-0.69 to 2.03)	1.63 (-0.75 to 3.85)	2.22 (-0.31 to 4.57)	3.00 (0.19 to 5.52)
Canada	0.58 (-0.54 to 1.65)	1.47 (-0.05 to 2.92)	1.31 (-0.30 to 2.77)	2.17 (0.29 to 3.82)
China	1.17 (-0.85 to 3.09)	1.79 (-1.08 to 4.52)	2.53 (-0.47 to 5.32)	3.37 (-0.00 to 6.21)
Colombia	0.78 (-0.49 to 1.98)	0.61 (-0.58 to 1.73)	0.56 (-0.87 to 1.88)	1.35 (-1.23 to 3.58)
Costa Rica	0.43 (-1.19 to 1.96)	0.42 (-1.37 to 2.26)	0.74 (-1.60 to 2.91)	1.10 (-1.89 to 3.89)
Czech Republic	0.29 (-0.32 to 0.87)	1.37 (-0.16 to 2.83)	3.75 (1.17 to 6.13)	4.73 (2.21 to 7.04)
Ecuador	0.29 (-0.33 to 0.89)	1.16 (-1.70 to 3.94)	1.39 (-1.33 to 3.92)	1.62 (-0.69 to 3.78)
Estonia	0.18 (-0.70 to 1.03)	0.88 (-0.88 to 2.58)	1.70 (-0.99 to 4.20)	2.49 (-1.95 to 6.46)
France	0.26 (-1.82 to 2.08)	0.63 (-0.38 to 1.59)	1.59 (-0.21 to 3.27)	9.36 (7.26 to 11.23)
Germany	0.09 (-0.24 to 0.42)	0.76 (-0.30 to 1.75)	2.03 (0.37 to 3.57)	7.69 (5.48 to 9.70)
Greece	0.88 (-0.39 to 2.08)	5.54 (3.30 to 7.55)	7.78 (4.82 to 10.43)	10.57 (6.59 to 13.99)
Guatemala	0.24 (-0.28 to 0.77)	0.17 (-0.44 to 0.77)	0.65 (-1.27 to 2.51)	0.53 (-1.52 to 2.45)
Iran	1.00 (-2.18 to 3.97)	3.54 (0.07 to 6.81)	3.34 (-0.40 to 6.84)	4.71 (1.23 to 7.92)
Ireland	0.20 (-0.28 to 0.66)	0.41 (-0.43 to 1.20)	0.20 (-0.52 to 0.88)	1.29 (-0.69 to 3.06)
Italy	1.93 (0.00 to 3.72)	3.96 (1.09 to 6.63)	4.57 (1.60 to 7.28)	4.91 (1.89 to 7.61)
Japan	0.97 (0.08 to 1.80)	1.85 (0.59 to 3.03)	2.22 (0.88 to 3.46)	2.38 (1.09 to 3.57)
Mexico	0.07 (-0.26 to 0.38)	0.42 (-0.58 to 1.38)	1.19 (-0.38 to 2.65)	2.72 (-0.07 to 5.31)
Moldova	0.08 (-0.28 to 0.43)	0.52 (-1.07 to 2.12)	0.96 (-1.11 to 2.89)	3.78 (0.58 to 6.59)
Netherland	0.07 (-0.33 to 0.47)	0.39 (-0.68 to 1.42)	1.69 (-0.42 to 3.66)	6.65 (3.57 to 9.45)
Panama	1.01 (-1.81 to 3.65)	4.23 (-2.74 to 9.75)	5.33 (-5.53 to 13.42)	1.57 (-1.57 to 4.50)
Peru	1.14 (-1.25 to 3.36)	1.70 (-1.44 to 4.64)	1.72 (-1.35 to 4.52)	1.27 (-0.89 to 3.05)
Portugal	0.14 (-0.18 to 0.45)	0.81 (0.03 to 1.53)	2.76 (1.35 to 4.04)	9.35 (6.94 to 11.48)
Puerto Rico	0.60 (-2.22 to 3.24)	0.82 (-1.81 to 3.36)	0.55 (-1.43 to 2.34)	1.90 (-1.82 to 5.22)
Romania	0.74 (-0.28 to 1.69)	2.14 (0.31 to 3.82)	4.08 (1.77 to 6.24)	5.96 (3.62 to 8.15)
South Africa	0.44 (-0.83 to 1.65)	0.73 (-0.98 to 2.35)	1.16 (-1.08 to 3.24)	1.86 (-1.10 to 4.41)
South Korea	0.50 (-0.61 to 1.57)	1.12 (-0.54 to 2.71)	1.09 (-0.55 to 2.59)	0.87 (-0.24 to 1.82)
Spain	1.18 (-0.08 to 2.33)	2.97 (0.74 to 5.02)	4.80 (1.69 to 7.62)	6.91 (3.26 to 10.12)
Sweden	0.04 (-0.11 to 0.19)	0.60 (-0.58 to 1.71)	1.33 (-0.09 to 2.64)	2.83 (0.12 to 5.28)
Switzerland	0.21 (-0.56 to 0.94)	1.21 (-1.09 to 3.43)	2.07 (-1.30 to 5.23)	5.85 (0.69 to 10.34)
Thailand	0.34 (-0.89 to 1.51)	0.94 (-1.70 to 3.50)	2.27 (-1.63 to 5.84)	3.57 (-1.18 to 7.67)
UK	0.11 (-0.29 to 0.49)	0.28 (-0.43 to 0.98)	0.51 (-0.35 to 1.33)	2.69 (0.55 to 4.61)
Uruguay	0.28 (-0.41 to 0.92)	2.60 (0.43 to 4.64)	4.77 (1.61 to 7.75)	5.31 (1.77 to 8.56)
USA	0.67 (-0.64 to 1.89)	1.08 (-0.54 to 2.61)	1.28 (-0.34 to 2.78)	1.82 (-0.07 to 3.52)
Vietnam	2.39 (-0.28 to 4.83)	4.98 (1.27 to 8.46)	3.03 (-0.56 to 6.60)	2.51 (-0.17 to 4.92)
Total	0.69 (-0.36 to 1.68)	1.36 (-0.12 to 2.76)	1.81 (0.14 to 3.36)	2.78 (0.81 to 4.55)

Definition of abbreviations: TV = temperature variability; Q1 = the first quartile; Q2 = the second quartile; Q3 = the third quartile; Q4 = the fourth quartile.

Table S10. Attributable fractions of mortality due to heat exposure stratified by quantiles of TV 0–3 in each country.

Country	Attributable fractions (%)			
	Q1 of TV 0–3	Q2 of TV 0–3	Q3 of TV 0–3	Q4 of TV 0–3
Argentina	1.54 (-0.18 to 3.19)	2.39 (0.24 to 4.39)	3.89 (1.77 to 5.85)	4.12 (1.68 to 6.40)
Australia	0.76 (-0.27 to 1.74)	0.52 (-0.38 to 1.35)	1.31 (-0.15 to 2.70)	2.06 (0.23 to 3.74)
Brazil	0.82 (-0.69 to 2.24)	1.70 (-0.78 to 3.97)	2.09 (-0.55 to 4.51)	3.03 (0.38 to 5.41)
Canada	0.71 (-0.46 to 1.81)	1.19 (-0.37 to 2.63)	1.62 (-0.15 to 3.25)	1.74 (0.08 to 3.18)
China	1.09 (-0.82 to 2.97)	1.88 (-1.16 to 4.70)	2.38 (-0.89 to 5.34)	3.35 (-0.02 to 6.10)
Colombia	0.73 (-0.68 to 2.05)	0.44 (-0.84 to 1.59)	1.03 (-1.26 to 3.18)	1.00 (-2.06 to 3.76)
Costa Rica	0.46 (-1.21 to 2.11)	0.61 (-1.73 to 2.82)	0.66 (-1.81 to 2.95)	0.97 (-1.68 to 3.43)
Czech Republic	0.35 (-0.39 to 1.05)	1.93 (0.14 to 3.56)	3.71 (1.23 to 6.00)	4.25 (1.84 to 6.47)
Ecuador	0.20 (-0.41 to 0.80)	1.31 (-1.56 to 3.98)	1.71 (-1.72 to 4.89)	1.41 (-0.62 to 3.35)
Estonia	0.18 (-0.67 to 0.99)	1.09 (-0.80 to 2.83)	1.47 (-1.28 to 4.04)	2.85 (-1.68 to 6.88)
France	0.37 (-1.71 to 2.17)	0.74 (-0.45 to 1.81)	1.60 (-0.17 to 3.23)	8.83 (6.68 to 10.72)
Germany	0.12 (-0.27 to 0.48)	0.75 (-0.39 to 1.78)	2.42 (0.61 to 4.08)	7.44 (5.13 to 9.54)
Greece	1.00 (-0.25 to 2.19)	6.69 (4.11 to 8.83)	8.70 (5.67 to 11.43)	11.28 (7.16 to 14.85)
Guatemala	0.08 (-0.13 to 0.29)	0.36 (-0.83 to 1.48)	0.73 (-1.63 to 2.95)	0.38 (-0.78 to 1.45)
Iran	2.03 (-1.69 to 5.42)	2.73 (-0.94 to 6.07)	3.05 (-0.52 to 6.36)	5.25 (1.33 to 8.81)
Ireland	0.24 (-0.45 to 0.91)	0.18 (-0.33 to 0.66)	0.41 (-0.57 to 1.33)	1.05 (-1.05 to 2.99)
Italy	1.85 (-0.03 to 3.62)	4.46 (1.41 to 7.21)	4.87 (1.77 to 7.71)	4.43 (1.41 to 7.12)
Japan	1.09 (0.15 to 1.98)	1.83 (0.52 to 3.03)	2.13 (0.82 to 3.37)	2.15 (0.93 to 3.29)
Mexico	0.11 (-0.29 to 0.48)	0.47 (-0.70 to 1.55)	0.82 (-0.41 to 1.99)	2.76 (0.22 to 5.12)
Moldova	0.06 (-0.27 to 0.40)	0.85 (-1.21 to 2.78)	0.89 (-1.17 to 2.83)	3.53 (0.47 to 6.08)
Netherland	0.07 (-0.28 to 0.42)	0.74 (-0.79 to 2.12)	1.50 (-0.64 to 3.50)	6.70 (3.80 to 9.35)
Panama	0.75 (-1.46 to 2.91)	4.71 (-3.57 to 11.17)	5.00 (-5.83 to 13.52)	1.39 (-1.62 to 4.28)
Peru	1.21 (-1.30 to 3.61)	1.52 (-1.55 to 4.36)	1.92 (-1.38 to 4.92)	1.05 (-1.08 to 2.75)
Portugal	0.15 (-0.20 to 0.48)	0.98 (0.04 to 1.83)	3.04 (1.48 to 4.44)	8.85 (6.66 to 10.81)
Puerto Rico	0.57 (-1.57 to 2.63)	0.66 (-1.92 to 3.08)	0.51 (-1.36 to 2.08)	1.82 (-2.18 to 5.36)
Romania	0.83 (-0.24 to 1.85)	1.89 (0.14 to 3.54)	4.07 (1.75 to 6.25)	5.96 (3.58 to 8.18)
South Africa	0.41 (-0.77 to 1.54)	0.76 (-0.98 to 2.35)	1.14 (-1.58 to 3.61)	1.85 (-1.45 to 4.67)
South Korea	0.55 (-0.58 to 1.65)	1.16 (-0.72 to 2.87)	1.03 (-0.50 to 2.40)	0.74 (-0.25 to 1.58)
Spain	1.25 (-0.00 to 2.40)	3.12 (0.66 to 5.35)	4.99 (1.87 to 7.83)	6.85 (2.99 to 10.22)
Sweden	0.03 (-0.07 to 0.14)	0.41 (-0.60 to 1.36)	1.37 (-0.45 to 3.05)	3.41 (0.79 to 5.77)
Switzerland	0.19 (-0.55 to 0.89)	0.91 (-1.38 to 3.04)	1.93 (-1.57 to 5.19)	6.08 (0.78 to 10.75)
Thailand	0.35 (-1.03 to 1.66)	0.85 (-1.87 to 3.36)	2.44 (-1.69 to 6.23)	3.38 (-1.34 to 7.39)
UK	0.11 (-0.26 to 0.46)	0.23 (-0.43 to 0.85)	0.53 (-0.41 to 1.43)	2.51 (0.45 to 4.34)
Uruguay	0.70 (-0.26 to 1.60)	2.05 (-0.07 to 4.03)	4.45 (1.45 to 7.23)	3.33 (-1.27 to 7.23)
USA	0.72 (-0.64 to 2.01)	1.08 (-0.59 to 2.61)	1.32 (-0.39 to 2.91)	1.71 (-0.13 to 3.36)
Vietnam	2.74 (0.07 to 5.33)	3.60 (-0.30 to 7.22)	4.47 (0.52 to 8.17)	2.12 (-0.68 to 4.62)
Total	0.76 (-0.33 to 1.80)	1.35 (-0.19 to 2.76)	1.82 (0.08 to 3.44)	2.61 (0.67 to 4.34)

Definition of abbreviations: TV = temperature variability; Q1 = the first quartile; Q2 = the second quartile; Q3 = the third quartile; Q4 = the fourth quartile.

Table S11. Attributable fractions of mortality due to heat exposure stratified by TVST for each quartile of TV 0–2 in each country.

Country	Attributable fractions (%): MMT–TVST				Attributable fractions (%): above TVST			
	Q1 of TV 0–2	Q2 of TV 0–2	Q3 of TV 0–2	Q4 of TV 0–2	Q1 of TV 0–2	Q2 of TV 0–2	Q3 of TV 0–2	Q4 of TV 0–2
Argentina	1.19 (-0.52 to 2.82)	2.57 (0.53 to 4.48)	2.90 (0.92 to 4.74)	2.57 (0.41 to 4.58)	10.78 (0.31 to 20.27)	15.25 (4.90 to 23.72)	27.77 (19.57 to 34.71)	19.77 (10.64 to 27.30)
Australia	0.64 (-0.27 to 1.52)	0.41 (-0.42 to 1.21)	1.33 (-0.22 to 2.79)	2.45 (0.62 to 4.09)	13.90 (-11.05 to 30.02)	/	/	34.67 (3.66 to 55.03)
Brazil	0.61 (-0.76 to 1.90)	1.50 (-0.81 to 3.66)	1.84 (-0.59 to 4.09)	2.12 (-0.49 to 4.50)	9.79 (-3.65 to 21.26)	11.86 (-1.82 to 22.96)	14.82 (3.89 to 23.91)	16.36 (2.86 to 26.76)
Canada	0.52 (-0.58 to 1.57)	1.20 (-0.24 to 2.56)	0.99 (-0.39 to 2.27)	1.21 (-0.16 to 2.49)	7.92 (-0.31 to 15.10)	12.55 (3.95 to 19.91)	8.81 (-0.92 to 17.03)	12.27 (0.69 to 21.19)
China	0.97 (-0.97 to 2.79)	1.57 (-1.14 to 4.20)	2.20 (-0.58 to 4.84)	2.50 (-0.20 to 4.91)	10.79 (-6.14 to 23.88)	15.98 (-3.43 to 29.98)	17.72 (-0.34 to 31.46)	23.27 (0.25 to 38.98)
Colombia	0.73 (-0.50 to 1.90)	0.56 (-0.58 to 1.64)	0.45 (-0.82 to 1.62)	0.97 (-0.93 to 2.74)	5.28 (-4.61 to 14.07)	4.10 (-3.27 to 10.25)	3.73 (-5.51 to 11.33)	6.82 (-10.32 to 20.37)
Costa Rica	0.23 (-1.14 to 1.54)	0.27 (-1.28 to 1.86)	0.35 (-1.28 to 1.96)	0.67 (-2.17 to 3.33)	4.53 (-3.55 to 12.29)	3.94 (-6.18 to 12.47)	6.41 (-7.50 to 17.17)	6.39 (-4.59 to 15.96)
Czech Republic	0.29 (-0.32 to 0.87)	1.37 (-0.16 to 2.82)	3.44 (0.90 to 5.78)	3.29 (0.93 to 5.52)	/	13.93 (0.55 to 24.43)	22.50 (13.35 to 31.00)	22.83 (12.43 to 31.24)
Ecuador	0.10 (-0.35 to 0.55)	0.80 (-1.73 to 3.24)	1.03 (-1.37 to 3.26)	0.73 (-1.06 to 2.39)	5.82 (-4.59 to 13.92)	8.13 (-5.35 to 18.91)	8.99 (-2.56 to 18.75)	11.38 (-3.45 to 23.26)
Estonia	0.18 (-0.70 to 1.01)	0.82 (-0.87 to 2.46)	0.99 (-1.11 to 2.97)	1.74 (-1.53 to 4.77)	7.35 (-5.47 to 18.94)	9.48 (-5.01 to 21.25)	12.04 (-4.10 to 24.42)	7.07 (-13.02 to 21.78)
France	0.21 (-1.83 to 2.00)	0.38 (-0.44 to 1.16)	0.67 (-0.60 to 1.85)	0.56 (-0.36 to 1.43)	4.05 (-1.83 to 9.83)	7.24 (0.16 to 13.73)	8.47 (1.86 to 14.53)	23.41 (18.80 to 27.36)
Germany	0.09 (-0.24 to 0.42)	0.69 (-0.33 to 1.64)	1.35 (-0.12 to 2.72)	1.86 (0.33 to 3.29)	4.01 (-3.61 to 11.57)	9.73 (2.04 to 16.66)	11.78 (6.14 to 17.14)	20.90 (16.04 to 25.33)
Greece	0.80 (-0.40 to 1.95)	4.97 (2.84 to 6.91)	5.47 (2.81 to 7.79)	4.56 (1.04 to 7.70)	7.51 (-0.13 to 14.72)	15.28 (10.82 to 19.44)	21.93 (15.80 to 27.55)	25.49 (19.15 to 31.13)
Guatemala	0.06 (-0.33 to 0.42)	0.04 (-0.33 to 0.42)	0.26 (-1.09 to 1.54)	0.38 (-1.50 to 2.17)	7.66 (-4.07 to 17.69)	4.32 (-5.12 to 12.37)	4.47 (-4.55 to 12.74)	3.09 (-2.16 to 7.94)
Iran	0.95 (-2.26 to 3.92)	3.20 (-0.25 to 6.39)	2.75 (-0.98 to 6.18)	2.97 (-0.26 to 5.88)	7.14 (-5.82 to 18.01)	15.50 (4.94 to 23.95)	19.24 (4.97 to 30.03)	32.01 (9.94 to 46.76)
Ireland	0.14 (-0.25 to 0.51)	0.30 (-0.41 to 0.97)	0.11 (-0.39 to 0.58)	0.60 (-0.69 to 1.82)	3.93 (-4.41 to 11.32)	4.52 (-3.36 to 11.69)	2.59 (-6.57 to 10.59)	6.46 (-4.27 to 15.37)
Italy	1.71 (-0.17 to 3.45)	3.38 (0.67 to 5.91)	3.96 (1.15 to 6.53)	3.44 (0.65 to 6.00)	10.82 (2.61 to 17.97)	17.83 (7.78 to 26.07)	20.31 (9.59 to 28.81)	20.24 (7.70 to 29.84)
Japan	0.97 (0.08 to 1.80)	1.85 (0.59 to 3.03)	2.20 (0.87 to 3.45)	2.35 (1.06 to 3.53)	11.82 (-36.66 to 54.47)	12.99 (4.08 to 21.07)	18.90 (9.36 to 27.43)	17.35 (4.73 to 28.67)
Mexico	0.07 (-0.26 to 0.38)	0.38 (-0.60 to 1.31)	0.90 (-0.51 to 2.23)	1.55 (-1.03 to 3.92)	20.29 (-13.73 to 47.23)	14.45 (-5.75 to 30.70)	9.86 (1.44 to 17.25)	9.20 (1.67 to 15.72)
Moldova	0.08 (-0.29 to 0.43)	0.49 (-1.06 to 2.06)	0.66 (-1.05 to 2.35)	1.35 (-0.75 to 3.28)	0.00 (0.00 to 0.00)	3.49 (-2.79 to 8.76)	5.97 (-2.89 to 13.33)	16.05 (2.43 to 26.44)
Netherland	0.05 (-0.34 to 0.42)	0.30 (-0.66 to 1.23)	1.05 (-0.78 to 2.73)	1.20 (-0.58 to 2.92)	4.89 (-3.06 to 12.17)	6.01 (-3.73 to 14.52)	10.62 (2.38 to 18.14)	19.37 (11.08 to 26.39)
Panama	1.01 (-1.81 to 3.65)	2.66 (-2.33 to 6.95)	2.88 (-3.69 to 8.71)	1.57 (-1.57 to 4.50)	/	26.95 (-16.74 to 57.75)	21.96 (-18.63 to 48.09)	/
Peru	0.54 (-1.38 to 2.35)	0.97 (-1.45 to 3.36)	0.87 (-1.33 to 2.95)	0.40 (-0.73 to 1.46)	8.71 (-3.82 to 19.42)	10.15 (-3.87 to 21.67)	11.05 (-4.85 to 23.49)	13.44 (-10.84 to 29.73)
Portugal	0.13 (-0.18 to 0.43)	0.63 (-0.09 to 1.31)	1.64 (0.44 to 2.72)	1.73 (-0.00 to 3.40)	5.61 (-0.05 to 10.97)	15.57 (9.19 to 21.30)	16.21 (11.36 to 20.76)	21.30 (17.24 to 24.97)
Puerto Rico	0.57 (-2.28 to 3.26)	0.52 (-1.63 to 2.72)	0.35 (-1.24 to 1.84)	1.00 (-2.11 to 3.87)	3.65 (-2.46 to 9.00)	8.46 (-8.88 to 22.61)	8.59 (-15.25 to 27.80)	11.67 (-13.28 to 29.23)
Romania	0.70 (-0.32 to 1.65)	2.10 (0.28 to 3.77)	3.83 (1.55 to 5.95)	4.10 (2.07 to 6.02)	13.40 (3.92 to 21.35)	19.53 (7.71 to 29.82)	22.73 (12.90 to 31.21)	29.56 (18.34 to 38.06)
South Africa	0.38 (-0.86 to 1.55)	0.56 (-0.96 to 2.03)	0.74 (-1.09 to 2.45)	1.08 (-1.04 to 3.06)	6.68 (-2.53 to 14.81)	6.56 (-4.26 to 15.41)	7.91 (-4.32 to 17.92)	7.77 (-6.81 to 18.70)
South Korea	0.50 (-0.61 to 1.57)	1.12 (-0.54 to 2.71)	1.09 (-0.54 to 2.59)	0.84 (-0.25 to 1.78)	/	/	13.08 (-14.58 to 31.43)	24.49 (-6.79 to 44.49)

Spain	1.10 (-0.13 to 2.22)	2.56 (0.43 to 4.52)	3.82 (0.90 to 6.48)	3.61 (0.47 to 6.43)	9.67 (3.35 to 15.55)	15.65 (8.65 to 21.92)	18.60 (10.54 to 25.51)	22.49 (13.10 to 29.98)
Sweden	0.04 (-0.11 to 0.18)	0.55 (-0.60 to 1.65)	0.96 (-0.30 to 2.13)	1.42 (-0.48 to 3.20)	4.45 (-2.93 to 11.00)	10.94 (1.06 to 19.33)	12.68 (3.96 to 20.58)	9.88 (0.27 to 18.01)
Switzerland	0.19 (-0.57 to 0.91)	1.13 (-1.11 to 3.28)	1.73 (-1.40 to 4.64)	2.88 (-0.75 to 6.29)	7.55 (-4.03 to 17.23)	11.31 (-3.74 to 23.55)	12.34 (-3.24 to 25.12)	18.99 (2.10 to 31.55)
Thailand	0.31 (-0.90 to 1.45)	0.87 (-1.70 to 3.38)	1.84 (-1.69 to 5.14)	2.23 (-1.40 to 5.57)	7.60 (-4.91 to 17.37)	11.39 (-5.58 to 24.77)	13.91 (-5.17 to 28.29)	15.60 (-7.88 to 32.08)
UK	0.09 (-0.29 to 0.46)	0.23 (-0.42 to 0.87)	0.29 (-0.37 to 0.92)	0.82 (-0.50 to 2.08)	3.55 (-2.20 to 8.82)	4.65 (-2.60 to 11.00)	5.71 (-1.03 to 11.71)	10.68 (2.65 to 17.22)
Uruguay	0.28 (-0.41 to 0.92)	2.25 (0.18 to 4.14)	2.99 (0.00 to 5.79)	2.71 (-0.17 to 5.41)	/	17.75 (7.84 to 26.08)	25.72 (14.24 to 34.55)	21.60 (4.40 to 34.02)
USA	0.49 (-0.72 to 1.63)	0.69 (-0.70 to 2.01)	0.66 (-0.58 to 1.81)	0.78 (-0.66 to 2.14)	6.03 (-0.57 to 11.96)	7.52 (0.13 to 14.15)	8.52 (0.84 to 15.30)	9.51 (0.89 to 16.71)
Vietnam	1.56 (-1.06 to 3.99)	3.18 (-0.21 to 6.41)	1.84 (-1.56 to 5.10)	1.77 (-0.68 to 4.05)	18.02 (8.41 to 25.90)	23.59 (10.32 to 34.02)	18.05 (5.39 to 28.28)	26.53 (6.33 to 41.65)
Total	0.62 (-0.39 to 1.57)	1.20 (-0.18 to 2.50)	1.48 (0.01 to 2.85)	1.68 (0.08 to 3.15)	6.45 (-0.95 to 13.03)	8.34 (0.19 to 15.47)	9.87 (1.44 to 17.16)	13.78 (4.76 to 21.09)

Definition of abbreviations: TV = temperature variability; Q1 = the first quartile; Q2 = the second quartile; Q3 = the third quartile; Q4 = the fourth quartile; MMT = minimum mortality temperature; TVST = temperature variability sensitive heat threshold.

Table S12. Attributable fractions of mortality due to heat exposure stratified by TVST for each quartile of TV 0–3 in each country.

Country	Attributable fractions (%): MMT–TVST				Attributable fractions (%): above TVST			
	Q1 of TV 0–3	Q2 of TV 0–3	Q3 of TV 0–3	Q4 of TV 0–3	Q1 of TV 0–3	Q2 of TV 0–3	Q3 of TV 0–3	Q4 of TV 0–3
Argentina	1.49 (-0.23 to 3.12)	2.02 (-0.05 to 3.96)	2.67 (0.73 to 4.51)	2.65 (0.32 to 4.82)	9.41 (1.68 to 16.35)	15.26 (3.33 to 25.55)	24.77 (16.51 to 31.74)	19.30 (10.14 to 26.95)
Australia	0.70 (-0.28 to 1.63)	0.45 (-0.36 to 1.18)	0.90 (-0.34 to 2.08)	0.67 (-0.46 to 1.75)	5.26 (-1.40 to 11.36)	5.21 (-3.71 to 13.23)	10.60 (2.81 to 17.85)	12.20 (1.01 to 21.15)
Brazil	0.79 (-0.72 to 2.21)	1.67 (-0.80 to 3.92)	1.98 (-0.64 to 4.37)	2.68 (0.16 to 4.97)	14.68 (-4.24 to 28.84)	12.50 (-6.48 to 26.73)	17.70 (4.13 to 28.50)	23.30 (3.14 to 37.24)
Canada	0.69 (-0.47 to 1.78)	1.04 (-0.46 to 2.43)	1.39 (-0.27 to 2.94)	1.11 (-0.10 to 2.24)	7.48 (-1.79 to 15.51)	13.71 (2.21 to 23.42)	12.44 (1.30 to 21.70)	12.84 (-0.95 to 22.98)
China	0.79 (-0.92 to 2.47)	1.40 (-1.37 to 3.96)	1.93 (-0.99 to 4.62)	1.77 (-0.32 to 3.68)	8.70 (-3.28 to 19.41)	13.97 (-2.62 to 26.89)	14.64 (-2.06 to 27.60)	19.24 (0.40 to 32.59)
Colombia	0.67 (-0.67 to 1.94)	0.40 (-0.84 to 1.52)	0.83 (-1.19 to 2.71)	0.81 (-1.62 to 3.12)	6.42 (-4.55 to 16.35)	2.58 (-5.80 to 9.52)	6.51 (-6.46 to 18.19)	3.98 (-17.92 to 20.45)
Costa Rica	0.28 (-1.22 to 1.70)	0.47 (-1.69 to 2.48)	0.36 (-1.54 to 2.21)	0.55 (-1.94 to 2.87)	4.65 (-3.30 to 12.44)	5.31 (-6.04 to 15.54)	6.08 (-8.17 to 17.50)	6.46 (-5.32 to 15.79)
Czech Republic	0.35 (-0.39 to 1.05)	1.93 (0.14 to 3.56)	3.62 (1.15 to 5.90)	3.54 (1.24 to 5.70)	/	/	25.92 (16.50 to 34.46)	25.91 (12.49 to 36.25)
Ecuador	0.09 (-0.37 to 0.55)	0.92 (-1.67 to 3.30)	1.27 (-1.69 to 4.05)	0.64 (-0.93 to 2.11)	4.99 (-3.86 to 12.54)	11.83 (-9.61 to 27.14)	9.91 (-4.31 to 21.94)	11.67 (-4.02 to 24.18)
Estonia	0.17 (-0.67 to 0.98)	1.01 (-0.82 to 2.71)	1.01 (-1.28 to 3.17)	1.84 (-1.53 to 4.97)	8.64 (-4.86 to 20.41)	11.01 (-3.68 to 23.20)	11.31 (-6.37 to 24.96)	9.99 (-12.12 to 25.81)
France	0.33 (-1.70 to 2.08)	0.47 (-0.51 to 1.36)	0.76 (-0.47 to 1.89)	0.41 (-0.50 to 1.23)	3.74 (-2.38 to 9.83)	7.72 (0.34 to 14.33)	7.74 (1.23 to 13.75)	22.48 (17.75 to 26.55)
Germany	0.12 (-0.27 to 0.48)	0.68 (-0.42 to 1.68)	1.66 (0.02 to 3.17)	1.80 (0.11 to 3.37)	3.22 (-3.75 to 10.02)	9.54 (1.33 to 17.03)	13.09 (7.48 to 18.40)	20.35 (15.44 to 24.83)
Greece	1.00 (-0.25 to 2.19)	6.38 (3.84 to 8.51)	7.59 (4.60 to 10.23)	7.06 (2.98 to 10.65)	/	23.32 (16.97 to 29.14)	24.90 (19.01 to 30.14)	34.30 (26.37 to 41.28)
Guatemala	0.01 (-0.12 to 0.14)	0.13 (-0.80 to 1.01)	0.32 (-1.42 to 1.96)	0.20 (-0.78 to 1.12)	5.64 (-3.95 to 14.39)	8.27 (-6.84 to 20.10)	4.88 (-5.10 to 13.74)	3.13 (-1.64 to 7.68)
Iran	2.03 (-1.69 to 5.42)	2.68 (-0.99 to 6.05)	2.77 (-0.76 to 6.09)	4.35 (0.56 to 7.75)	/	19.22 (0.84 to 33.13)	27.80 (9.37 to 39.96)	31.85 (6.33 to 48.52)
Ireland	0.18 (-0.45 to 0.80)	0.12 (-0.25 to 0.48)	0.28 (-0.42 to 0.94)	0.62 (-1.09 to 2.22)	4.70 (-3.11 to 12.04)	2.42 (-5.51 to 9.99)	4.09 (-5.98 to 12.91)	4.64 (-6.44 to 13.85)
Italy	1.74 (-0.12 to 3.48)	4.15 (1.14 to 6.87)	4.38 (1.38 to 7.12)	3.77 (0.96 to 6.37)	14.67 (5.35 to 22.58)	24.74 (11.94 to 35.26)	27.61 (13.52 to 38.31)	23.87 (4.22 to 37.48)
Japan	1.01 (0.08 to 1.88)	1.57 (0.32 to 2.73)	1.60 (0.41 to 2.73)	1.46 (0.40 to 2.44)	9.02 (3.32 to 14.47)	9.28 (4.01 to 14.34)	10.39 (5.15 to 15.26)	9.83 (4.42 to 14.90)
Mexico	0.11 (-0.29 to 0.48)	0.45 (-0.72 to 1.50)	0.63 (-0.46 to 1.65)	1.55 (-0.68 to 3.65)	24.72 (-16.70 to 59.26)	15.71 (-10.17 to 36.41)	8.65 (-1.11 to 17.22)	10.40 (2.67 to 17.09)
Moldova	0.06 (-0.27 to 0.40)	0.82 (-1.24 to 2.75)	0.67 (-1.13 to 2.46)	1.19 (-0.41 to 2.80)	/	2.90 (-0.98 to 6.10)	4.75 (-2.51 to 10.82)	16.81 (1.77 to 28.16)
Netherland	0.05 (-0.28 to 0.38)	0.59 (-0.81 to 1.88)	0.89 (-0.92 to 2.57)	1.14 (-0.50 to 2.70)	3.59 (-3.25 to 10.16)	7.56 (-1.29 to 15.52)	9.76 (0.75 to 17.83)	20.07 (12.02 to 26.82)
Panama	0.75 (-1.46 to 2.91)	2.87 (-2.41 to 7.48)	3.70 (-4.94 to 10.89)	1.39 (-1.62 to 4.28)	/	24.92 (-20.23 to 54.12)	30.34 (-28.17 to 69.57)	/
Peru	1.10 (-1.32 to 3.43)	1.40 (-1.55 to 4.13)	1.67 (-1.44 to 4.54)	0.80 (-0.90 to 2.26)	12.91 (-5.95 to 27.13)	19.26 (-13.71 to 43.46)	23.22 (-7.23 to 43.51)	28.06 (-31.42 to 61.97)
Portugal	0.14 (-0.21 to 0.47)	0.80 (-0.08 to 1.59)	1.90 (0.59 to 3.07)	1.24 (-0.18 to 2.60)	6.55 (0.78 to 12.20)	13.11 (6.92 to 18.76)	15.35 (10.29 to 20.08)	21.38 (17.27 to 25.03)
Puerto Rico	0.45 (-1.61 to 2.44)	0.61 (-1.90 to 2.96)	0.19 (-0.72 to 1.00)	1.08 (-2.31 to 4.17)	4.39 (-2.33 to 10.35)	9.85 (-11.98 to 28.79)	6.71 (-15.50 to 24.48)	9.85 (-17.64 to 28.07)
Romania	0.80 (-0.28 to 1.81)	1.83 (0.08 to 3.47)	3.88 (1.60 to 6.03)	4.08 (1.99 to 6.05)	12.50 (1.95 to 21.13)	21.35 (8.97 to 31.98)	20.10 (10.48 to 28.24)	29.65 (18.45 to 38.14)
South Africa	0.35 (-0.78 to 1.44)	0.61 (-0.96 to 2.06)	0.82 (-1.54 to 2.99)	1.14 (-1.40 to 3.44)	6.80 (-2.43 to 15.14)	7.51 (-5.37 to 18.23)	7.29 (-5.82 to 17.85)	7.90 (-7.58 to 19.30)
South Korea	0.48 (-0.60 to 1.52)	0.81 (-0.81 to 2.31)	0.50 (-0.48 to 1.43)	0.27 (-0.27 to 0.77)	7.16 (-2.06 to 15.28)	8.12 (-0.57 to 15.88)	7.45 (-1.79 to 15.09)	10.85 (-3.68 to 21.71)

Spain	1.17 (-0.04 to 2.29)	2.68 (0.31 to 4.83)	4.01 (1.06 to 6.67)	3.58 (0.16 to 6.63)	9.40 (3.21 to 15.36)	15.76 (8.34 to 22.22)	18.76 (10.62 to 25.84)	22.46 (13.00 to 30.05)
Sweden	0.03 (-0.07 to 0.14)	0.36 (-0.63 to 1.30)	1.04 (-0.62 to 2.59)	1.74 (-0.12 to 3.51)	/	8.82 (-1.55 to 17.97)	11.29 (2.28 to 19.23)	12.98 (2.93 to 21.33)
Switzerland	0.17 (-0.56 to 0.87)	0.83 (-1.38 to 2.89)	1.55 (-1.62 to 4.55)	3.49 (-0.53 to 7.25)	8.06 (-1.71 to 16.24)	9.95 (-7.91 to 24.32)	11.70 (-3.51 to 23.99)	17.88 (1.12 to 30.42)
Thailand	0.32 (-1.04 to 1.62)	0.79 (-1.87 to 3.24)	2.03 (-1.72 to 5.55)	2.09 (-1.40 to 5.32)	7.85 (-3.17 to 17.65)	8.55 (-9.02 to 22.34)	13.67 (-4.89 to 27.93)	14.97 (-8.94 to 31.70)
UK	0.11 (-0.26 to 0.46)	0.23 (-0.43 to 0.84)	0.50 (-0.42 to 1.37)	1.73 (0.10 to 3.25)	6.25 (-3.61 to 14.34)	7.26 (-7.40 to 18.76)	9.64 (-2.31 to 19.37)	16.05 (2.15 to 26.09)
Uruguay	0.70 (-0.26 to 1.60)	1.75 (-0.32 to 3.62)	3.35 (0.53 to 5.93)	2.20 (-0.94 to 5.10)	/	15.56 (4.84 to 24.68)	26.13 (14.88 to 35.15)	10.19 (-13.37 to 26.51)
USA	0.61 (-0.70 to 1.86)	0.85 (-0.70 to 2.29)	0.94 (-0.56 to 2.34)	1.01 (-0.51 to 2.43)	7.69 (-0.06 to 14.78)	9.99 (0.36 to 18.45)	11.04 (1.20 to 19.43)	11.34 (0.18 to 20.17)
Vietnam	1.90 (-0.72 to 4.36)	2.41 (-1.13 to 5.69)	3.62 (-0.27 to 7.20)	1.59 (-1.03 to 4.07)	23.76 (11.25 to 34.12)	15.94 (1.31 to 27.45)	25.55 (10.88 to 37.58)	23.59 (-1.58 to 40.36)
Total	0.69 (-0.37 to 1.69)	1.14 (-0.31 to 2.48)	1.37 (-0.19 to 2.83)	1.44 (-0.13 to 2.88)	8.26 (0.90 to 15.02)	9.69 (1.87 to 16.68)	10.90 (3.17 to 17.64)	13.82 (4.97 to 20.99)

Definition of abbreviations: TV = temperature variability; Q1 = the first quartile; Q2 = the second quartile; Q3 = the third quartile; Q4 = the fourth quartile; MMT = minimum mortality temperature; TVST = temperature variability sensitive heat threshold.

Table S13. Sensitivity analyses of overall attributable fractions after adding separate predictor in meta-regression with BLUP, stratified by quartiles of TV.

Predictors	Attributable fractions (%)			
	Q1 of TV	Q2 of TV	Q3 of TV	Q4 of TV
Without predictors	0.70 (-0.33 to 1.69)	1.34 (-0.14 to 2.73)	1.99 (0.29 to 3.57)	2.73 (0.76 to 4.50)
Region	0.79 (-0.27 to 1.76)	1.35 (0.06 to 2.55)	1.99 (0.47 to 3.40)	2.88 (1.01 to 4.56)
Average mean temperature	0.75 (-0.27 to 1.72)	1.37 (-0.13 to 2.78)	2.00 (0.30 to 3.58)	2.74 (0.75 to 4.52)
Range of mean temperature	0.71 (-0.33 to 1.72)	1.36 (-0.16 to 2.80)	1.99 (0.26 to 3.60)	2.74 (0.73 to 4.54)
Koeppen-Geiger Climatic Zones 1st	0.75 (-0.45 to 1.88)	1.39 (-0.22 to 2.88)	2.02 (0.19 to 3.70)	2.76 (0.66 to 4.64)
Koeppen-Geiger Climatic Zones 2nd	0.74 (-0.33 to 1.72)	1.39 (-0.15 to 2.81)	2.03 (0.20 to 3.71)	2.81 (0.67 to 4.71)
Koeppen-Geiger Climatic Zones 3rd	0.76 (-0.37 to 1.79)	1.39 (-0.17 to 2.83)	2.04 (0.18 to 3.75)	2.83 (0.59 to 4.82)
GDP per capita ($)	0.72 (-0.34 to 1.72)	1.35 (-0.16 to 2.77)	2.00 (0.27 to 3.60)	2.75 (0.74 to 4.55)
Latitude	0.72 (-0.33 to 1.72)	1.36 (-0.17 to 2.80)	1.98 (0.26 to 3.58)	2.73 (0.76 to 4.50)
longitude	0.70 (-0.32 to 1.66)	1.35 (-0.13 to 2.72)	1.99 (0.31 to 3.55)	2.72 (0.73 to 4.51)

Definition of abbreviations: BLUP = the best linear unbiased prediction; TV = temperature variability; Q1 = the first quartile; Q2 = the second quartile; Q3 = the third quartile; Q4 = the fourth quartile.

Table S14. Sensitivity analyses of overall attributable fractions after changing the lag of mean temperature in the main model, stratified by quartiles of TV.

Lag	Attributable fractions (%)			
	Q1 of TV	Q2 of TV	Q3 of TV	Q4 of TV
7	0.80 (-0.34 to 1.83)	1.56 (-0.05 to 3.03)	2.24 (0.51 to 3.84)	2.99 (1.01 to 4.78)
8	0.78 (-0.34 to 1.80)	1.48 (-0.06 to 2.90)	2.14 (0.42 to 3.74)	2.87 (0.93 to 4.61)
9	0.72 (-0.35 to 1.72)	1.39 (-0.11 to 2.80)	2.07 (0.35 to 3.67)	2.81 (0.82 to 4.61)
10 [a]	0.70 (-0.33 to 1.69)	1.34 (-0.14 to 2.73)	1.99 (0.29 to 3.57)	2.73 (0.76 to 4.50)
11	0.67 (-0.34 to 1.62)	1.28 (-0.16 to 2.64)	1.92 (0.25 to 3.46)	2.62 (0.67 to 4.37)
12	0.64 (-0.37 to 1.58)	1.24 (-0.21 to 2.60)	1.86 (0.19 to 3.41)	2.54 (0.58 to 4.30)
13	0.62 (-0.36 to 1.55)	1.20 (-0.25 to 2.57)	1.80 (0.13 to 3.35)	2.47 (0.52 to 4.21)

Definition of abbreviations: TV = temperature variability; Q1 = the first quartile; Q2 = the second quartile; Q3 = the third quartile; Q4 = the fourth quartile.

[a] lag day used in the primary model.

Table S15. Sensitivity analyses of overall attributable fractions using different definitions of warm season, stratified by quartiles of TV.

Warm season	Attributable fractions (%)			
	Q1 of TV	Q2 of TV	Q3 of TV	Q4 of TV
two consecutive months	1.63 (-1.33 to 4.32)	2.28 (-1.21 to 5.33)	3.06 (-0.80 to 6.48)	4.69 (0.31 to 8.49)
three consecutive months	1.00 (-0.38 to 2.37)	1.53 (-0.20 to 3.17)	2.26 (0.14 to 4.19)	3.13 (0.75 to 5.32)
four consecutive months [a]	0.70 (-0.33 to 1.69)	1.34 (-0.14 to 2.73)	1.99 (0.29 to 3.57)	2.73 (0.76 to 4.50)

Definition of abbreviations: TV = temperature variability; Q1 = the first quartile; Q2 = the second quartile; Q3 = the third quartile; Q4 = the fourth quartile.

[a] warm season used in the primary model.

Table S16. Sensitivity analyses of overall attributable fractions after adjusting relative humidity in the main model using 556 cities with relative humidity data, stratified by quartiles of TV.

Model	Attributable fractions (%)			
	Q1 of TV	Q2 of TV	Q3 of TV	Q4 of TV
Without relative humidity	0.68 (-0.26 to 1.56)	1.34 (-0.05 to 2.64)	2.00 (0.44 to 3.46)	2.66 (0.99 to 4.17)
With relative humidity	0.73 (-0.29 to 1.69)	1.41 (-0.03 to 2.74)	2.08 (0.49 to 3.55)	2.73 (0.96 to 4.32)

Definition of abbreviations: TV = temperature variability; Q1 = the first quartile; Q2 = the second quartile; Q3 = the third quartile; Q4 = the fourth quartile.

Table S17. Sensitivity analyses of overall attributable fractions using different complete data after removing cities with missing vlaues or after imputaion, stratified by quartiles of TV.

Dataset	Attributable fractions (%)			
	Q1 of TV	Q2 of TV	Q3 of TV	Q4 of TV
complete data after removing cities with missing values	0.59 (-0.41 to 1.51)	1.10 (-0.30 to 2.41)	1.69 (0.14 to 3.11)	2.65 (0.70 to 4.37)
complete data after imputation	0.71 (-0.39 to 1.73)	1.35 (-0.14 to 2.75)	1.99 (0.28 to 3.57)	2.73 (0.79 to 4.48)
primary results	0.70 (-0.33 to 1.69)	1.34 (-0.14 to 2.73)	1.99 (0.29 to 3.57)	2.73 (0.76 to 4.50)

Definition of abbreviations: TV = temperature variability; Q1 = the first quartile; Q2 = the second quartile; Q3 = the third quartile; Q4 = the fourth quartile.

Reference:

1. Zhao Q, Guo Y, Ye T, et al. Global, regional, and national burden of mortality associated with non-optimal ambient temperatures from 2000 to 2019: a three-stage modelling study. *The Lancet Planetary health* 2021; **5**(7): e415-e25.
2. Xu R, Xiong X, Abramson MJ, Li S, Guo Y. Ambient temperature and intentional homicide: A multi-city case-crossover study in the US. *Environ Int* 2020; **143**: 105992.
3. Meng X, Liu C, Chen R, et al. Short term associations of ambient nitrogen dioxide with daily total, cardiovascular, and respiratory mortality: multilocation analysis in 398 cities. *BMJ (Clinical research ed)* 2021; **372**: n534.
